# Antioxidant, Alpha-Glucosidase Inhibition Activities, In Silico Molecular Docking and Pharmacokinetics Study of Phenolic Compounds from Native Australian Fruits and Spices

**DOI:** 10.3390/antiox12020254

**Published:** 2023-01-23

**Authors:** Akhtar Ali, Jeremy J. Cottrell, Frank R. Dunshea

**Affiliations:** 1 School of Agriculture and Food, The University of Melbourne, Parkville, VIC 3010, Australia; 2 Faculty of Biological Sciences, The University of Leeds, Leeds LS2 9JT, UK

**Keywords:** mountain pepper, rosella, strawberry gum, lemon aspen, flavonoids, anthocyanins, bioavailability, LC-MS/MS

## Abstract

Native Australian fruits and spices are enriched with beneficial phytochemicals, especially phenolic compounds, which are not fully elucidated. Therefore, this study aimed to analyze native Australian mountain-pepper berries (*Tasmannia lanceolata*), rosella (*Hibiscus sabdariffa*), lemon aspen (*Acronychia acidula*), and strawberry gum (*Eucalyptus olida*) for phenolic and non-phenolic metabolites and their antioxidant and alpha-glucosidase inhibition activities. Liquid chromatography–mass spectrometry–electrospray ionization coupled with quadrupole time of flight (LC-ESI-QTOF-MS/MS) was applied to elucidate the composition, identities, and quantities of bioactive phenolic metabolites in Australian native commercial fruits and spices. This study identified 143 phenolic compounds, including 31 phenolic acids, 70 flavonoids, 10 isoflavonoids, 7 tannins, 3 stilbenes, 7 lignans, 10 other compounds, and 5 limonoids. Strawberry gum was found to have the highest total phenolic content (TPC—36.57 ± 1.34 milligram gallic acid equivalent per gram (mg GAE/g), whereas lemon aspen contained the least TPC (4.40 ± 0.38 mg GAE/g). Moreover, strawberry gum and mountain pepper berries were found to have the highest antioxidant and anti-diabetic potential. In silico molecular docking and pharmacokinetics screening were also conducted to predict the potential of the most abundant phenolic compounds in these selected plants. A positive correlation was observed between phenolic contents and biological activities. This study will encourage further research to identify the nutraceutical and phytopharmaceutical potential of these native Australian fruits.

## 1. Introduction

Diabetes mellitus is one of the leading causes of death around the globe [1] and is characterized by high blood glucose levels. Alpha-glucosidase (α-glucosidase) is the main enzyme with a significant role in hydroxylation, digestion, and absorption of sugars in the human body. Therefore, inhibiting α-glucosidase is an effective strategy for treating and minimizing type 2 diabetes. There is increasing interest in using natural sources to treat diabetes. Various nutraceuticals and bioactive compounds have been investigated to control/inhibit the complications of diabetes. Using phenolic metabolites is a therapeutic approach to suppressing the prevalence of pre- or post-diabetic conditions [1]. Therefore, detailed characterization and identification of phenolic metabolites is required to understand the potent role of polyphenols in food and human health.

Fruits, vegetables, herbs, spices, and medicinal plants contain large amounts of phytochemicals, including polyphenols. When they encounter living tissues, they exhibit a beneficial effect on human health [1]. Flavonoids are the largest subclass of polyphenols, with more than ten thousand compounds being reported [2]. According to nutritionists, foods high in polyphenols may reduce or remove the risk for certain malignancies, degenerative diseases, cardiovascular ailments, and chronic inflammation in humans [3]. Excessive production of reactive oxygen species (ROS) and reactive nitrogen species (RNS) leads to oxidative stress in the body, the leading cause of the above-mentioned pathological conditions [4]. Tasmannia lanceolata, commonly known as the mountain pepper berry, is used in food flavoring and traditional medicine to treat venereal diseases, skin disorders, and stomachaches [5]. Lemon aspen is a pale/yellow, 2–2.5 cm in diameter fruit that is endemic to Queensland, Australia. It is traditionally used in curries and on meats as a seasoning, though syrups, sauces, and infused vinegar are also made from lemon aspen [6]. Strawberry gum and rosella are also used in traditional medicine.

Considering that bioactive phenolic compounds have strong antioxidant and antidiabetic potential, it was hypothesized that selected native Australian plants could have considerable bioactive potential. In this context, we comprehensively characterized native Australian mountain pepper berries, rosella, lemon aspen, and strawberry gum for their phenolic compounds’ antioxidant and α-glucosidase inhibition potential. Previously, these native Australian plants had not been studied for radical scavenging and α-glucosidase inhibition activities. Therefore, total monomeric anthocyanin content (TMAC), total phenolic content (TPC), total condensed tannins (TCT), and total flavonoid content (TFC) were measured. Furthermore, antioxidant activities, through the ferrous ion chelating assay (FICA), ferric reducing antioxidant power (FRAP) test, 2,2′-diphenyl-1-picrylhydrazyl (DPPH) reducing power assay (RPA), 2,2′-azinobis-(3-ethylbenzothiazoline-6-sulfonic acid (ABTS) assay, phosphomolybdate assay (PMA), and hydroxyl-radical scavenging assay (^•^OH-RSA), were also measured in this study. The anti-diabetic potential of these selected native Australian plants was also measured through the α-glucosidase inhibition activity. Moreover, LC-ESI-qTOF-MS/MS was used to characterize and screen polyphenols from these native Australian plants. Furthermore, the binding affinities of most abundant phenolic metabolites in selected native Australian plants for the active sites of a-glucosidase (5NN8) were predicted using the in silico molecular docking. Nowadays, in silico molecular docking is widely used in drug discovery, which enables us to understand the behavior of drug molecules in the binding sites of the α-glucosidase protein and explains the basic biochemical processes [7,8,9]. Moreover, oral bioavailability, drug-likeness, absorption, distribution, metabolism, excretion, and toxicity of abundant phenolic compounds were computed to predict their suitability as therapeutic agents. This research explores the use of native Australian plants in the medicinal, pharmaceutical, food, and feed industries.

## 2. Materials and Methods

### 2.1. Chemicals and Reagents

Analytical, HPLC, and LCMS-grade chemicals were used as described [1,10].

### 2.2. Preparation and Extraction of Phenolic Compounds

Mountain pepper berries (whole dried), rosella (freeze-dried powder), and strawberry gum (finely ground) were purchased from the Australian superfoods Company (www.australiansuperco.com.au) accessed on 21 September 2021. Lemon aspen (freeze-dried powder) was purchased from Australian Creative Native Foods (www.creativenativefoods.com.au) accessed on 21 September 2021. The bioactive phenolic compounds from the selected native Australian fruits were extracted in triplicate by following the method of Ali et al. [1]. The extracts were stored at −20 °C, and all the analyses were conducted within a week.

### 2.3. Measurement of Phenolic Contents and Biological Activities

The TPC of selected native Australian plants was examined by following the method of Ali et al. [1]. A 25 μL sample extract or standard, 25 μL Folin-Ciocalteu reagent (25% in Milli-Q water) and 200 μL of H_2_O were mixed in a 96-well plate and incubated for 5 min at room temperature. Then, 25 μL of 10% sodium carbonate was mixed and again incubated for 60 min at room temperature in the dark. Gallic acid monohydrate (≥99%) in analytical grade ethanol (0–200 μg/mL) was used to generate standard curve at 765 nm. Then, the method of Sharifi-Rad et al. [11] was used to quantify the TFC of native Australian fruits and spices. The TCT and TMAC of selected plants were determined using the procedures of Ali et al. [1,10]. The DPPH and ABTS activities were measured using the methods of Chou et al. [12] and Zahid et al. [13]. The PMA, RPA and FRAP potential of these selected plants were quantified by adopting the methods of Ali et al. [10]. The FICA and the ^•^OH-RSA potential of selected plants were quantified by adopting the methods of Bashmil et al. [14] and Ali et al. [10]. α-Glucosidase inhibition activity was determined by following our previously published method [1], and acarbose (Aca) was used as a reference drug (≥95%).

### 2.4. LC-MS/MS Analysis

LC-ESI-Q-TOF-MS/MS was used to analyze the untargeted phenolic metabolites from native Australian mountain pepper berries, rosella, strawberry gum, and lemon aspen by following the methods of Ali at al. [1,15]. The heatmap hierarchical clustering was conducted by using MetaboAnalyst 5.0 (www.metaboanalyst.ca) accessed on 7 November 2022.

### 2.5. Molecular Docking and Pharmacokinetic Properties of Abundant Phenolic Compounds

The pharmacokinetic properties of the most abundant phenolic compounds tentatively identified in the plants were predicted by following the methods of Ali et al. [16] and Daina et al. [17]. Oral bioavailability, absorption, distribution, metabolism, excretion, and toxicity of the abundant phenolic compounds were predicted. Moreover, in silico molecular docking was also conducted to predict the α-glucosidase potential of the selected phenolic compounds from native Australian fruits and spices, as described by Ali et al. [1]. Grid box dimensions were x = −12.95, y = −36.99, and y = 87.77 while docking ligands with a length lower than 20 Å.

### 2.6. Statistical Analysis

Minitab (version 18.0, Minitab, LLC, State College, PA, USA) and XLSTAT-2019.1.3 software were used for and analysis of variance (ANOVA), Pearson correlation, and a biplot analysis. The results of phenolic contents and their biological activities are represented as mean ± standard deviation.

## 3. Results and Discussion

### 3.1. Measurement of Total Polyphenols (TPC, TFC, TMAC, TCT)

Phytochemicals, especially plants’ secondary metabolites, are vital for human health [18]. Phenolic acids and flavonoids are critical secondary bioactive metabolites with various health benefits. They are considered multi-functional metabolites, as metal chelators, hydrogen atom donators, free radical scavengers, and reducing agents [18].

In this study, we investigated Australian mountain pepper berries, rosella, lemon aspen, and strawberry gum for phenolic and non-phenolic compounds. TPC, TFC, TCT, and TMAC quantified in these native Australian plants are given in Table 1.

Total phenols represent phenolic acids, flavonoids, stilbenes, lignans, coumarins and derivatives, tyrosols, and other small molecules. In this context, strawberry gum was found to have the highest TPC (36.57 ± 1.34 mg GAE/g) of the selected Australian native plants. The TPC of strawberry gum was comparable to the previously quantified TPC of Australian-grown thyme (43.16 ± 1.54 mg GAE/g), basil (39.91 ± 1.39 mg GAE/g), and allspice (40.49 ± 1.92 mg GAE/g) [10,18]. Previously, the levels of phenolic compounds in Australian native lemon myrtle and Tasmanian pepper berry were found to be in the range of 16.9 to 31.4 mg GAE/g [19]. Moreover, the TPC of mountain pepper berries was comparable to the TPC reported by Cáceres-Vélez et al. [20] and Vélez et al. [21]. The concentrations of phenolic contents in mountain pepper berry, rosella, and lemon aspen are 2 to 3-fold higher than in Australian-grown cherries [22]. Previously, Lukmanto et al. [23] also measured the TPC of 8.63 mg GAE/g, which is comparatively higher than our results. The TPC of strawberry gum is also comparable to that of villous amomum fruit (46.02 ± 1.12 mg GAE/g) and that of citron fruit (46.22 ± 1.01 mg GAE/g) reported by Liu et al. [24].

The highest TFC (15.69 ± 2.69 mg QE/g) was quantified in strawberry gum, and the lowest TFC (0.79 ± 0.04 mg QE/g) was quantified in lemon aspen. The highest TCT (8.05 ± 0.52 mg CE/g) was also measured in strawberry gum, and the lowest TCT (1.26 ± 1.13 mg CE/g) was measured in rosella. The TMAC was only measured in mountain pepper berry (0.17 ± 0.03 mg/g) and rosella (0.08 ± 0.02 mg/g). Previously, we measured higher amounts of total anthocyanins in the Davidson plum and quandong peach than berries [1]. Flavonoids are the most abundant class of phenolic compounds in fruits, herbs, and medicinal plants, and they have gained much interest due to their health properties. Previously, a limited number of studies have been conducted to investigate the total flavonoid contents in these plants. There are significant differences in total phenolics, and flavonoids found in each study conducted on these plants due to the aforementioned factors.

### 3.2. Biological Activities of Native Australian Fruits and Spices

Phenolic compounds include diverse antioxidant constituents present in plants that have various health effects. According to several studies, certain plants’ antioxidant properties vary due to their diverse bioactive components and mostly depend on the extraction technique and method used to quantify them. Numerous studies have been carried out to estimate the antioxidant activities of plants from different geographical locations [25,26,27,28,29,30], but the information on native Australian plants is limited. Therefore, we conducted various antioxidant activity tests to understand the targeted antioxidant potential of native Australian fruits. Various antioxidant activity tests should help in understanding the potential of these native Australian herbs and medicinal plants.

**Figure 1:**
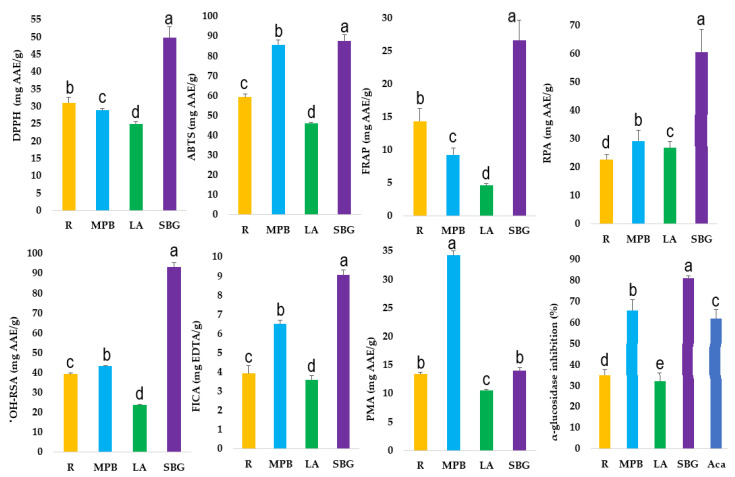
Biological activities (2,2′-diphenyl-1-picrylhydrazyl (DPPH), 2,2′-azinobis-(3-ethylbenzothiazoline-6-sulfonic acid (ABTS), ferric reducing antioxidant power (FRAP), reducing power assay (RPA), hydroxyl-radical scavenging assay (^•^OH-RSA), ferrous ion chelating assay (FICA), phosphomolybdate assay (PMA)) and α-glucosidase inhibition activity of native Australian rosella (R), mountain pepper berries (MPB), lemon aspen (LA), and strawberry gum (SBG); acarbose (Aca). The vales with letters (a–e) are significantly different from each other.

In this study, seven in vitro antioxidant assays (DPPH, ABTS, FRAP, RPA, ^•^OH-RSA, FICA, and PMA) were conducted, and α-glucosidase inhibition activity was tested, to measure the antioxidant and anti-diabetic potential of native Australian native mountain pepper berries, rosella, strawberry gum, and lemon aspen (Table S1, Figure 1).

DPPH and ABTS are the widely used in vitro antioxidant assays for total antioxidant potential measurement of plant extracts based on scavenging the free radicals in the biological system. The highest DPPH (49.70 ± 3.21 mg AAE/g) was measured in strawberry gum, and the lowest DPPH (24.94 ± 0.70 mg AAE/g) was quantified in sea lemon aspen. ABTS^+^ radical cation inhibition is based on the characteristic wavelength of 734 nm [31]. The ABTS values of strawberry gum (87.65 ± 3.17 mg AAE/g) and mountain pepper berries (85.60 ± 2.32 mg AAE/g) were higher than those of rosella (59.27 ± 1.50 mg AAE/g) and lemon aspen (46.18 ± 0.38 mg AAE/g). Some other studies also reported higher ABTS values for rosemary, oregano, and mint [18]. This indicates that strawberry gum has a higher antioxidant potential than mountain pepper berries, rosella, and lemon aspen. The Fe^+3^–TPTZ complex, which reduces the antioxidant compounds’ ability to form an Fe^+2^–TPTZ complex in the biological system, was evaluated through the FRAP assay [10]. The FRAP values of strawberry gum (26.57 ± 3.10 mg AAE/g) and rosella (14.30 ± 1.92 mg AAE/g) were higher than those of the other selected fruits and spices (*p* < 0.05). Previously, the highest FRAP values were found in rosemary (17.21 ± 0.54 mg AAE/g) and oregano (10.72 ± 1.44 mg AAE/g). Fenugreek was found to have the lowest value of FRAP (1.48 ± 1.21 mg AAE/g). Furthermore, Wojdyło et al. [32] also reported higher FRAP for rosemary than the other plants selected in our study.

Excessive amounts of different reactive oxygen species (ROS), such as hydrogen peroxide (H_2_O_2_), hydroxy radicals (^•^OH), and super-oxide radicals (O_2_^•^⁻), cause various pathologies. The ^•^OH radicals cause lipid peroxidation and DNA damage due to high oxidative stress, and the daily consumption of antioxidant-rich fruits is crucial in order to protect the human body from these pathologies [18]. The highest ^•^OH-RSA value (93.29 ± 2.20 mg AAE/g) was achieved by strawberry gum. This is vital because ^•^OH scavenging inhibits lipid peroxidation by inhibiting the transition of oxidized metal ions [33,34]. The metal chelating ability of native Australian fruits and spices was estimated by using the ferrous ion chelating assay (FICA), and the highest FICA result (9.05 ± 0.27 mg EDTA/g) was achieved by strawberry gum. The principal antioxidant ingredients are flavonoids, according to a significant association between the antioxidant properties and flavonoids. These fruits and spices can contain different reducing agents which can bind with free radicals to terminate or stabilize the chain reactions in the biological systems [35]. Thus, high reduction power for a fruit extract indicates high antioxidant capacity. Free radicals can be produced by metabolic processes within bodily tissues and brought from outside sources such as food, medications, and pollution. Natural antioxidants are increasingly being used as food additives to neutralize free radicals. This is due to their scavenging abilities and the fact that they are all-natural, non-synthetic items that are well-liked by consumers. Furthermore, α-glucosidase inhibition activity of strawberry gum (81.01 ± 4.6 %) and mountain pepper berries (65.78 ± 5.01 %) was quantified higher than acarbose (standard), rosella, and lemon aspen. Previously, Syabana et al. [36] quantified the IC_50_ of *Cosmos caudatus* (61.33 ± 1.21 μg/mL), *Etlingera elaitor* (53.13 ± 2.87 μg/mL), *Pluchea indica* (12.17 ± 0.18 μg/mL), and *Syzygium polyanthum* (11.76 ± 0.32 μg/mL). The inhibitory activity of strawberry gum (12.01 ± 1.2 μg/mL) was higher than the activity of *Cosmos caudatus* and *Etlingera elaitor* and comparable to the activity of *Pluchea indica* and *Syzygium polyanthum* (Table S1). The inhibitory activity levels of rosella (79.09 ± 7.52 μg/mL) and lemon aspen (83.07 ± 9.03 μg/mL) were lower than those of these plants (Table S1). Moreover, the inhibitory activity of strawberry gum was comparable to that of the methanolic extract of *Satureja cuneifolia* (10.66 μg/mL) reported by Taslimi et al. [37].

Fruits, herbs, spices, and medicinal plants are used as antioxidant sources in the human diet because they inhibit or deactivate the free radicals in the body [38]. Generally, phenolic molecules are regarded as the active antioxidant components in fruits, herbs, and medicinal plants, thereby having potent health benefits. They act as metal chelators, anti-radicals, hydrogen-ion donators, and reducing agents in the biological system [10]. It has been reported that there are many methods to measure a plant extract’s total antioxidant potential due to the diverse nature of antioxidant compounds, mainly phenolic constituents [1,18]. The plant’s bioactive compounds, mainly polyphenols, depend on the type of cultivar, area, and climatic conditions. There are several techniques to assess the antioxidant capacities of bioactive phenolic metabolites, each with its advantages and disadvantages [11,14,19]. Generally, no approach measures the exact antioxidant capacity of bioactive phenolic compounds because of the complexity of phenolic compounds and the variety of processes of reactions in the human body [39]. These results demonstrate that further research is required to identify and quantify the individual phenolic compounds in these selected native Australian plants. Thus, LC-MS/MS was used to elucidate plant extracts’ structure, composition, and bioactive metabolites. The proper quantification and identification of individual phenolic compounds via the process of LC-MS/MS in these plants might help make the essential role of these bioactive metabolites in antioxidant activities understandable.

### 3.3. Correlation Analysis

Correlation analysis was executed between the phenolic contents (TPC and TFC) of the Australian native herbs and their antioxidant activities generated by the eight different assays (Table 2).

It is observed that a positive correlation (*p* ≤ 0.1) of TPC was observed with the TFC (*r* = 1.00), TCT (*r* = 0.99), FRAP (*r* = 0.92), ^•^OH-RSA (*r* = 0.97), and RPA (*r* = 0.99); the TFC had a significant positive correlation with TCT (*r* = 0.99), DPPH (*r* = 0.98), FRAP (*r* = 0.92), ^•^OH-RSA (*r* = 0.97), and RPA (*r* = 0.99). This appears to show a direct association between the phenolic compounds in the strawberry gum and the antioxidant processes of peroxyl inhibition, ferric chelation, and free radical scavenging. There was a strong correlation of flavonoids with hydroxyl inhibition, but there were lesser ones with the free radical scavenging, the phosphomolybdate assay outcome, and ferric ion chelation activity. This also indicates the diversity of phenolic and non-phenolic metabolites present in the extracts of native Australian plants. This may be connected to the fact that the flavonoid’s ability to operate as an antioxidant often depends on where the hydroxyl group is located on the B-ring and whether it can provide a free radical, either a hydrogen or an electron [1]. Additionally, the experimental conditions, the mechanisms of the antioxidant reactions, and the synergistic or antagonistic effects of various compounds present in the reaction mixture can all impact the associations between antioxidant activity and phenolic compounds [15,18].

**Figure 2:**
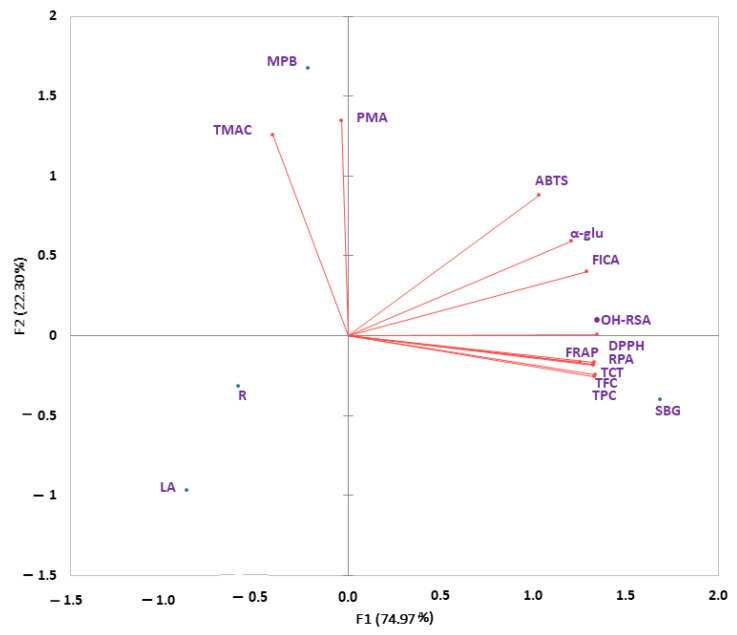
Biplot analysis of phenolic contents and biological activities in native Australian lemon aspen (**LA**), rosella (R), strawberry gum (**SBG**), and mountain pepper berries (**MPB**).

A biplot (Figure 2) exhibits that the higher TPC, TFC, and TCT in strawberry gum significantly contributed to all antioxidant activities except that shown by the phosphomolybdate assay. Furthermore, it is depicted that mountain pepper berries have higher concentrations of total monomeric anthocyanin than other plants. Interestingly, rosella and lemon aspen are negatively correlated with all biological activities, which indicates that these have low concentrations of phenolic compounds and flavonoids. Previously, it has been demonstrated that a greater number of OH groups in a flavonoid is favorable for biological activities. Furthermore, each ring’s structural arrangement its number of hydroxyl groups, a catechol group in the B ring, and several double bonds in the C ring determine the antioxidant capacity of phenolic metabolites in extracts [40]. Numerous investigations of herbs and medicinal plants have shown a significant, positive association between phenolic content and antioxidant activity [41]. Previously, we reported a positive correlation between phenolic contents of herbs and spices and their antioxidant activities [10,18]. Additionally, two other studies showed that phenolic contents in native Australian fruits and other plants also had positive relationships with their biological activities [1,15].

### 3.4. LC-MS Analysis

Nutritionists and food scientists have concentrated on exploring the thorough characterization of fruits, spices, and medicinal plants in response to the growing interest in and understanding the antioxidant potential and associated health benefits of phenolic chemicals. The untargeted characterization and screening of phenolic compounds from Australian native fruits and medicinal plants (mountain pepper berries, strawberry gum, rosella, and lemon aspen) were achieved using LC-ESI-QTOF-MS/MS. In this context, a total of 143 phenolic and non-phenolic metabolites, including 31 phenolic acids, 70 flavonoids, 10 isoflavonoids, 7 tannins, 3 stilbenes, 7 lignans, 10 other compounds, and 5 limonoids, were tentatively characterized through the analysis of their MS/MS spectra (Table 3, Figure S1, Figure S2).

#### 3.4.1. Phenolic Acids

Phenolic acids are diverse plant metabolites from the secondary class produced via the phenylpropanoid pathway by shikimic acid [42]. They are broadly utilized in beauty, health, pharmacology, and medicinal industries due to their anti-aging, antioxidant, antimicrobial, anti-cancer, cardio-protective, antitumor, and anti-inflammatory properties [43].

##### Benzoic acid and Its Derivatives

Benzoic acid is the simplest aromatic carboxylic acid and has a range of derivatives. Eight hydroxybenzoic acids in total were tentatively identified in these native Australian plants. Compounds **1**, **3**, and **8** produced fragment ions at *m*/*z* 169, 153, and 137 after the loss of the glycosyl moiety [M−H−162]^−^ from the precursor ions, respectively. Compounds **1**, **3**, and **8** were tentatively identified as gallic acid 4-*O*-glucoside, protocatechuic acid 4-*O*-glucoside, and 4-hydroxybenzoic acid 4-*O*-glucoside, respectively. Compounds **2**, **4**, and **5** generated product ions at *m/z* 125, 109, and 93 after the loss of CO_2_ (44 Da) from the parent ions, respectively [10]. Compounds **2**, **4**, and **5** were identified through pure standards as gallic acid, protocatechuic acid, and *p*-hydroxybenzoic acid. Compound **2** (gallic acid—C_7_H_6_O_5_) and compound **4** (protocatechuic acid—C_7_H_6_O_4_) were identified in mountain pepper berries and strawberry gum. Gallic acid 4-*O*-glucoside was only identified in mountain pepper berries, whereas benzoic acid (compound 4) was identified in all four fruits. The presence of the unique phenolic acid protocatechuic acid has been reported in many therapeutic plants [44]. It has said to possess several health benefits, including anti-inflammatory, antioxidant, anti-cancer, anti-ulcer, anti-diabetic, hepato-protective, and neuro-protective activities [44].

##### Cinnamic Acids and Derivatives

The most prevalent phenolic acid class is hydroxycinnamic in fruits, herbs, and medicinal plants. Sixteen hydroxycinnamic acids were identified, and their fragmentation patterns were verified using MS/MS. The removal of CO_2_ and the hexosyl moiety from the parent ions is the primary way that phenolic acids exhibit the fragmentation pattern [10]. Rosmarinic acid, caffeic acid, sinapic acid, *p*-coumaric acid, 3-caffeoylquinic acid (chlorogenic acid), and cinnamic acid were confirmed through pure standards. The quinic acid derivatives **9** (*m*/*z* 349.0921), **20** (*m*/*z* 337.0934) **21** (*m*/*z* 367.1025), **22** (*m*/*z* 397.1141), and **29** (*m*/*z* 515.1197) are known as -feruloyquinic acid lactone, 3-*p*-coumaroylquinic acid, 3-feruloylquinic acid, 3-sinapoylquinic acid, and 1,5-dicaffeoylquinic acid, respectively. Compound 21 (3-feruloylquinic acid) was tentatively identified in rosella and lemon aspen at *m*/*z* 367.1029, which generated characteristic fragment ions of ferulic acid and quinic acid at *m*/*z* 193 and 191 in negative-ion mode. Compound **29** (1,5-dicaffeoylquinic acid) was detected in rosella and strawberry gum in negative mode. This was confirmed through MS/MS, where it produced fragment ions at *m*/*z* 353, 191, and 179 after the breakdown of precursor ions into 5-caffeoylquinic acid (*m*/*z* 353), quinic acid (*m*/*z* 191), and caffeic acid (*m*/*z* 179) units, respectively [10]. Previously, 1,5-dicaffeoylquinic acid was also identified in cumin [10]. Compound **10** at ESI^−^ *m*/*z* 279.0503 was identified in lemon aspen and strawberry gum, which generated a product ion at *m*/*z* 163 (coumaric acid) after the loss of C_4_H_4_O_4_ (116 Da) from the precursor ion in the MS/MS scan. Therefore, compound **10** was putatively identified as *p*-coumaroyl malic acid. Rosmarinic acid produced a characteristic fragment at *m*/*z* 197 after the removal of a hexose moiety (162 Da), which further broke down into a caffeic acid unit (*m*/*z* 179) through the loss of H_2_O, and a caffeic acid (*m*/*z* 179) fragment at *m*/*z* 135 represents the loss of a CO_2_ [M−H−44]^−^ unit [45,46]. Rosmarinic acid (compound **14**) is one of herbs’ and medicinal plants’ most plentiful phenolic acids. Compound **24** at ESI^−^ *m*/*z* 163.0400 was identified in mountain pepper berries, rosella, and lemon aspen, which generated a product ion at *m*/*z* 119 after the loss of CO_2_ [M−H−44]^−^ from the precursor ion (*m*/*z* 163.0400). Compound **24** was tentatively identified as *p*-coumaric acid. Compound **31** at ESI^+^ *m*/*z* 223.0605 generated product ions at *m*/*z* 205 and 147, and 119, after the loss of a unit of H_2_O (18 Da), the glycolic acid moiety (76 Da), and both from the precursor ion, respectively. In contrast, a product ion at *m*/*z* 119 is a specific fragment ion of *p*-coumaric acid. As a result, compound **31** was tentatively identified as *p*-coumaroyl glycolic acid in rosella and mountain pepper berries. Previously, Kadam et al. [47] also reported *p*-coumaroyl glycolic in *Lepidium sativum* seedcake.

#### 3.4.2. Flavonoids

Flavonoids are widely used in nutraceutical, pharmaceutical, and cosmetic industries due to their anti-carcinogenic, antimicrobial, anti-inflammatory, anti-mutagenic, and antioxidant properties. In this study, we tentatively identified seventy flavonoids (Table 2).

##### Anthocyanins

Anthocyanins are water-soluble, colored plant pigments. The main positions of their hydroxyls are 3, 5, and 7 in ring A and 3′ and 5′ in ring B [48]. The screening, identification, and characterization of anthocyanins in native Australian rosella and mountain pepper berries were conducted. This work identified nine anthocyanins using their MS/MS spectra (Table 2). The native Australian quandong peach and Davidson plum were used as reference plants to understand anthocyanins’ structural and spectral characteristics further; these fruits are abundant in anthocyanins [1]. The removal of sugar units from anthocyanins (162 Da for hexoses, 150 Da for xyloses, 132 Da for pentoses, and 308 Da for the rutinoside moiety from the basic aglycone of corresponding anthocyanins) results in the formation of MS/MS product ions (303 Da for delphinidin, 331 Da for malvidin, 301 Da for peonidin, 317 Da for petunidin, and 287 Da for cyanidin) [1]. Compounds **33**, 35, and **36** at ESI^+^ *m*/*z* 581.1526, 595.1660, and 449.0994 generated a characteristic fragment ion at *m*/*z* 287 (cyanidin). Thus, compounds **33**, **35**, and **36** were tentatively identified as cyanidin 3-sambubioside, cyanidin 3-rutinoside, and cyanidin-3-O-glucoside, respectively. Compound **36** (cyanidin 3-*O*-glucoside) was identified in mountain pepper berries and rosella. Cyanidin-3-*O*-glucoside was quantified in grapes from 2.7 to 51.7 μg/mL by Oh et al. [49]. Compounds **32**, **38**, **39**, **40**, and **41** produced a distinctive fragment of delphinidin at *m*/*z* 303 in positive-ion mode (Table 2). Compounds **33**, **38**, and **39** were only identified in rosella. Due to their positively charged oxygen atom, anthocyanins have higher antioxidant activity than other flavonoids [50].

##### Flavanols

We identified monomeric flavanols in our samples, including epicatechin, epigallocatechin, and derivatives [48]. In this study, seven flavanols, including polymerized and derivative substances, were tentatively identified in mountain pepper berries, rosella, strawberry gum, and lemon aspen. Flavanols are also called catechins, having no double bond between C2 and C3, and there is no carbonyl group in ring C (C4) [51]. Compound **44** at ESI^−^ *m*/*z* 305.0650 generated product ions at *m*/*z* 289, 169, and 125 from the ion precursor. Compound **44** was putatively identified as (−)-epigallocatechin (C_15_H_14_O_7_). They have been reported abundantly for their potent antioxidant and cardio-protective effects in tea and cocoa. Compound **46** at ESI^−^ *m*/*z* 289.0711 was identified in strawberry gum, rosella, and mountain pepper berries, which produced product ions at *m*/*z* 245, 205, and 179 after CO_2_ loss [M−H−44]^−^, flavonoid A ring [M−H−84]^−^ loss, and flavonoid B ring [M−H−110]^−^ loss from the precursor ion, respectively. Compound **46** was tentatively identified as epicatechin (C_15_H_14_O_6_) [52]. These compounds are the building blocks of proanthocyanidins (condensed tannins). The most prevalent flavonoids: flavanols, and flavan-3-ols have a variety of chemical and biological properties. 

##### Flavanones

Flavanones do not have double bond between C2 and C3, but they have a carbonyl ring at C4 in ring C [48]. Sixteen compounds were identified as flavanones. Compounds **49** (naringin 6′-malonate), **54** (6-geranylnaringenin), **56** (eriodictyol-7-*O*-glucoside), **59** (eriodictyol), **60** (naringenin), **62** (5,7-dihydroxyflavanone), **63** (Hesperidin), and 64 (3′,4′,5′-trimethoxyflavone) were only identified in strawberry gum; and compounds **52** (hesperetin 5-glucoside) and **58** (hesperetin 5′,7-*O*-diglucuronide) were only identified in lemon aspen. Compound **61** (8-Prenylnaringenin) was only identified in mountain pepper berries. Compounds **51**, **52**, **55**, and **56** generated product ions at *m*/*z* 579, 301, 271, and 287 after the loss of a glycosyl moiety from their precursor ions, respectively. Therefore, compounds **51**, **52**, **55**, and **56** were tentatively identified as narirutin 4′-*O*-glucoside, hesperetin 5-glucoside, naringenin-7-O-glucoside, and eriodictyol-7-*O*-glucoside, respectively.

##### Flavones and Isoflavones

Flavones are characterized by a non-saturated C3 chain and have a double bond between C2 and C3 [48]. Sixteen compounds were characterized as flavones and flavanones in mountain pepper berries, rosella, strawberry gum, and lemon aspen. Compounds **67** (velutin), **72** (biochanin A 7-*O*-glucoside), and **79** (chrysin) were only identified in strawberry gum; and compounds **69** (azaleatin 3-arabinoside), **73** (Apigenin 6-C-glucoside), **75** (apigenin), **75** (chrysoeriol 7-*O*-glucoside), **77** (wogonin), and **78** (glycitein) were only identified in mountain pepper berries. Compounds **68** (diosmin), **71** (luteolin), **74** (apigenin), **76** (diosmetin), and **79** (chrysin) were identified via the MS/MS spectra of pure standards. Compounds **70** (syringetin-3-*O*-glucoside), **72** (biochanin A 7-*O*-glucoside), **73** (apigenin 6-C-glucoside), and **75** (chrysoeriol 7-*O*-glucoside) generated product ions at *m*/*z* 299, 345, 271, and 299, respectively, after the loss of glycosyl moiety from their parent ions.

##### Flavonols, Dihydroflavonols, and Chalcones

Flavonols have a double bond between C2 and C3, and there is a carbonyl in ring C (C4) and a OH group at C3 [51]. These compounds have strong absorption at 340–380 nm. Eighteen compounds were identified as flavonols and dihydroflavonols. Compound **81** (limocitrin) was only identified in lemon aspen, and compounds **82** (myricetin 3-*O*-glucoside) and **97** (isorhamnetin 3-*O*-glucuronide) were only identified in rosella. Compounds **82** (*m*/*z* 479.0816), **92** (*m*/*z* 463.0842), and **94** (Kaempferol 3-*O*-glucoside) generated product ions at *m*/*z* **317** (myricetin), 301 (quercetin), and 285 (kaempferol) after the loss of a hexose moiety (162 Da) from the precursor ions, respectively. Compounds **82**, **92**, and **94** were putatively identified as myricetin 3-*O*-glucoside, quercetin-3-*O*-glucoside, and kaempferol 3-*O*-glucoside, respectively. Moreover, compounds **85**, **86**, **87**, **88**, **89**, **90**, **and 97** produced fragment ions at *m*/*z* 317 (myricetin), 285 (kaempferol), 301 (quercetin), 303 (dihydroquercetin), and 315 (isorhamnetin) after the loss of sugar moieties, including rhamnoside (146 Da), rutinoside (308 Da), arabinoside (132 Da), and glucuronide (176 Da), from their precursor ions. Compounds **83** (quercetin 3-(2-galloylglucoside) and **88** (quercitrin) were only identified in lemon aspen while compounds **80** (6-hydroxykaempferol 3,6-diglucoside 7-glucuronide), **86** (kaempferol 3-rutinoside), **87** (kaempferol 3-*O*-arabinoside), and **90** (quercetin 3-*O*-arabinoside) were only identified in mountain pepper berries. Compounds 84 (rutin), **92** (quercetin-3-*O*-glucoside), **93** (Isorhamnetin), **95** (myricetin), **96** (taxifolin), and **98** (quercetin) were identified through the MS/MS spectra of pure standards [53]. The resulting ions at *m*/*z* 300 and 271, which correspond to the loss of CH_3_ and CO_2_ from the precursor [1,18], were used to identify isorhamnetin (compound **93** at ESI^−^ *m*/*z* 315.0504), which was identified in mountain pepper berries, lemon aspen, and strawberry gum. In addition to repairing iron-induced DNA oxidation, myricetin 3-*O*-rhamnoside (compound **85**) also inhibits the activity of digestive, lipid, fecal, and colonic bacterial enzymes and functions as an anti-allergenic, anti-obesity, and anti-cancer compound [54]. Flavonols are also frequently found in Australian native fruits and medicinal plants. According to a comparison of the flavonoid literature, the aglycone derivatives of kaempferol, myricetin, and quercetin are the most often found flavonols in these plants. These aglycone derivatives are renowned for having highly effective anti-diabetic properties. These aglycone compounds are eight times more potent than the diabetic medication acarbose, according to some research [55]. In many earlier investigations, quercetin and kaempferol were connected to rutinoside, galactosides, and glucosides; previously, these flavonoid-3-*O*-glycosides were not described in selected native Australian plants. Three phenolic compounds, **99**, **100**, and **101**, were only identified in strawberry gum.

#### 3.4.3. Isoflavonoids

Isoflavonoids differ from flavonoids, as the isoflavonoid skeleton was biogenetically engineered from the 2-phenylchroman skeleton. In isoflavonoids, ring A (phenyl ring) is fused with the C-ring (six-membered heterocyclic ring) and another phenyl B-ring at C3, whereas the B-ring is substituted at C2 position in flavonoids [15]. Ten phenolic compounds were identified as isoflavonoids. Compounds **103** (equol 7-*O*-glucuronide) and **107** (3′,4′,7-trihydroxyisoflavanone) were only identified in strawberry gum; and **109** (daidzein 7-*O*-glucuronide) and **110** (3′-hydroxymelanettin) were only identified in rosella and lemon aspen. Compounds **103**, **106** and **109** generated product ions at *m*/*z* 241 (equol), 415 (daidzin), and 253 (daidzein) after the loss of [M−H−176] from their precursor ions, respectively. Compounds **103**, **106**, and **109** were tentatively identified as equol 7-*O*-glucuronide, daidzin 4′-*O*-glucuronide, and daidzein 7-*O*-glucuronide, respectively. As per our knowledge, no previous research has been conducted in such a comprehensive way to identify these isoflavonoids in the selected Australian native plants.

#### 3.4.4. Tannins

Proanthocyanidins (condensed tannins) are condensed flavanols. Seven compounds were identified as tannins (proanthocyanidins, hydrolyzable and complex tannins) [56]. Compound **115** at *m*/*z* 865.2004 produced fragment ions at *m*/*z* 739, 713, and 695 in negative-ion mode. The daughter ion at *m*/*z* 739 formed after the loss of ring “A” because of the fission of the heterocyclic ring [M−H−126]^−^ from the precursor ion, RDA (152 Da), and a water unit (18 Da) from the latter product ion (*m*/*z* 713). Compound **115** was putatively identified as the procyanidin trimer C1 in strawberry gum and rosella. Compound **116** at ESI^−^ *m*/*z* 577.1353 was tentatively identified in mountain pepper berries, lemon aspen, and strawberry gum, which generated fragment ions at *m*/*z* 451, 425, and 289; C4, C5 and O-C2 showed cleavage of one pyran ring, which led to phloroglucinol molecule loss (A-ring) from the precursor ion [52], which resulted in product ions at *m*/*z* 451 [M−H−126]^−^ and 425 [M−H−152]^−^. Compound **116** was putatively identified as the procyanidin B2. Previously, procyanidin B2 and procyanidin trimmer C1 were recognized in nutmeg and cinnamon [10]. They have been reported to have anti-cancer, antioxidant, cardio-protective, and anti-inflammatory activities [56,57]. Compounds **113** (2-*O*-galloylpunicalin) and **117** (punicafolin) were only identified in strawberry gum, and compound **114** (glucosyringic acid) was only identified in mountain pepper berries.

#### 3.4.5. Lignans and Stilbenes

Stilbenes are natural phytochemicals that contain a 1,2-diphenylethylene (a basic skeleton of stilbenoids), and lignans are a group of diphenol derivatives with dibenzylbutane skeleton structures [15]. Due to their diverse structural makeup and established advantages for human health, lignans and stilbenes are among the most studied secondary plant metabolites [15]. Ten metabolites that fit into these classes were putatively discovered in this investigation. A total of three stilbenes (piceatannol, polydatin, and piceatannol 3-*O*-glucoside) and seven lignans were tentatively identified in these selected Australian native fruits and medicinal plants. Compound **119** (piceatannol) resulted in a deprotonated precursor ion at *m*/*z* 243 that formed a fragment ion at *m*/*z* 225 following the removal of a water unit [M−H−H_2_O], and a second product ion at *m*/*z* 201 due to the neutral loss of C_2_H_2_O (42 Da) from the precursor ion. Previously, piceatannol was found in fenugreek and dill leaves [18] and has been reported to have strong anti-mutagenic, antioxidant, anti-inflammatory, and anti-cancer properties. Compound **127** at ESI^+^ *m*/*z* 299.1279 was putatively identified in mountain pepper berries and strawberry gum, which generated product ions at *m*/*z* 281, 187, and 165 after the loss of [M−H−H_2_O], [M−H−C_6_H_8_O_2_], and [M−H−C_9_H_8_O_2_], respectively, from the precursor ion. Compound **127** was characterized as enterolactone. Enterolactone has been acknowledged for its antioxidant [58] and anti-cancer activities [59]. Compounds **122** (sesamin), **124** (silibinin), and **128** (2-hydroxyenterodiol) were only identified in mountain pepper berries.

#### 3.4.6. Other Compounds

Ten compounds were identified as other compounds. Compounds **137** (carnosic acid) and **138** (mellein) generated product ions at *m*/*z* 287 and 135, respectively, after the loss of CO_2_ (44 Da). Compound **129** at ESI^−^ *m*/*z* 125.0242 was identified in mountain pepper berries and generated product ions at *m*/*z* 107, 97, and 79 after the loss of H_2_O (18 Da) and CO (28 Da), and the removal of H_2_O after the loss of CO (18 Da). Compound **133** at ESI^−^ *m*/*z* 161.0242 was identified in mountain pepper berries and rosella, which produced fragment ion s at *m*/*z* 133, 117, and 105 through the removal of [M−H−CO], [M−H−CO_2_], and [M−H−C_2_H_2_] from the precursor ion and the former product ion, respectively. Compound **133** was tentatively characterized as umbelliferone. Compound **134** (2-hydroxybenzaldehyde) was tentatively identified only in mountain pepper berries, which produced fragment ions at *m*/*z* 92 and 77 after the loss of CO (28 Da) and CO_2_ (44 Da), respectively, from the precursor ion. Compound **132** (1,2,4,6-tetragalloyl-ꞵ-D-glucopyranose) was only identified in strawberry gum; and compounds **129** (pyrogallol), **134** (2-hydroxybenzaldehyde), and **135** (*p*-coumaraldehyde) were only identified in mountain pepper berries. A total of five limonoids were putatively detected in these native Australian fruits and spices. Compounds **139** (limonin) and **143** (citrusin) were tentatively identified only in lemon aspen.

The screening and profiling of the phenolic compounds give an overall idea of antioxidation compounds in selected Australian native plants. Strawberry gum is an excellent source of phenolic compounds, especially flavonoids used in the food, feed, cosmetics, and pharmaceutical industries because several of them have already been shown to possess high antioxidation capabilities.

### 3.5. Quantification/Semi-Quantification of Targeted Phenolic Compounds

A total of 26 compounds were quantified in Australian native mountain pepper berries, strawberry gum, rosella, and lemon aspen, which are given in Table S2. Flavonoids are the most abundant class in these selected Australian native plants. Strawberry gum was found to have the highest concentration of flavonoids, and quercitrin had the highest concentration among them (1274.04 ± 43.78 μg/g). Myricetin 3-*O*-rhamnoside (394.71 ± 16.21 μg/g), 3′,4′,5′-trimethoxyflavone (615.15 ± 21.63 μg/g), quercetin 3-*O*-arabinoside (371.54 ± 14.26 μg/g), quercetin 3-(2-galloylglucoside) (309.15 ± 20.38 μg/g), chrysin (35.52 ± 2.77 μg/g), and naringenin (24.72 ± 1.83 μg/g) were only quantified in strawberry gum. Chlorogenic acid (3-caffeoylquinic acid) is the most abundant phenolic acid in mountain pepper berries (134.05 ± 12.67 μg/g), and the lowest concentration of chlorogenic acid was quantified in strawberry gum. Previously, Konczak et al. [60] also quantified the higher concentration of chlorogenic acid in Tasmanian pepper berries. Protocatechuic acid was quantified in strawberry gum (63.56 ± 4.67 μg/g) and mountain pepper berries (44.57 ± 5.82 μg/g); *p*-hydroxybenzoic acid was quantified in rosella (11.74 ± 1.56 μg/g) and mountain pepper berries (21.91 ± 3.41 μg/g). The highest concentration of caffeic acid was found in mountain pepper berries (23.49 ± 1.92 μg/g), and the lowest concentration of caffeic acid was found in strawberry gum (15.51 ± 2.09 μg/g). Gallic acid (19.24 ± 3.12 μg/g) and *p*-coumaric acid (10.56 ± 1.35 μg/g) were found in mountain pepper berries. Gallic acid was also found in strawberry gum (23.54 ± 3.19 μg/g) and rosella (17.21 ± 2.17 μg/g). The highest concentration (39.52 ± 3.65 μg/g) of procyanidin B2 was found in strawberry gum, and the lowest concentration (11.32 ± 1.48 μg/g) was measured in lemon aspen. Rutin (56.61 ± 5.48 μg/g) was only found in mountain pepper berries. Previously, Konczak et al. [60] also found rutin in mountain pepper berries. The highest concentration of quercetin was found in mountain pepper berries (71.46 ± 4.52 μg/g), and the lowest concentration was measured in lemon aspen (18.31 ± 2.34 μg/g). The highest concentrations of isorhamnetin (26.83 ± 2.86 μg/g) and myricetin (23.67 ± 3.71 μg/g) were found in mountain pepper berries, and the lowest concentrations of isorhamnetin (12.52 ± 1.08 μg/g) and myricetin (13.16 ± 0.89 μg/g) were found in strawberry gum. A total of six anthocyanin compounds were also found in mountain pepper berries and rosella. Delphinidin 3-*O*-sambubioside (196.61 ± 17.91 μg/g) and cyanidin 3-rutinoside (142.98 ± 13.01 μg/g) were found in rosella; and delphinidin 3-*O*-sambubioside (59.67 ± 5.24 μg/g) and cyanidin 3-rutinoside (82.91 ± 7.25 μg/g) were found in mountain pepper berries. Cyanidin-3-sambubioside (72.21 ± 8.63 μg/g) and delphinidin 3-rutinoside (17.23 ± 1.61 μg/g) were only found in rosella.

**Figure 3:**
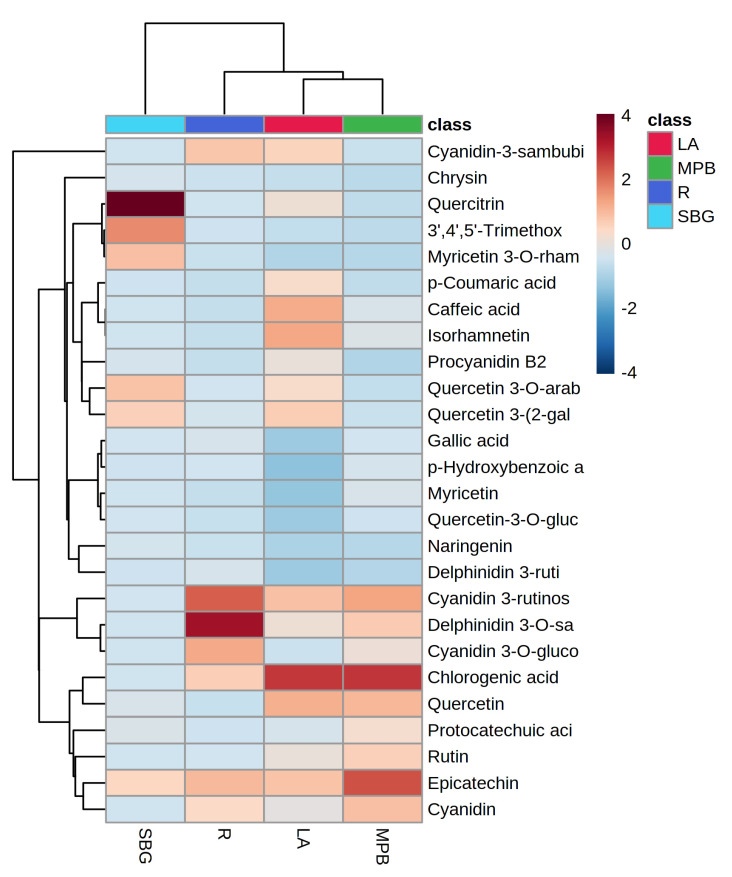
Heatmap hierarchical clustering of quantified phenolic compounds in mountain pepper berries (**MPB**), lemon aspen (**LS**), rosella (**R**), and strawberry gum (**SBG**).

Furthermore, hierarchical heatmap clustering (Figure 3) was conducted by using MetaboAnalyst 5.0 (www.metaboanalyst.ca) accessed on 7 November 2022. 

It depicted in the heatmap that quercitrin, 3′,4′,5′-trimethoxyflavone, myricetin 3-*O*-rhamnoside, quercetin 3-*O*-arabnoside, quercetin 3-(2-galloylglucoside), and epicatechin had higher concentrations than other quantified phenolic compounds in strawberry gum; and delphinidin 3-*O*-sambubioside, cyanidin 3-rutinoside, cyanidin 3-glucoside, cyanidin-3-sambubioside, cyanidin, epicatechin, and chlorogenic acid had higher concentrations in rosella. The highest concentrations of chlorogenic acid were found in mountain pepper berries and lemon aspen. Mountain pepper berries had higher concentrations of chlorogenic acid, epicatechin, cyanidin 3-rutinoside, quercetin, cyanidin, delphinidin 3-*O*-sambubiode, rutin, and protocatechuic acid.

### 3.6. Molecular Docking

**Figure 4:**
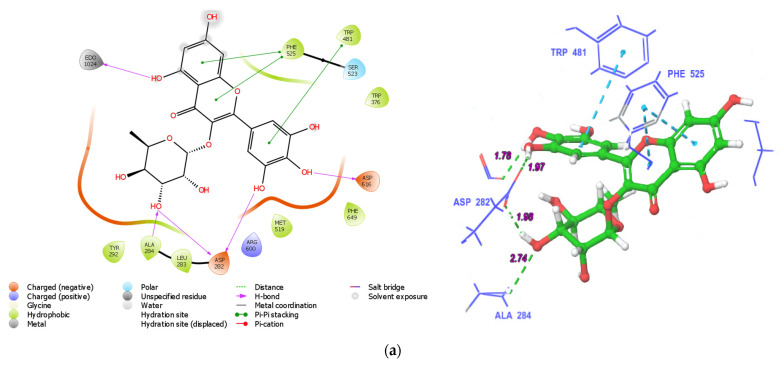

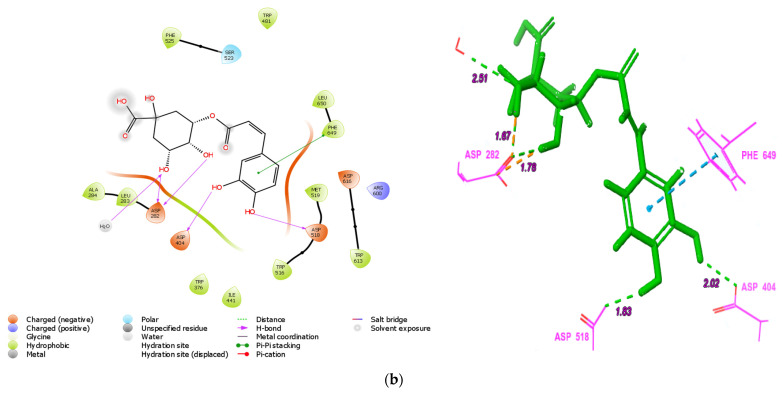
(**a**) The estimated binding geometry (2D (**left**) and 3D (**right**)) of myricitrin in 5NN8. The active side residues are named with three letters. (**b**) The estimated binding geometry (2D (**left**) and 3D (**right**)) of chlorogenic acid in 5NN8. The active side residues are named with three letters.

In silico molecular docking was conducted to predict the roles of abundant phenolic compounds in α-glucosidase inhibition activity. The estimated binding geometry 2D and 3D structures of myricitrin and chlorogenic acid in α-glucosidase protein (5NN8) are given in Figure 4A,B; and the calculated binding energy, glide energy, and binding geometry 2D of selected phenolic compounds are given in Table S3 and Figure S3.

All compounds were properly docked in 5NN8. Myricitrin (Figure 4A) and quercitrin made two hydrogen bonds with ASP 282 (negatively charged) and one each with ASP 616 (negatively charged), ALA 284 (hydrophobic), and EDO 1024. They had double Pi–Pi stacking hydrophobically with PHE 525 and a single Pi–Pi bond with TRP 481. Chlorogenic acid (Figure 4B) made two hydrogen bonds with ASP 282, one hydrogen bond each with ASP 518 and ASP 404, and one with a water molecule; and it had π–π staking with the hydrophobic PHE 649. Quercetin 3-(2-galloylglucoside) made hydrogen bonds with ASP 282 (negatively charged), ASP 616 (negatively charged), ASP 518 (negatively charged), ASP 404 (negatively charged), ALA 284 (hydrophobic), ARG 600 (positively charged), PHE 525 (hydrophobic) and water molecules; and Pi–Pi bonds with TRP 481, PHE 525, and PHE 649. Moreover, delphinidin 3-rutinoside, delphinidin 3-sambubioside, and rutin made four hydrogen bonds (two with ASP 616, one with ASP 404, and one with ASP 518), six hydrogen bonds (two ASP 282 and one each with ASP 518, ASP 403, EDO 1024, and a water molecule), and six hydrogen bonds (ASP 282, ASP 404, ASP 518, ASP 616, ASN 524 and SER 523); they also had π–π stacking in one (TRP 481), two (TRP 481 and PHE 649), and four (two with PHE 649, one with TRP 481, and one with TRP 376) bonds, respectively. Acarbose made twelve hydrogen bonds (two each with ASP 518, ASP 404, ASP 282, and SER 523; three OH groups from water molecules, which further made hydrogen bonds with ASP 645 and ARG 281). Naringin made hydrogen bonds with ASP 282, PHE 525, LEU 678, EDO 1024, and ARG 281 and one Pi–Pi stacking interaction with TRP 481. Furthermore, diosmin made three hydrogen bonds with the negatively charged ASP 282 and one with the negatively charged ASP 616 (Figure S3). The binding energies of quercetin 3-(2-galloylglucoside), delphinidin 3-rutinoside, cyanidin 3-O-rutinoside, delphinidin 3-sambubioside, rutin, acarbose, cyanidin 3-rhamnoside 5-glucoside, delphinidin, procyanidin B2, myricitrin, 3-feruloylquinic acid, taxifolin, diosmin, quercitrin, chlorogenic acid, naringin, 3-*p*-coumaroylquinic acid, myricetin, quercetin, isorhamnetin, quinic acid, luteolin, (-)-epicatechin, hesperetin, and gallic acid in 5NN8 were calculated as − 11.09, − 11.08, −10.90, −10.38, −10.14, −9.65, −9.46, −8.48, −8.05, −7.59, −7.32, −7.13, −6.84, −6.72, −6.62, −6.40, −6.35, −6.28, −5.95, −5.68, −6.65, −5.52, −5.36, −5.28, and −5.15 kcal/mol, respectively (Table S3). From the given results, it is predicted that quercetin 3-(2-galloylglucoside) identified in strawberry gum has higher α-glucosidase-inhibiting activity than acarbose. Overall, flavonoids are predicted to have higher binding affinities than the other selected phenolic compounds. Interestingly, 3-feruloylquinic acid has a higher binding affinity than taxifolin, diosmin, quercitrin, naringin, myricetin, quercetin, isorhamnetin, and luteolin chlorogenic acid; and 3-*p*-coumaroylquinic acid has a higher binding affinity than myricetin, quercetin, isorhamnetin, luteolin, (-)-epicatechin, hesperetin, diosmetin, and naringenin (Table S3). In silico molecular docking is a prediction of possible interactions between target proteins (5NN8) and potential inhibitors. Therefore, it is critical to assess the inhibitory activities of individual purified phenolic compounds to establish the precise roles of individual bioactive compounds in the inhibition of α-glucosidase. Moreover, the insights into inhibitory mechanisms of bioactive polyphenolic compounds against α-glucosidase and other proteins involved in diabetic conditions can be revealed through advanced molecular dynamics techniques and free-energy calculations, and through inverse molecular docking [61].

### 3.7. Pharmacokinetics Study of Selected Phenolic Compounds

Using computational methods to test the potential drug metabolites helps reduce the number of experimental studies and improve the success rate in pharmacokinetics studies. Absorption, distribution, metabolism, excretion, and toxicological (ADMET) screening were also conducted to validate this study for drug discovery. The interaction of inhibitors with a target receptor cannot guarantee the suitability of phenolic metabolites as drugs for the target pathology. Therefore, ADMET screening of compounds is critical in drug discovery. Unfavorable characteristics of ADMET in the biological system are the main reasons for the failure of drug molecules during clinical experiments [7]. This study evaluated the most abundant phenolic compounds identified in selected plants for ADMET properties.

#### 3.7.1. Absorption and Distribution

**Figure 5:**
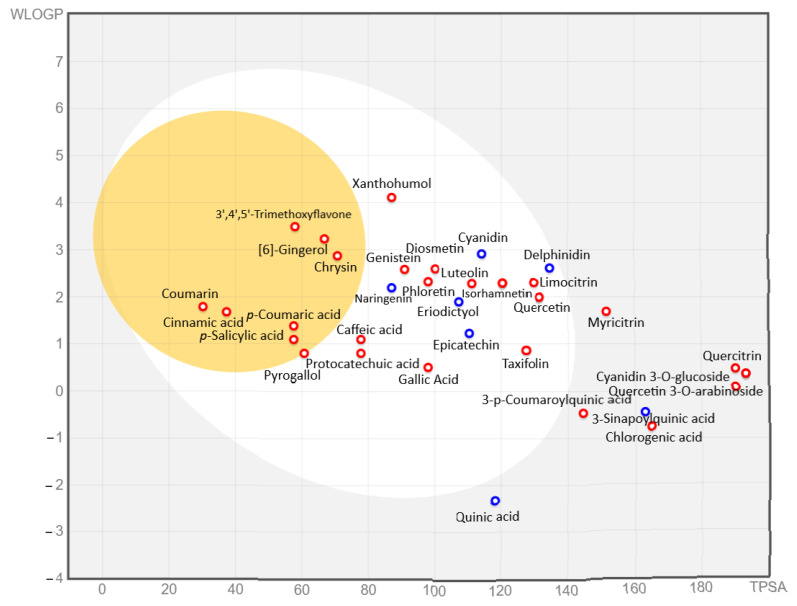
Evaluation of abundant phenolic compounds through the BOILED-Egg method. The blue dots indicate molecules predicted to be expelled from the CNS by P-glycoprotein, and the red dots indicate molecules predicted not to be expelled from the CNS by P-glycoprotein. The egg-yolk area predicts the phenolic metabolites that will passively penetrate the blood–brain barrier. In contrast, the egg-white area predicts which phenolic compounds will be absorbed through the gastrointestinal tract.

The absorption of the phenolic compounds was predicted through the BIOLED-Egg method and using the pkCSM platform. The results of absorption are given in Figure 5 and Table S4 and Table S5.

Figure 5 predicts that cinnamic acid, coumarin, *p*-coumaric acid, *p*-hydroxybenzoic acid, chrysin, [6]-gingerol, and 3′,4′,5′-trimethoxyflavone pass through the blood–brain barrier; and gallic acid, protocatechuic acid, caffeic acid, pyrogallol, cyanidin, taxifolin, epicatechin, delphinidin, naringenin, genistein, phloretin, quercetin, diosmetin, isorhamnetin, limocitrin, and eriodictyol should be absorbed through the gastrointestinal tract. Moreover, the results predict that the cinnamic acid found in mountain pepper berries and strawberry gum will more readily cross the blood–brain barrier than other phenolic compounds (Table S4). 3′,4′,5′-Trimethoxyflavone (98.1%), coumarin (97.3%), cinnamic acid (94.8%), chrysin (93.8%), *p*-coumaric acid (93.5%), genistein (93.4%), [6]-gingerol (92.4%) naringenin (91.3%), xanthohumol (89.9%), cyanidin (87.3%), *p*-hydroxybenzoic acid (84%), pyrogallol (83.6%), and luteolin (81.1%) are predicted to have the highest human intestinal absorption. Coumarin is the only compound which is predicted to pass through the skin. It is worth noting that anthocyanin aglycones with sugar moieties are predicted to have no human intestinal absorption (Table S4). Therefore, we can predict that anthocyanins with sugar moieties may play a role in gut modulation after the breakdown through colonic fermentation into their basic aglycones, or they will play a role as prebiotic polyphenols. Additionally, cinnamic (1.72), coumarin (1.65), 3′,4′,5′-trimethoxyflavone (1.39), *p*-coumaric acid (1.21), *p*-hydroxybenzoic acid (1.15), pyrogallol (1.12), naringenin (1.03), chrysin (0.95), [6]-gingerol (0.94), and taxifolin (0.92), are predicted to have the highest Caco-2 cell permeability. If the Caco2 permeability value is higher than 0.90, a compound is considered to have high Caco-2 permeability. Furthermore, the compounds which have Caco-2 permeability, gastrointestinal absorption, a good bioavailability score, and obey Lipinski’s rule of five while not being able to pass through the BBB, not acting as P-gp substrates, and having poor skin permeability should be successful drugs [62].

Most of the flavonoids that are not absorbed in the gastrointestinal tract can be metabolized by gut microbiota into small phenolic metabolites, where they tend to be absorbed in the colon [48]. Flavonoids are bound to albumin and transported to the liver through the portal vein after absorption. However, the bioavailability of flavonoids is low due to the limited absorption, extensive metabolism, and rapid excretion [63].

#### 3.7.2. Drug-Likeness

**Figure 6:**
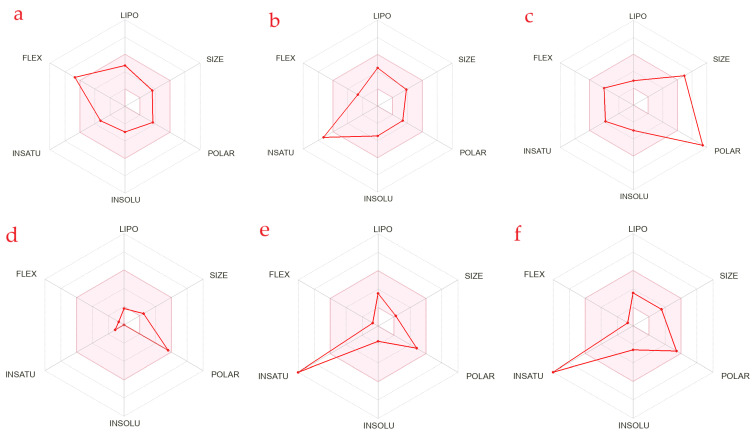
The pink area of the bioavailability radar represents the optimal range for each property. Radars of [6]-gingerol (**a**), 3′,4′,5′-trimethoxyflavone (**b**), naringin (**c**), quinic acid (**d**), gallic acid (**e**), and cyanidin (**f**) were obtained.

The bioavailability radars of selected compounds were obtained by following the method of Daina et al. [17] to predict the drug-likeness to assess the oral bioavailability of compounds (Figure 6).

Figure 6 and Table S6 depict that no compound predicted oral bioavailability except quinic acid. To predict the oral bioavailability of selected compounds, six physiochemical properties (size, polarity, lipophilicity, flexibility, saturation, and solubility) were considered and analyzed through the bioavailability radar.

#### 3.7.3. Metabolism, Excretion, and Toxicity

Cytochrome P450 (CYP) plays a vital role in the metabolism of bioactive compounds (drugs) [63]. The predicted metabolism and excretion of the phenolic compounds are given in Table S7. Metabolism was predicted through the CYP model for substrate or inhibitor (CYP1A2, CYP2D6, CYP3A4, CYP2C9, and CYP2C19). Bioactive compounds that inhibit the CYP pathway may cause elevated concentrations of other bioactive compounds, resulting in higher toxicity of that compound and vice versa. Bioactive compounds with higher total clearance are predicted to have higher bioavailability and metabolism in the liver (Table S5). Virtual toxicological screening of the bioactive compounds is provided in Table S8. The predicted results indicate that all bioactive compounds do not inhibit the hERG 1 channel, and no compound predicted AIME toxicity, hepatotoxicity, skin sensitization, *Tetrahymena pyriformis* toxicity, or Minnow toxicity except 3′,4′,5′-trimethoxyflavone, which predicted toxicity in Minnow.

## 4. Conclusions

In this study, native Australian fruits and spices were comprehensively analyzed for polyphenols, and a total of 143 metabolites were identified. Twenty-six of these compounds were quantified. Strawberry gum had higher total phenolic content, antioxidant capacity, and α-glucosidase inhibition activity than rosella, lemon aspen, and mountain pepper berries. Furthermore, in silico molecular docking predicted that flavonoids have a significant role in the inhibition of α-glucosidase. Additionally, simulated pharmacokinetics predicted that all screening phenolic compounds from native Australian fruits and spices are safe and do not have any toxicity, and small phenolic metabolites such as phenolic acids have higher absorption in Caco-2 cells and the gastrointestinal tract than other phenolic compounds. This study demonstrates that strawberry gum has a significant medicinal and pharmaceutical potential that could be utilized in food, feed, cosmetic, and pharmaceutical industries with the further proved in vivo data.

## Figures and Tables

**Table 1:** Quantification of phenolic contents in Australian native fruits and spices.

Variables	TPC mg GAE/g	TFC mg QE/g	TCT mg CE/g	TMAC mg C3GE/g
Rosella	5.65 ± 0. 48 ^b^	1.33 ± 0.10 ^c^	1.26 ± 1.13 ^d^	0.08 ± 0.02
Mountain pepper berries	6.10 ± 0.34 ^cd^	1.73 ± 0.15 ^b^	2.37 ± 0.10 ^b^	0.17 ± 0.03
Lemon aspen	4.40 ± 0.38 ^c^	0.79 ± 0.04 ^d^	1.80 ± 0.35 ^c^	0.00 ± 0.00
Strawberry gum	36.57 ± 1.34 ^a^	15.69 ± 2.69 ^a^	8.05 ± 0.52 ^a^	0.00 ± 0.00

Total phenolic content (TPC), total flavonoid content (TFC), total condensed tannins (TCT), total monomeric anthocyanin content (TMAC), cyanidin 3-glucoside equivalent (C3GE), gallic acid equivalent (GAE), quercetin equivalent (QE), catechin equivalent (CE). Values are presented as mean ± standard deviation (*n* = 3) per gram of dry weight. Values within the same column with different superscripts (^a–d^) are significantly different.

**Table 2:** Pearson correlation between phenolic contents and biological activities.

Variables	TPC	TFC	TCT	TMAC	DPPH	ABTS	FRAP	PMA	FICA	^•^OH-RSA	RPA
TFC	**1.00**										
TCT	**0.99**	**0.99**									
TMAC	−0.48	−0.47	−0.44								
DPPH	**0.98**	**0.98**	**0.95**	−0.38							
ABTS	0.62	0.63	0.66	0.38	0.66						
FRAP	**0.92**	**0.92**	0.87	−0.31	**0.98**	0.64					
PMA	−0.21	−0.20	−0.13	0.90	−0.18	0.62	−0.19				
FICA	0.88	0.88	**0.91**	−0.04	0.87	**0.91**	0.80	0.28			
^•^OH-RSA	**0.97**	**0.97**	**0.96**	−0.27	**0.99**	0.76	**0.96**	−0.04	**0.93**		
RPA	**0.99**	**0.99**	**1.00**	−0.46	**0.95**	0.65	0.86	−0.14	**0.91**	**0.95**	
* α-glu	0.79	0.80	0.84	0.11	0.79	**0.95**	0.71	0.43	**0.99**	0.87	0.83

* = α-glucosidase inhibition activity (%), values in bold are different from 0 with a significance level alpha = 0.1.

**Table 3:** LC-MS/MS characterization of phenolic metabolites in Australian native fruits and spices.

No.	Proposed Compounds	Molecular Formula	RT (min)	ESI +/−	Theoretical (*m*/*z*)	Observed (*m*/*z*)	Mass Error (ppm)	MS/MS	Samples
	**Phenolic acids**								
	**Hydroxybenzoic acids and derivatives**								
1	Gallic acid 4-*O*-glucoside	C_13_H_16_O_10_	5.212	[M−H]^−^	331.0671	331.0676	1.5	169, 125	MPB
2	Gallic acid	C_7_H_6_O_5_	6.288	[M−H]^−^	169.0142	169.0140	−1.2	125	MPB, SBG, R
3	Protocatechuic acid 4-*O*-glucoside	C_13_H_16_O_9_	8.861	[M−H]^−^	315.0721	315.0699	−7.0	153, 109	R, MPB
4	Protocatechuic acid	C_7_H_6_O_4_	9.341	[M−H]^−^	153.0193	153.0179	−9.1	109	MPB, SBG, R
5	*p*-Hydroxybenzoic acid	C_7_H_6_O_3_	12.359	[M−H]^−^	137.0244	137.0244	0.0	93	R, MPB
6	3-*O*-Methylgallic acid	C_8_H_8_O_5_	14.708	[M−H]^−^	183.0299	183.0296	−1.6	168, 124, 78	R, SBG
7	Benzoic acid	C_7_H_6_O_2_	26.507	[M−H]^−^	121.0295	121.0300	4.1	77	R, LA, SBG, MBP
8	4-Hydroxybenzoic acid 4-*O*-glucoside	C_13_H_16_O_8_	35.326	[M−H]^−^	299.0772	299.0773	0.3	255, 137	SBG, R
	**Cinnamic acids and derivatives**								
9	3-Feruloyquinic acid lactone	C_17_H_18_O_8_	3.926	[M−H]^−^	349.0929	349.0921	−2.3	193, 191, 178	SBG
10	*p*-Coumaroyl malic acid	C_13_H_12_O_7_	4.495	[M−H]^−^	279.0510	279.0503	−2.5	163	LA, SBG
11	Ferulic acid 4-*O*-glucuronide	C_16_H_18_O_10_	6.585	[M−H]^−^	369.0827	369.0828	0.3	193	R, MPB, SBG, LA
12	1-*O*-Caffeoyl-ꞵ-D-glucose	C_15_H_18_O_9_	13.291	[M−H]^−^	341.0873	341.0870	−0.9	179, 135	SBG
13	Feruloyl tartaric acid	C_14_H_14_O_9_	14.091	[M−H]^−^	325.0565	325.0562	−0.9	193, 149, 105	SBG
14	Rosmarinic acid	C_18_H_16_O_8_	14.395	[M−H]^−^	359.0772	359.0753	−5.3	197, 179, 135	MPB
15	*trans p*-Coumaric acid 4-glucoside	C_15_H_18_O_8_	15.213	[M−H]^−^	325.0929	325.0926	−0.9	163, 119	SBG, LA, MPB
16	3-Caffeoylquinic acid	C_16_H_18_O_9_	15.691	[M−H]^−^	353.0878	353.0875	−0.8	191, 179, 161, 135	R, SBG, LA, MPB
17	Caffeic acid	C_9_H_8_O_4_	16.614	[M−H]^−^	179.0350	179.0343	−3.9	135	SBG, MPB, R, LA
18	Cinnamoyl glucose	C_15_H_18_O_7_	16.808	[M−H]^−^	309.0979	309.0979	0.0	147	R
19	1-*O*-Sinapoyl-ꞵ-D-glucose	C_17_H_22_O_10_	16.945	[M−H]^−^	385.1140	385.1139	−0.3	223	LA, MPB
20	Cinnamic acid	C_9_H_8_O_2_	17.423	[M−H]^−^	147.0451	147.0458	4.8	103	SBG, MPB
21	3-*p*-Coumaroylquinic acid	C_16_H_18_O_8_	18.518	[M−H]^−^	337.0929	337.0918	−3.3	191, 119	MPB, LA, R
22	3-Feruloylquinic acid	C_17_H_20_O_9_	19.200	[M−H]^−^	367.1034	367.1029	−1.4	191	R, LA
23	3-Sinapoylquinic acid	C_18_H_22_O_10_	21.997	[M−H]^−^	397.1140	397.1141	0.3	223, 191	LA, R
24	*p*-Coumaroyl tartaric acid	C_13_H_12_O_8_	22.056	[M−H]^−^	295.0459	295.0464	1.7	115	LA
25	*p*-Coumaric acid	C_9_H_8_O_3_	22.309	[M−H]^−^	163.0400	163.0400	0.0	119	R, LA, MPB
26	1,2-Disinapoylgentiobiose	C_34_H_42_O_19_	23.851	[M−H]^−^	753.2247	753.2247	0.0	223, 207	SBG, LA
27	Sinapic acid	C_11_H_12_O_5_	24.062	[M−H]^−^	223.0612	223.0618	2.7	193, 179, 149, 134	LA
28	Feruloyl glucose	C_16_H_20_O_9_	26.100	[M−H]^−^	355.1034	355.1038	1.1	223, 207	LA, MPB
29	1,2-Diferuloylgentiobiose	C_32_H_38_O_17_	26.359	[M−H]^−^	693.2036	693.2042	0.9	193, 134	LA, MPB, SBG
30	1,5-Dicaffeoylquinic acid	C_25_H_24_O_12_	26.770	[M−H]^−^	515.1195	515.1197	0.4	191, 179, 135	R, SBG
31	*p*-Coumaroyl glycolic acid	C_11_H_10_O_5_	60.679	[M+H]^+^	223.0601	223.0605	1.8	205, 147, 119	R, MPB
	**Flavonoids**								
	**Anthocyanins**								
32	Delphinidin 3-*O*-sambubioside	C_26_H_29_O_16_	11.988	[M]^+^	597.1456	597.1471	2.5	303	R, MBP
33	Cyanidin 3-sambubioside	C_26_H_29_O_15_	13.177	[M]^+^	581.1506	581.1526	3.4	287	R
34	Cyanidin	C_15_H_11_O_6_	13.926	[M]^+^	287.0556	287.0522	−11.8	231, 139, 69	MPB, R
35	Cyanidin 3-rutinoside	C_27_H_31_O_15_	13.621	[M]^+^	595.1663	595.1660	−0.5	287	MPB
36	Cyanidin 3-*O*-glucoside	C_21_H_21_O_11_	14.461	[M]^+^	449.1084	449.0994	20.4	287	MPB, R
37	Peonidin 3-*O*-(6″-p-coumaroyl-glucoside)	C_31_H_29_O_13_	16.378	[M]^+^	609.1608	609.1617	1.5	301	MPB
38	Delphinidin 3-rutinoside	C_27_H_31_O_16_	20.460	[M]^+^	611.1612	611.1623	1.8	449, 303	R
39	Delphinidin 3-galatoside	C_21_H_21_O_12_	20.460	[M]^+^	465.1033	465.1033	0.0	303	R
40	Delphinidin 3-*O*-(6″-p-coumaroyl-glucoside)	C_30_H_27_O_14_	20.528	[M]^+^	611.1401	611.1430	4.7	303	MPB, R
41	Delphinidin	C_15_H_11_O_7_	20.528	[M]^+^	303.0505	303.0495	−3.3	303	MPB, R
	**Flavanols**								
42	Theaflavin 3-*O*-gallate	C_36_H_28_O_16_	4.172	[M+H]^+^	717.1450	717.1418	−4.5	699, 565, 139	SBG
43	Prodelphinidin trimer GC-GC-C	C_45_H_38_O_20_	6.333	[M−H]^−^	897.1883	897.1906	2.6	879, 305, 289, 125	SBG, R
44	(−)-Epigallocatechin	C_15_H_14_O_7_	12.207	[M−H]^−^	305.0667	305.0650	−5.6	289, 245, 179	R
45	4′,4″-Dimethylepigallocatechin gallate	C_24_H_22_O_11_	13.524	[M−H]^−^	485.1089	485.1092	0.6	441, 319, 183, 139	SBG
46	(−)-Epicatechin	C_15_H_14_O_6_	15.19	[M−H]^−^	289.0717	289.0711	−2.1	245, 205	SBG, R, MPB,
47	Cinnamtannin A2	C_60_H_50_O_24_	17.559	[M−H]^−^	1153.2619	1153.2602	−1.5	1135, 577, 289, 125	SBG
48	Catechin 3′-glucoside	C_21_H_24_O_11_	20.08	[M−H]^−^	451.1246	451.1253	1.6	289, 245	LA, MPB
	**Flavanones**								
49	Naringin 6′-malonate	C_30_H_34_O_17_	3.858	[M−H]^−^	665.1723	665.1701	−3.3	579	SBG
50	6″-Acetylliquiritin	C_23_H_24_O_10_	6.288	[M−H]^−^	459.1297	459.1313	3.5	441, 255	SBG, R
51	Narirutin 4′-*O*-glucoside	C_33_H_42_O_19_	20.803	[M−H]^−^	741.2247	741.2269	3.0	579, 271	LA, SBG
52	Hesperetin 5-glucoside	C_22_H_24_O_11_	23.183	[M−H]^−^	463.1246	463.1252	1.3	301	LA
53	Hesperetin 3′-*O*-glucuronide	C_22_H_22_O_12_	23.249	[M−H]^−^	477.1038	477.1052	2.9	301	SBG, LA
54	6-Geranylnaringenin	C_25_H_28_O_5_	23.288	[M−H]^−^	407.1864	407.1864	0.0	287, 271	SBG
55	Naringenin-7-*O*-glucoside	C_21_H_22_O_10_	25.833	[M−H]^−^	433.1135	433.1154	4.4	301, 271, 151, 119	MPB, SBG
56	Eriodictyol-7-*O*-glucoside	C_21_H_22_O_11_	27.312	[M−H]^−^	449.1084	449.1046	−8.5	287, 151	SBG
57	Hesperetin	C_16_H_14_O_6_	27.569	[M−H]^−^	301.0717	301.0719	0.7	265, 221, 177, 137	LA, SBG, MPB
58	Hesperetin 5′,7-*O*-diglucuronide	C_28_H_30_O_18_	37.143	[M−H]^−^	653.1359	653.1341	−2.8	301	LA
59	Eriodictyol	C_15_H_12_O_6_	37.692	[M−H]^−^	287.0556	287.0572	5.6	151, 135	SBG
60	Naringenin	C_15_H_12_O_5_	43.436	[M−H]^−^	271.0607	271.0623	5.9	227, 151, 119, 107	SBG
61	8-Prenylnaringenin	C_20_H_20_O_5_	49.532	[M−H]^−^	339.1238	339.1230	−2.4	221, 147	MPB
62	5,7-Dihydroxyflavanone	C_15_H_12_O_6_	52.05	[M−H]^−^	255.0658	255.0671	5.1	213, 151	SBG
63	Hesperidin	C_28_H_34_O_15_	53.498	[M+H]^+^	611.1971	611.1966	−0.8	303	SBG
64	3′,4′,5′-Trimethoxyflavone	C_18_H_16_O_5_	56.326	[M−H]^−^	311.0920	311.0890	−9.6	296, 267	SBG
	**Flavones and isoflavones**								
65	3′-*O*-Methylmaysin	C_28_H_30_O_14_	3.942	[M−H]^−^	589.1563	589.1571	1.4	589	R, LA
66	Tetramethylscutellarein	C_19_H_18_O_6_	5.213	[M−H]^−^	341.103	341.1030	0.0	341	R, MPB
67	Velutin	C_17_H_14_O_6_	6.265	[M−H]^−^	313.0717	313.0713	−1.3	313	SBG
68	Diosmin	C_28_H_32_O_15_	16.891	[M−H]^−^	607.1668	607.1669	0.2	301	MPB, LA
69	Azaleatin 3-arabinoside	C_21_H_20_O_11_	21.170	[M−H]^−^	447.0928	447.0903	−5.6	299, 269	MBP
70	Syringetin-3-*O*-glucoside	C_23_H_24_O_13_	24.105	[M−H]^−^	507.1144	507.1160	3.2	345	LA
71	Luteolin	C_15_H_10_O_6_	28.520	[M−H]^−^	285.0404	285.0423	6.7	267, 175, 133, 107	LA, MBP, R
72	Biochanin A 7-*O*-glucoside	C_22_H_22_O_10_	31.319	[M−H]^−^	445.1135	445.1156	4.7	283	SBG
73	Apigenin 6-C-glucoside	C_21_H_20_O_10_	32.237	[M−H]^−^	431.0983	431.0967	-3.7	271	MPB
74	Apigenin	C_15_H_10_O_5_	38.457	[M−H]^−^	269.0450	269.0467	6.3	225, 149	MPB
75	Chrysoeriol 7-*O*-glucoside	C_22_H_22_O_11_	40.140	[M−H]^−^	461.1089	461.1068	−4.6	299	MPB
76	Diosmetin	C_16_H_12_O_6_	40.251	[M−H]^−^	299.0561	299.0567	2.0	284, 265, 133	LA, MPB
77	Wogonin	C_16_H_12_O_5_	51.656	[M−H]^−^	283.0607	283.0587	−7.1	268	MPB
78	Glycitein	C_16_H_12_O_5_	52.368	[M−H]^−^	283.0607	283.0617	3.5	268	MPB
79	Chrysin	C_15_H_10_O_4_	52.451	[M−H]^−^	253.0501	253.0515	5.5	253	SBG
	**Flavonols and dihydroflavonols**								
80	6-Hydroxykaempferol 3,6-diglucoside 7-glucuronide	C_33_H_38_O_23_	14.082	[M−H]^−^	801.1726	801.1826	12.5	447, 285	MBP
81	Limocitrin	C_17_H_14_O_8_	17.162	[M−H]^−^	345.0616	345.0604	−3.5	330, 315, 301, 181	LA
82	Myricetin 3-*O*-glucoside	C_21_H_20_O_13_	19.041	[M−H]^−^	479.0831	479.0816	−3.1	317	R
83	Quercetin 3-(2-galloylglucoside)	C_28_H_24_O_16_	20.371	[M−H]^−^	615.0986	615.0936	−8.1	301, 169	SBG
84	* Rutin	C_27_H_30_O_16_	20.530	[M−H]^−^	609.1461	609.1443	−3.0	301, 300, 271, 255	MPB, R
85	Myricetin 3-*O*-rhamnoside (myricitrin)	C_21_H_20_O_12_	21.328	[M−H]^−^	463.0882	463.0849	−7.1	317	SBG, R
86	Kaempferol 3-rutinoside	C_27_H_30_O_15_	24.911	[M−H]^−^	593.1507	593.1511	0.7	285, 151	MPB
87	Kaempferol 3-*O*-arabinoside	C_20_H_18_O_10_	25.902	[M−H]^−^	417.0822	417.0793	−7.0	285	MPB
88	Quercetin 3-rhamnoside (quercitrin)	C_21_H_20_O_11_	26.142	[M−H]^−^	447.0928	447.0941	2.9	301	SBG
89	Dihydroquercetin 3-*O*-rhamnoside	C_21_H_22_O_11_	26.305	[M−H]^−^	449.1089	449.1095	1.3	303	SBG, MPB
90	Quercetin 3-*O*-arabinoside	C_20_H_18_O_11_	25.269	[M−H]^−^	433.0776	433.0769	−1.6	301, 271, 151	MPB
91	Isorhamnetin 3-*O*-glucoside 7-*O*-rhamnoside	C_28_H_32_O_16_	26.574	[M−H]^−^	623.1617	623.1607	−1.6	315	R, LA
92	* Quercetin-3-*O*-glucoside	C_21_H_20_O_12_	27.258	[M−H]^−^	463.0882	463.0842	−8.6	301, 271, 255, 151	SBG, MBP, R
93	* Isorhamnetin	C_16_H_12_O_7_	29.384	[M−H]^−^	315.0510	315.0491	−6.0	300, 271, 151	LA, MPB, SBG
94	Kaempferol 3-*O*-glucoside	C_21_H_20_O_11_	30.214	[M−H]^−^	447.0928	447.0927	−0.2	285, 255, 147	MPB, R, LA
95	* Myricetin	C_15_H_10_O_8_	30.613	[M−H]^−^	317.0298	317.0314	3.3	179, 151	SBG, MPB, R
96	* Taxifolin	C_15_H_12_O_7_	31.176	[M−H]^−^	303.0510	303.0505	−1.7	217, 125	LA, MPB
97	Isorhamnetin 3-*O*-glucuronide	C_22_H_20_O_13_	31.344	[M−H]^−^	491.0831	491.0819	−2.4	315	R
98	* Quercetin	C_15_H_10_O_7_	39.148	[M−H]^−^	301.0353	301.0352	−0.3	271, 179, 151, 121	SBG, LA, MPB, R
	**Chalcones**								
99	Xanthohumol	C_21_H_22_O_5_	10.842	[M−H]^−^	353.1389	353.1399	2.8	295, 233	SBG
100	Phloretin	C_15_H_14_O_5_	28.333	[M−H]^−^	273.0768	273.0780	4.3	167, 119	SBG
101	Phloretin-2′-O-glucoside	C_21_H_24_O_10_	28.33	[M−H]^−^	435.1296	435.1303	1.5	273, 167	SBG
	**Isoflavonoids**								
102	Dihydroformononetin	C_16_H_14_O_4_	3.991	[M−H]^−^	269.0819	269.0816	−1.1	253, 239, 223	SBG, MPB
103	Equol 7-*O*-glucuronide	C_21_H_22_O_9_	6.310	[M−H]^−^	417.1191	417.1201	2.4	241	SBG
104	6″-*O*-Malonyldaidzin	C_24_H_22_O_12_	14.474	[M−H]^−^	501.1038	501.1027	−2.2	415	MPB, LA
105	6″-*O*-Acetyldaidzin	C_23_H_22_O_10_	16.980	[M−H]^−^	457.1140	457.1138	−0.4	415	SBG, R
106	Daidzin 4′-*O*-glucuronide	C_27_H_28_O_15_	21.029	[M−H]^−^	591.1355	591.1353	−0.3	415, 253	LA, R, SBG
107	3′,4′,7-Trihydroxyisoflavanone	C_15_H_12_O_5_	27.308	[M−H]^−^	271.0612	271.0611	−0.4	239, 135, 121	SBG
108	3′-*O*-Methylviolanone	C_18_H_18_O_6_	27.762	[M−H]^−^	329.1030	329.1029	−0.3	285, 163	MPB, LA
109	Daidzein 7-*O*-glucuronide	C_21_H_18_O_10_	33.742	[M−H]^−^	429.0827	429.0807	−4.7	253	R
110	3′-Hydroxymelanettin	C_16_H_12_O_6_	40.251	[M−H]^−^	299.0561	299.0567	2.0	284	LA, MPB
111	2′-Hydroxyformononetin	C_16_H_12_O_5_	52.124	[M−H]^−^	283.0612	283.0606	−2.1	268	SBG, MPB
	**Tannins**								
112	Gallagic acid	C_28_H_12_O_16_	3.075	[M−H]^−^	603.0052	603.0041	−1.8	587, 559, 549, 446	R, MPB
113	2-*O*-Galloylpunicalin	C_41_H_26_O_26_	6.333	[M−H]^−^	933.0639	933.0645	0.6	781, 169, 125	SBG
114	Glucosyringic acid	C_15_H_20_O_10_	7.546	[M−H]^−^	359.0978	359.0914	−17.8	315, 197, 153, 125	MPB
115	Procyanidin trimer C1	C_45_H_38_O_18_	16.230	[M−H]^−^	865.1985	865.2012	3.1	739, 713, 695	SBG, R
116	Procyanidin B2	C_30_H_26_O_12_	19.039	[M−H]^−^	577.1351	577.1323	−4.9	451, 425, 289, 245	MPB, LA, SBG, R
117	Punicafolin	C_41_H_30_O_26_	19.102	[M−H]^−^	937.0952	937.0966	1.5	169, 125	SBG
118	Ellagic acid	C_14_H_6_O_8_	55.906	[M−H]^−^	300.9990	300.9988	−0.7	284, 257	LA, MBP, R
	**Stilbenes**								
119	Piceatannol	C_14_H_12_O_4_	5.594	[M−H]^−^	243.0663	243.0653	−4.1	225, 201	SBG, MPB
120	Polydatin	C_20_H_22_O_8_	21.854	[M−H]^−^	389.1242	389.1245	0.8	227	LA, MPB, SBG
121	Piceatannol 3-*O*-glucoside	C_20_H_22_O_9_	30.064	[M−H]^−^	405.1191	405.1188	−0.7	243	MPB
	**Lignans**								
122	Sesamin	C_20_H_18_O_6_	4.879	[M−H]^−^	353.1030	353.1015	−4.2	338, 163	MPB
123	2-Hydroxyenterolactone	C_18_H_18_O_5_	6.371	[M−H]^−^	313.1081	313.1091	3.2	255, 163	LA, MPB
124	Silibinin	C_25_H_22_O_10_	16.794	[M−H]^−^	481.1140	481.1151	2.3	301, 179, 165, 151	MPB
125	7-Oxomatairesinol	C_20_H_20_O_7_	21.997	[M−H]^−^	371.1136	371.1138	0.5	358, 343, 328	LA
126	Arctigenin	C_21_H_24_O_6_	23.288	[M−H]^−^	371.1500	371.1497	−0.8	356, 312, 295	SBG
127	Enterolactone	C_18_H_18_O_4_	47.739	[M−H]^−^	299.1288	299.1299	4.3	281, 187, 165	MPB, SBG
128	2-Hydroxyenterodiol	C_18_H_22_O_5_	53.013	[M−H]^−^	317.1394	317.1395	0.3	299, 287, 269, 257	MPB
	**Other compounds**								
129	Pyrogallol	C_6_H_6_O_3_	7.009	[M−H]^−^	125.0244	125.0242	−1.6	107, 97, 79	MPB
130	[6]-Gingerol	C_17_H_26_O_4_	12.249	[M−H]^−^	293.1758	293.1768	3.4	137	SBG, MPB
131	Quinic acid	C_7_H_12_O_6_	4.189	[M−H]^−^	191.0561	191.0578	9.0	85	MPB, SBG
132	1,2,4,6-Tetragalloyl-ꞵ-D-glucopyranose	C_34_H_28_O_22_	19.144	[M−H]^−^	787.0999	787.0953	−5.9	169, 125	SBG
133	Umbelliferone	C_9_H_6_O_3_	19.396	[M−H]^−^	161.0244	161.0246	1.2	133	MPB, R
134	2-Hydroxybenzaldehyde	C_7_H_6_O_2_	20.620	[M−H]^−^	121.0269	121.0276	5.8	92, 77	MPB
135	*p*-Coumaraldehyde	C_9_H_8_O_2_	29.139	[M−H]^−^	147.0451	147.0463	8.0	119	MPB
136	Xanthotoxol	C_11_H_6_O_4_	47.927	[M−H]^−^	201.0193	201.0191	−1.0	171	MPB, LA, SBG
137	Carnosic acid	C_20_H_28_O_4_	55.899	[M−H]^−^	331.1915	331.1927	3.6	287	SBG, R
138	Mellein	C_10_H_10_O_3_	62.141	[M+H]^+^	179.0703	179.0694	−5.0	135	LA
	**Limonoids**								
139	Limonin	C_26_H_30_O_8_	19.039	[M−H]^−^	469.1868	469.1859	−1.9	229	LA
140	Obacunoic acid	C_26_H_32_O_8_	25.201	[M−H]^−^	471.2024	471.2027	0.6	471	LA, MPB, SBG
141	Nomilin	C_28_H_34_O_9_	51.280	[M+H]^+^	515.2276	515.2280	0.8	515	MPB, SBG
142	Obacunone	C_26_H_30_O_7_	19.253	[M−H]^−^	455.2065	455.2065	0.0	407, 163	SBG, R, MPB, LA
143	Citrusin	C_28_H_34_O_11_	55.330	[M+H]^+^	547.2174	547.2162	−2.2	547	LA

Mountain pepper berries (MPB), rosella (R), lemon aspen (LA), and strawberry gum (SBG). Compounds with asterisk (*) were identified with pure standards.

## Data Availability

The supporting data are available in the Supplementary Materials.

## Supplementary Materials

The following supporting information can be downloaded at: https://www.mdpi.com/article/10.3390/antiox12020254/s1. Figure S1: Base peak chromatograms (BPC) of mountain pepper berries, rosella, lemon aspen, and strawberry gum in positive (black) and negative (blue) modes of ionization. Figure S2: MS/MS spectra of some selected compounds. Figure S3: Two-dimensional binding geometry of some selected compounds. Table S1: Antioxidant activities of native Australian fruits and spices. Table S2: Quantification/semi-quantification of phenolic metabolites in Australian native fruits and spices (μg/g). Table S3: The calculated binding energies of selected compounds. Table S4: Predicted absorption and distribution of selected compounds. Table S5: Pharmacokinetic properties of selected compounds. Table S6: Radar bioavailability properties of selected compounds. Table S7: Metabolism and excretion of selected compounds. Table S8: Predicted toxicity of abundant phenolic compounds.

## References

[1] Ali A., Cottrell J.J., Dunshea F.R. (2022). Identification and characterization of anthocyanins and non-anthocyanin phenolics from australian native fruits and their antioxidant, antidiabetic, and anti-alzheimer potential. Food Res. Int..

[2] Ali A., Zahid H.F., Cottrell J.J., Dunshea F.R. (2022). A comparative study for nutritional and phytochemical profiling of coffea arabica (c. Arabica) from different origins and their antioxidant potential and molecular docking. Molecules.

[3] Tsao R. (2010). Chemistry and biochemistry of dietary polyphenols. Nutrients.

[4] Kiloni S.M., Akhtar A., Cáceres-Vélez P.R., Dunshea F., Jusuf P. (2022). P06-05 zebrafish embryo acute toxicity and antioxidant characterization of native australian plants: Towards safe and effective glaucoma treatments. Toxicol. Lett..

[5] Cock I.E. (2011). Medicinal and aromatic plants–australia. Ethnopharmacol. Encycl. Life Support Syst. EOLSS.

[6] Richmond R., Bowyer M., Vuong Q. (2019). Australian native fruits: Potential uses as functional food ingredients. J. Funct. Foods.

[7] Attique S.A., Hassan M., Usman M., Atif R.M., Mahboob S., Al-Ghanim K.A., Bilal M., Nawaz M.Z. (2019). A molecular docking approach to evaluate the pharmacological properties of natural and synthetic treatment candidates for use against hypertension. Int. J. Environ. Res. Public Health.

[8] Bahun M., Jukić M., Oblak D., Kranjc L., Bajc G., Butala M., Bozovičar K., Bratkovič T., Podlipnik Č., Poklar Ulrih N. (2022). Inhibition of the sars-cov-2 3cl(pro) main protease by plant polyphenols. Food Chem..

[9] Jukič M., Janežič D., Bren U. (2021). Potential novel thioether-amide or guanidine-linker class of sars-cov-2 virus rna-dependent rna polymerase inhibitors identified by high-throughput virtual screening coupled to free-energy calculations. Int. J. Mol. Sci..

[10] Ali A., Wu H., Ponnampalam E.N., Cottrell J.J., Dunshea F.R., Suleria H.A.R. (2021). Comprehensive profiling of most widely used spices for their phenolic compounds through lc-esi-qtof-ms2 and their antioxidant potential. Antioxidants.

[11] Sharifi-Rad J., Song S., Ali A., Subbiah V., Taheri Y., Suleria H.A.R. (2021). Lc-esi-qtof-ms/ms characterization of phenolic compounds from pyracantha coccinea m. Roem. And their antioxidant capacity. Cell. Mol. Biol..

[12] Chou O., Ali A., Subbiah V., Barrow C.J., Dunshea F.R., Suleria H.A.R. (2021). Lc-esi-qtof-ms/ms characterisation of phenolics in herbal tea infusion and their antioxidant potential. Fermentation.

[13] Zahid H.F., Ali A., Ranadheera C.S., Fang Z., Dunshea F.R., Ajlouni S. (2022). In vitro bioaccessibility of phenolic compounds and alpha-glucosidase inhibition activity in yoghurts enriched with mango peel powder. Food Biosci..

[14] Bashmil Y.M., Ali A., BK A., Dunshea F.R., Suleria H.A.R. (2021). Screening and characterization of phenolic compounds from australian grown bananas and their antioxidant capacity. Antioxidants.

[15] Ali A., Cottrell J.J., Dunshea F.R. (2022). Lc-ms/ms characterization of phenolic metabolites and their antioxidant activities from australian native plants. Metabolites.

[16] Ali A., Kiloni S.M., Cáceres-Vélez P.R., Jusuf P.R., Cottrell J.J., Dunshea F.R. (2022). Phytochemicals, antioxidant activities, and toxicological screening of native australian fruits using zebrafish embryonic model. Foods.

[17] Daina A., Michielin O., Zoete V. (2017). Swissadme: A free web tool to evaluate pharmacokinetics, drug-likeness and medicinal chemistry friendliness of small molecules. Sci. Rep..

[18] Ali A., Bashmil Y.M., Cottrell J.J., Suleria H.A.R., Dunshea F.R. (2021). Lc-ms/ms-qtof screening and identification of phenolic compounds from australian grown herbs and their antioxidant potential. Antioxidants.

[19] Konczak I., Zabaras D., Dunstan M., Aguas P. (2010). Antioxidant capacity and phenolic compounds in commercially grown native australian herbs and spices. Food Chem..

[20] Cáceres-Vélez P.R., Ali A., Fournier-Level A., Dunshea F.R., Jusuf P.R. (2022). Phytochemical and safety evaluations of finger lime, mountain pepper, and tamarind in zebrafish embryos. Antioxidants.

[21] Vélez P.R.C., Ali A., Fournier-Level A., Dunshea F., Jusuf P.R. (2022). P06-04 antioxidant activity and embryotoxicity of citrus australasica, tasmannia lanceolata and diploglottis australis extracts in zebrafish. Toxicol. Lett..

[22] Hu T., Subbiah V., Wu H., Bk A., Rauf A., Alhumaydhi F.A., Suleria H.A.R. (2021). Determination and characterization of phenolic compounds from australia-grown sweet cherries (prunus avium l.) and their potential antioxidant properties. ACS Omega.

[23] Lukmanto S., Roesdiyono N., Ju Y.-H., Indraswati N., Soetaredjo F.E., Ismadji S. (2013). Supercritical co2 extraction of phenolic compounds in roselle (hibiscus sabdariffa l.). Chem. Eng. Commun..

[24] Liu H., Qiu N., Ding H., Yao R. (2008). Polyphenols contents and antioxidant capacity of 68 chinese herbals suitable for medical or food uses. Food Res. Int..

[25] Yang W.-J., Li D.-P., Li J.-K., Li M.-H., Chen Y.-L., Zhang P.-Z. (2009). Synergistic antioxidant activities of eight traditional chinese herb pairs. Biol. Pharm. Bull..

[26] Yoo K.M., Lee C.H., Lee H., Moon B., Lee C.Y. (2008). Relative antioxidant and cytoprotective activities of common herbs. Food Chem..

[27] Tsai M.-L., Lin C.-C., Lin W.-C., Yang C.-H. (2011). Antimicrobial, antioxidant, and anti-inflammatory activities of essential oils from five selected herbs. Biosci. Biotechnol. Biochem..

[28] Lin C.-C., Yang C.-H., Wu P.-S., Kwan C.-C., Chen Y.-S. (2011). Antimicrobial, anti-tyrosinase and antioxidant activities of aqueous aromatic extracts from forty-eight selected herbs. J. Med. Plants Res..

[29] Garg D., Muley A., Khare N., Marar T. (2012). Comparative analysis of phytochemical profile and antioxidant activity of some indian culinary herbs. Res. J. Pharm. Biol. Chem. Sci..

[30] Chen I.-C., Chang H.-C., Yang H.-W., Chen G.-L. (2004). Evaluation of total antioxidant activity of several popular vegetables and chinese herbs: A fast approach with abts/h2o2/hrp system in microplates. J. Food Drug Anal..

[31] Köksal E., Bursal E., Gülçin İ., Korkmaz M., Çağlayan C., Gören A.C., Alwasel S.H. (2017). Antioxidant activity and polyphenol content of turkish thyme (thymus vulgaris) monitored by liquid chromatography and tandem mass spectrometry. Int. J. Food Prop..

[32] Wojdyło A., Oszmiański J., Czemerys R. (2007). Antioxidant activity and phenolic compounds in 32 selected herbs. Food Chem..

[33] Gülçin İ. (2006). Antioxidant activity of caffeic acid (3, 4-dihydroxycinnamic acid). Toxicology.

[34] Gülçin İ., Huyut Z., Elmastaş M., Aboul-Enein H.Y. (2010). Radical scavenging and antioxidant activity of tannic acid. Arab. J. Chem..

[35] Gülçın İ., Oktay M., Kıreçcı E., Küfrevıoǧlu Ö.İ. (2003). Screening of antioxidant and antimicrobial activities of anise (pimpinella anisum l.) seed extracts. Food Chem..

[36] Syabana M.A., Yuliana N.D., Batubara I., Fardiaz D. (2020). Antidiabetic activity screening and nmr profile of vegetable and spices commonly consumed in indonesia. Food Sci. Technol..

[37] Taslimi P., Köksal E., Gören A.C., Bursal E., Aras A., Kılıç Ö., Alwasel S., Gülçin İ. (2020). Anti-alzheimer, antidiabetic and antioxidant potential of satureja cuneifolia and analysis of its phenolic contents by lc-ms/ms. Arab. J. Chem..

[38] Cottrell J.J., Le H.H., Artaiz O., Iqbal Y., Suleria H.A., Ali A., Celi P., Dunshea F.R. (2022). Recent advances in the use of phytochemicals to manage gastrointestinal oxidative stress in poultry and pigs. Anim. Prod. Sci..

[39] Granato D., Shahidi F., Wrolstad R., Kilmartin P., Melton L.D., Hidalgo F.J., Miyashita K., van Camp J., Alasalvar C., Ismail A.B. (2018). Antioxidant activity, total phenolics and flavonoids contents: Should we ban in vitro screening methods?. Food Chem..

[40] Freeman B.L., Eggett D.L., Parker T.L. (2010). Synergistic and antagonistic interactions of phenolic compounds found in navel oranges. J. Food Sci..

[41] Shan B., Cai Y.Z., Sun M., Corke H. (2005). Antioxidant capacity of 26 spice extracts and characterization of their phenolic constituents. J. Agric. Food Chem..

[42] Mandal S.M., Chakraborty D., Dey S. (2010). Phenolic acids act as signaling molecules in plant-microbe symbioses. Plant Signal. Behav..

[43] Goleniowski M., Bonfill M., Cusido R., Palazón J. (2013). Phenolic acids. Nat. Prod..

[44] Kakkar S., Bais S. (2014). A review on protocatechuic acid and its pharmacological potential. ISRN Pharmacol..

[45] Hossain M.B., Rai D.K., Brunton N.P., Martin-Diana A.B., Barry-Ryan C. (2010). Characterization of phenolic composition in lamiaceae spices by lc-esi-ms/ms. J. Agric. Food Chem..

[46] Dong J., Zhu Y., Gao X., Chang Y., Wang M., Zhang P. (2013). Qualitative and quantitative analysis of the major constituents in chinese medicinal preparation dan-lou tablet by ultra high performance liquid chromatography/diode-array detector/quadrupole time-of-flight tandem mass spectrometry. J. Pharm. Biomed. Anal..

[47] Kadam D., Palamthodi S., Lele S.S. (2018). Lc-esi-q-tof-ms/ms profiling and antioxidant activity of phenolics from l. Sativum seedcake. J. Food Sci. Technol..

[48] Aherne S.A., O’Brien N.M. (2002). Dietary flavonols: Chemistry, food content, and metabolism. Nutrition.

[49] Oh Y.S., Lee J.H., Yoon S.H., Oh C.H., Choi D.S., Choe E., Jung M.Y. (2008). Characterization and quantification of anthocyanins in grape juices obtained from the grapes cultivated in korea by hplc/dad, hplc/ms, and hplc/ms/ms. J. Food Sci..

[50] Liu Y., Tikunov Y., Schouten R.E., Marcelis L.F.M., Visser R.G.F., Bovy A. (2018). Anthocyanin biosynthesis and degradation mechanisms in solanaceous vegetables: A review. Front. Chem..

[51] Murkovic M., Caballero B., Finglas P.M., Toldrá F. (2016). Phenolic compounds: Occurrence, classes, and analysis. Encyclopedia of Food and Health.

[52] Rockenbach I.I., Jungfer E., Ritter C., Santiago-Schübel B., Thiele B., Fett R., Galensa R. (2012). Characterization of flavan-3-ols in seeds of grape pomace by ce, hplc-dad-msn and lc-esi-fticr-ms. Food Res. Int..

[53] Zahid H.F., Ali A., Ranadheera C.S., Fang Z., Ajlouni S. (2023). Identification of phenolics profile in freeze-dried apple peel and their bioactivities during in vitro digestion and colonic fermentation. Int. J. Mol. Sci..

[54] Thuan N.H., Pandey R.P., Thuy T.T., Park J.W., Sohng J.K. (2013). Improvement of regio-specific production of myricetin-3-o-α-l-rhamnoside in engineered escherichia coli. Appl. Biochem. Biotechnol..

[55] Habtemariam S. (2011). A-glucosidase inhibitory activity of kaempferol-3-o-rutinoside. Nat. Prod. Commun..

[56] Chang Z., Zhang Q., Liang W., Zhou K., Jian P., She G., Zhang L. (2019). A comprehensive review of the structure elucidation of tannins from terminalia linn. Evid. Based Complement. Altern. Med..

[57] Hou K., Wang Z. (2021). Application of nanotechnology to enhance adsorption and bioavailability of procyanidins: A review. Food Rev. Int..

[58] Kitts D.D., Yuan Y.V., Wijewickreme A.N., Thompson L.U. (1999). Antioxidant activity of the flaxseed lignan secoisolariciresinol diglycoside and its mammalian lignan metabolites enterodiol and enterolactone. Mol. Cell. Biochem..

[59] Liu Z., Fei Y.J., Cao X.H., Xu D., Tang W.J., Yang K., Xu W.X., Tang J.H. (2021). Lignans intake and enterolactone concentration and prognosis of breast cancer: A systematic review and meta-analysis. J. Cancer.

[60] Konczak I., Zabaras D., Dunstan M., Aguas P. (2010). Antioxidant capacity and hydrophilic phytochemicals in commercially grown native australian fruits. Food Chem..

[61] Pantiora P., Furlan V., Matiadis D., Mavroidi B., Perperopoulou F., Papageorgiou A.C., Sagnou M., Bren U., Pelecanou M., Labrou N.E. (2023). Monocarbonyl curcumin analogues as potent inhibitors against human glutathione transferase p1-1. Antioxidants.

[62] Khalfaoui A., Noumi E., Belaabed S., Aouadi K., Lamjed B., Adnan M., Defant A., Kadri A., Snoussi M., Khan M.A. (2021). Lc-esi/ms-phytochemical profiling with antioxidant, antibacterial, antifungal, antiviral and in silico pharmacological properties of algerian asphodelus tenuifolius (cav.) organic extracts. Antioxidants.

[63] Khan J., Deb P.K., Priya S., Medina K.D., Devi R., Walode S.G., Rudrapal M. (2021). Dietary flavonoids: Cardioprotective potential with antioxidant effects and their pharmacokinetic, toxicological and therapeutic concerns. Molecules.

