# Supplementary material for: Antioxidant, Alpha-Glucosidase Inhibition Activities, In Silico Molecular Docking and Pharmacokinetics Study of Phenolic Compounds from Native Australian Fruits and Spices
Source: Antioxidants (Basel). 2023 Jan 23;12(2):254. doi: 10.3390/antiox12020254 (PMC9952698; doi:10.3390/antiox12020254)
Supplement: Supplementary file 1 [file antioxidants-12-00254-s001.zip › antioxidants-2146639-supplementary.pdf]

Supplementary Material

Antioxidant, Alpha-Glucosidase Inhibition Activities, *In silico* Molecular Docking and Pharmacokinetics Study of Phenolic Compounds from Native Australian Fruits and Spices

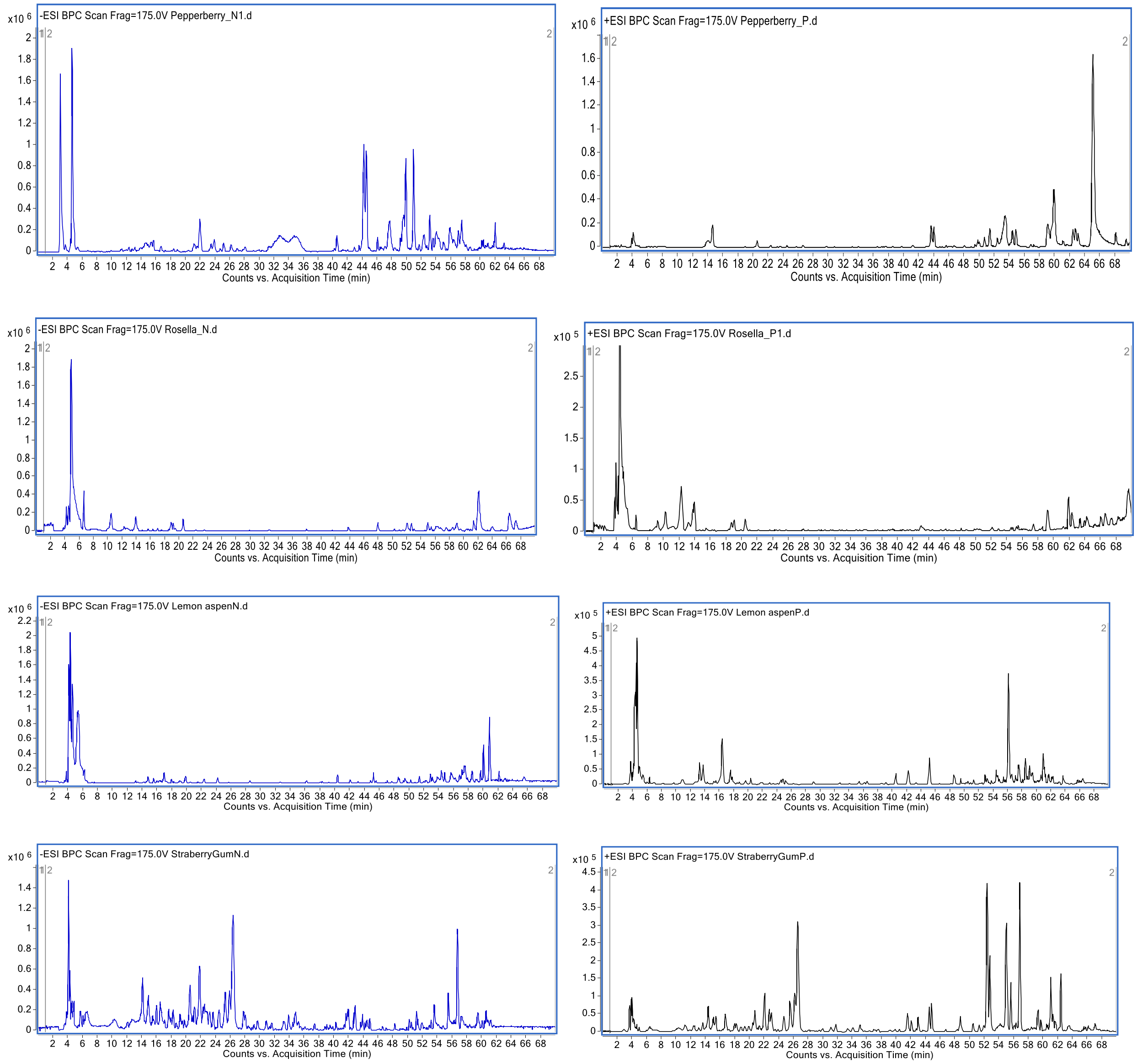

Figure S1. Base peak chromatograms (BPC) of mountain pepper berries, rosella, lemon aspen, and strawberry gum in positive (black) and negative (blue) modes of ionization.

p-Hydroxybenzoic acid

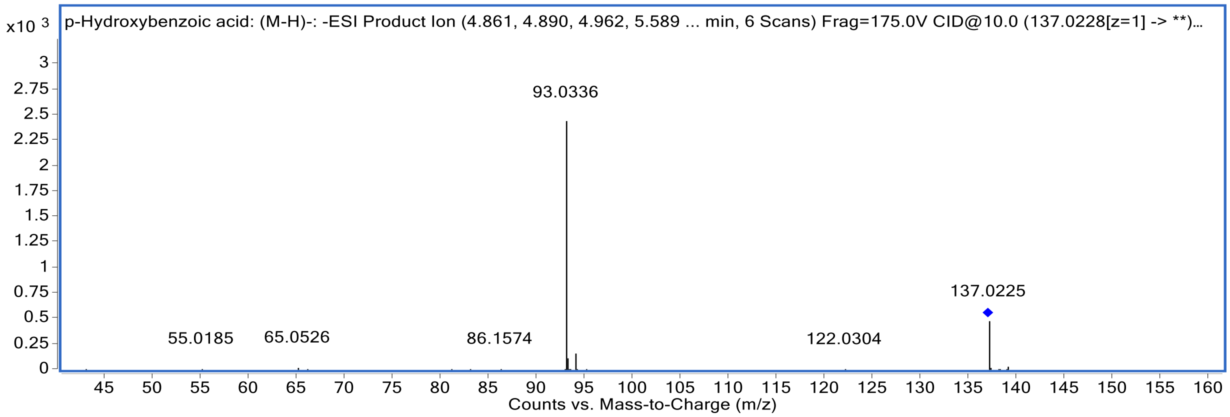

Protocatechuic acid

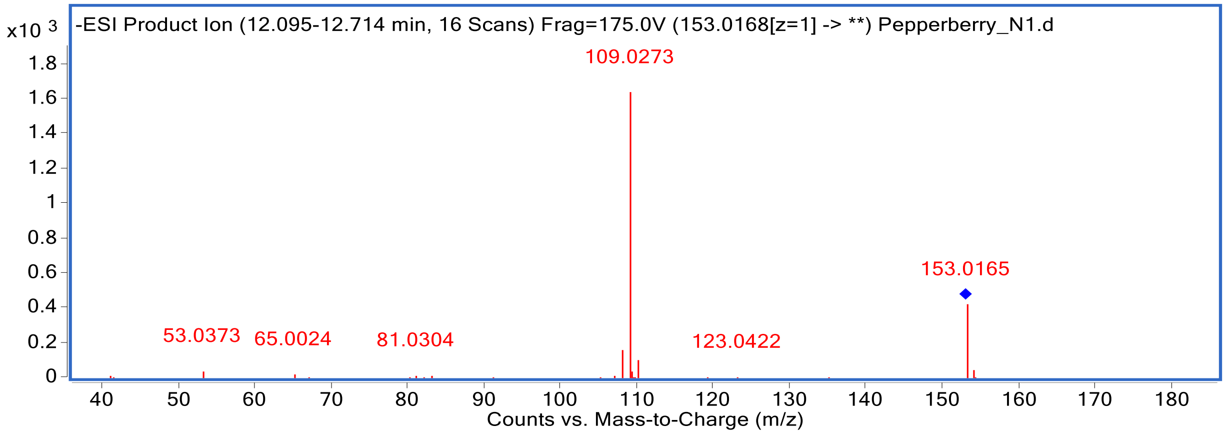

Luteolin

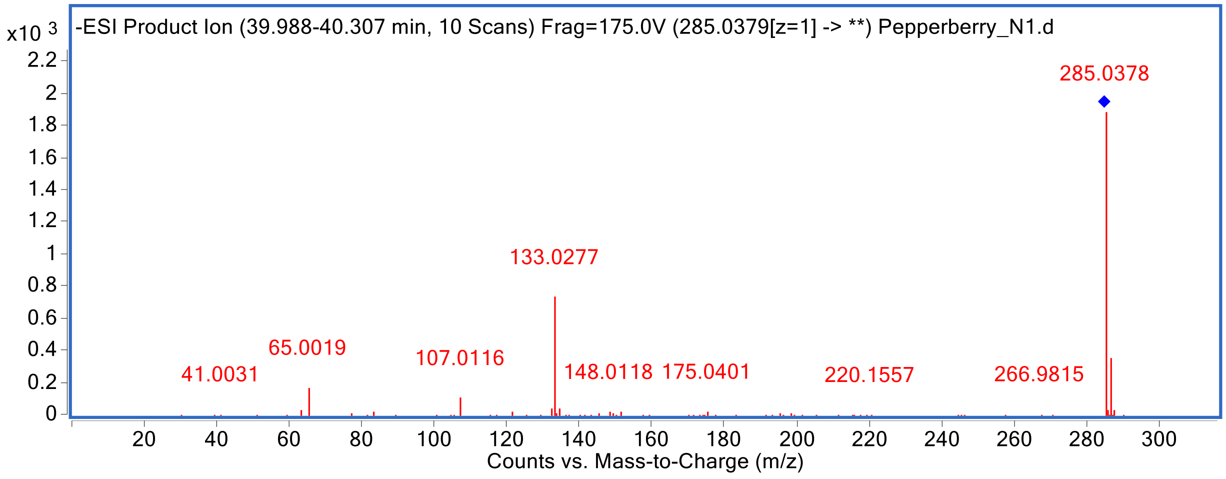

Epicatechin

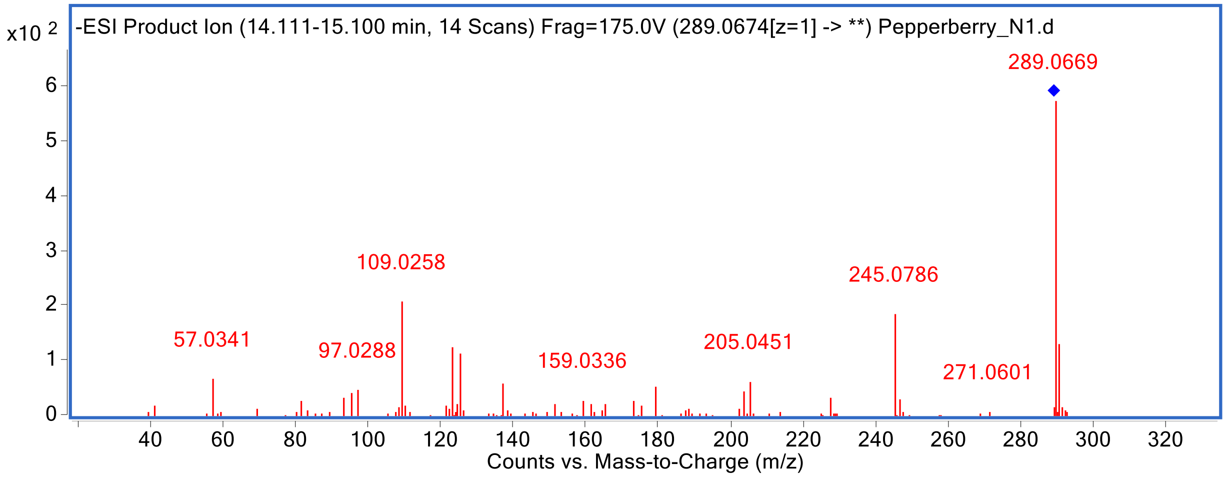

Diosmetin

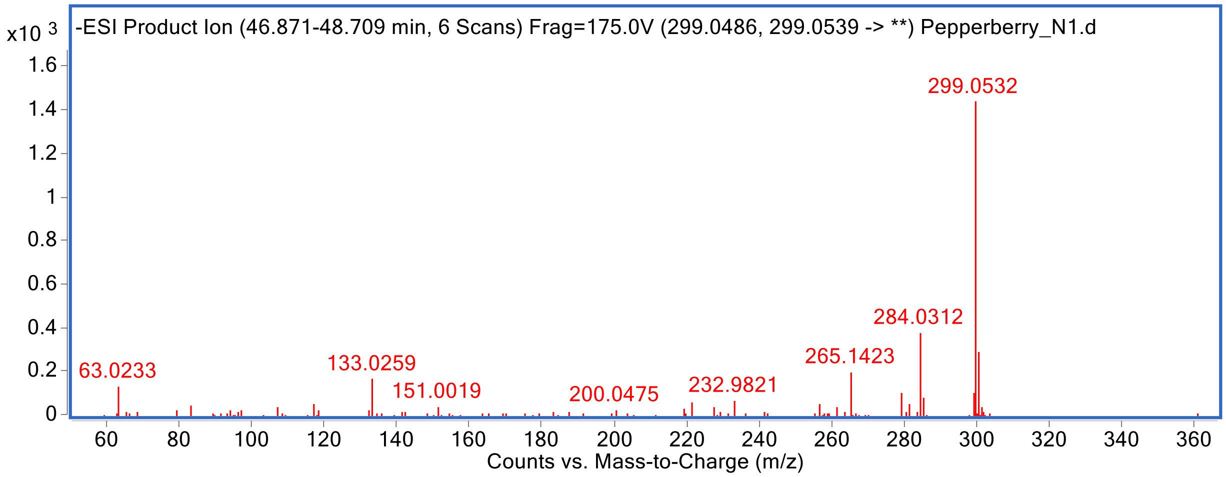

Quercetin

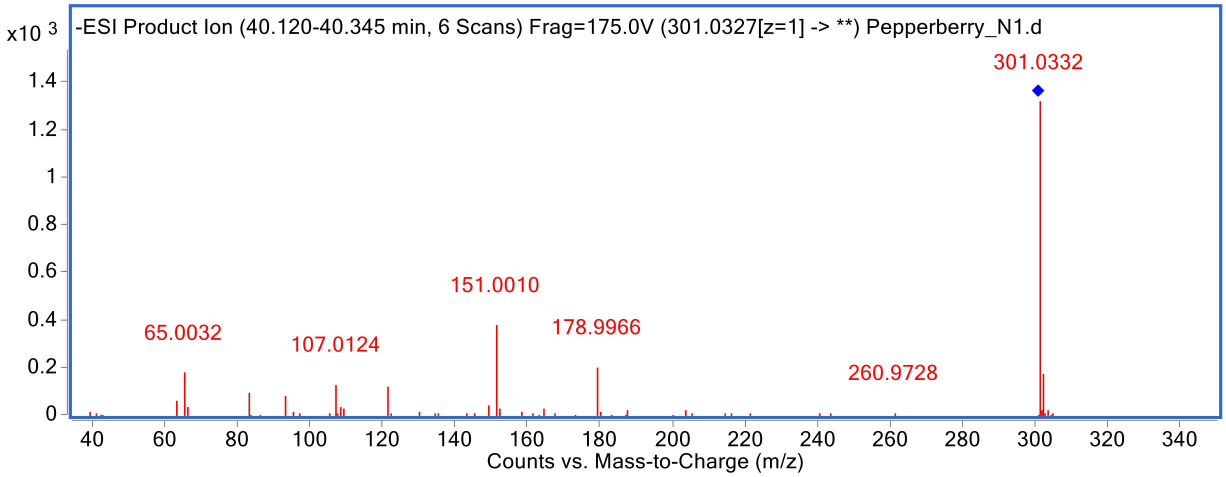

Hesperetin

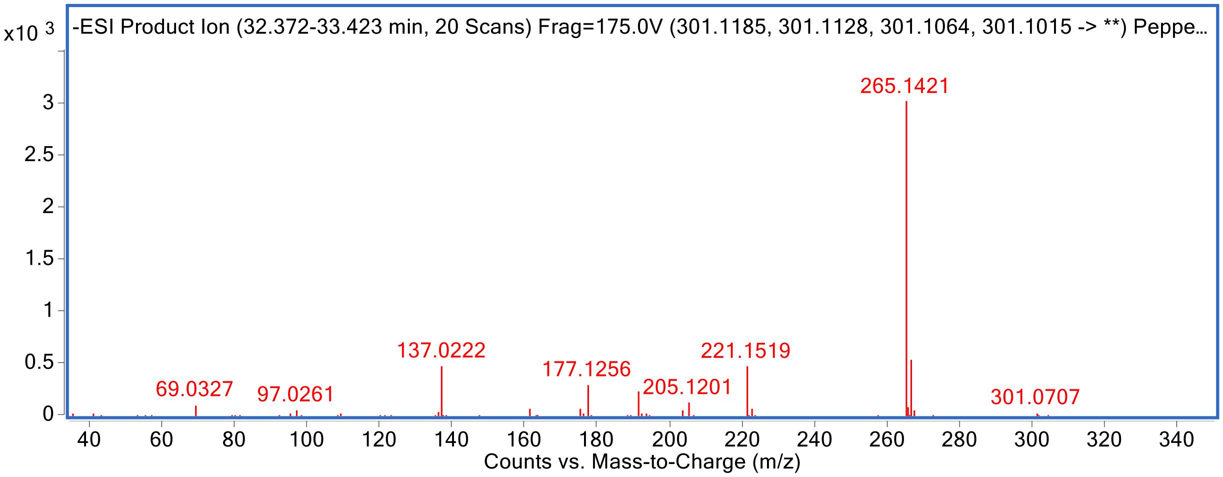

Chlorogenic acid

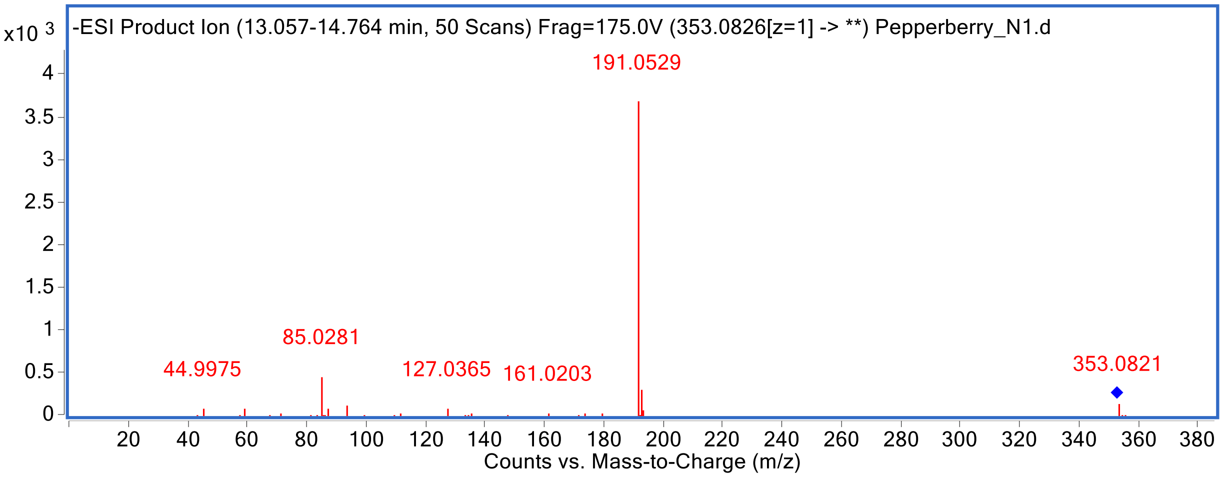

Glucosyringic acid

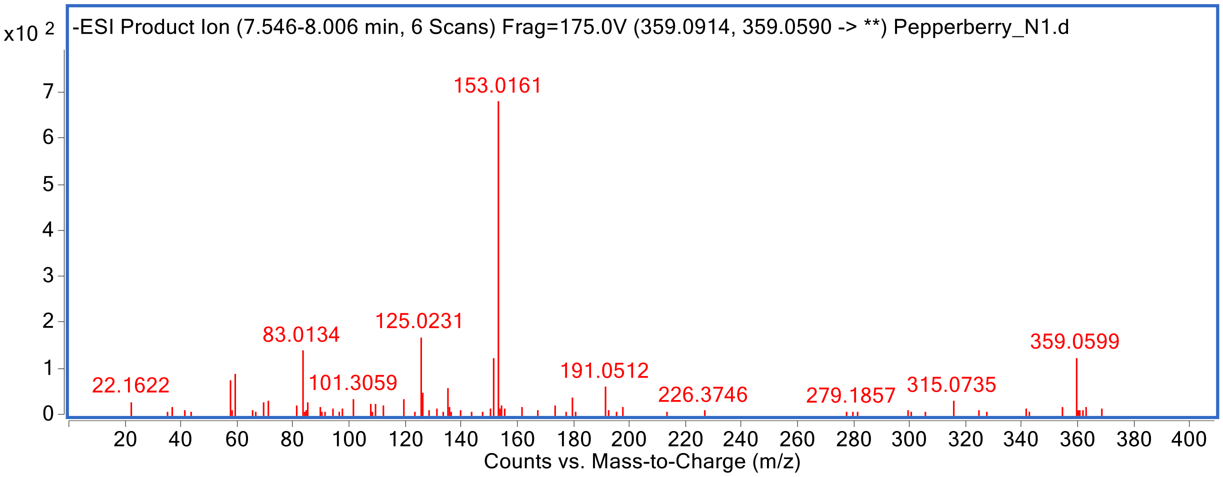

3-*p*-Coumaroylquinic acid

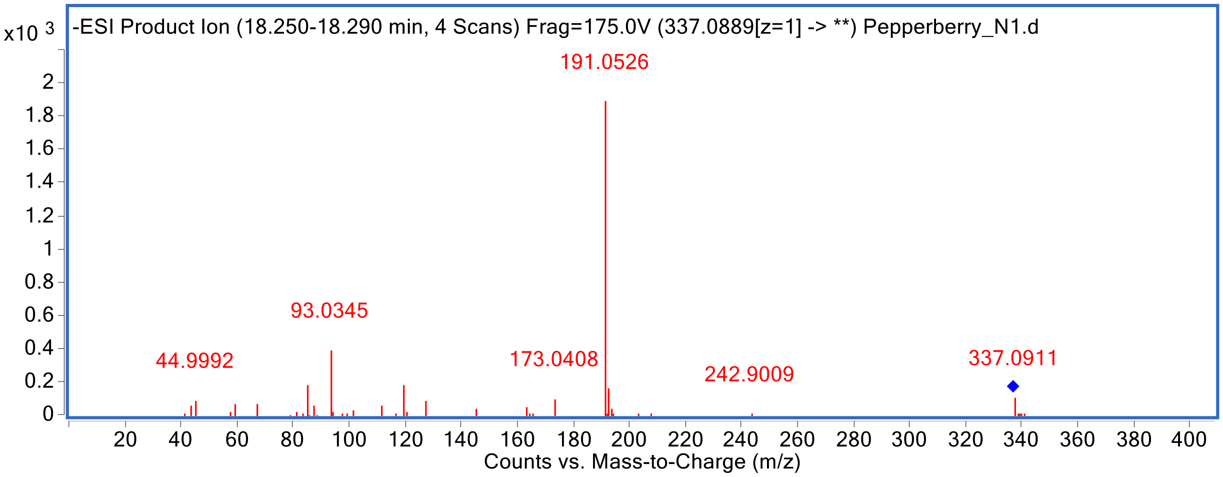

Naringenin-7-O-glucoside

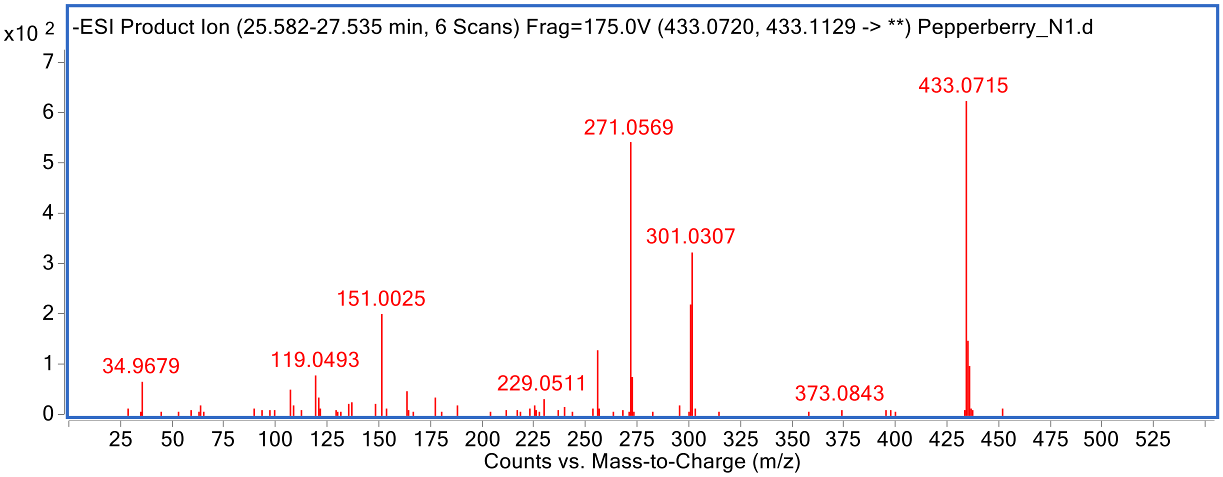

Kaempferol 3-*O*-glucoside

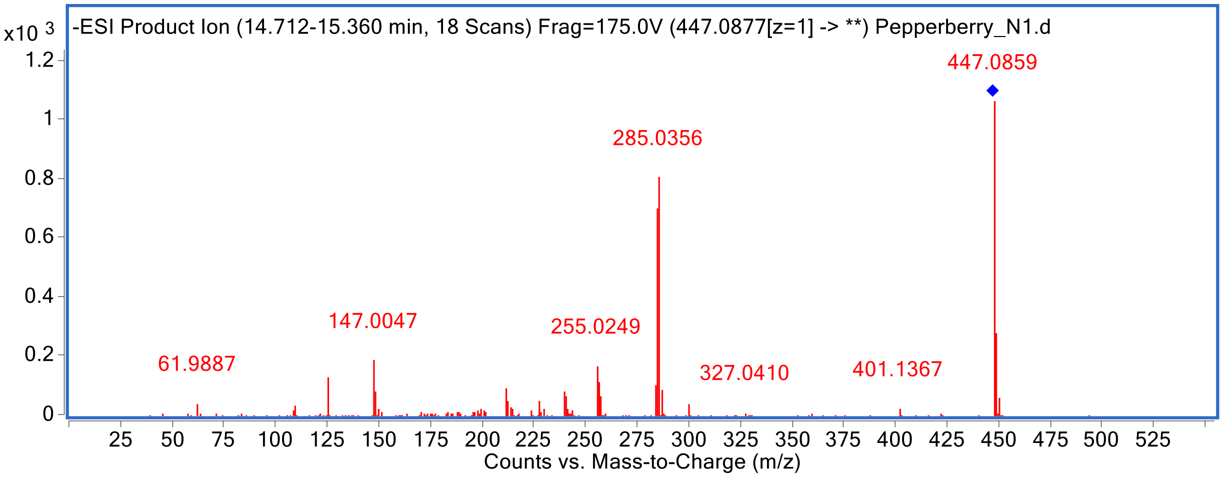

Azaleatin 3-arabinoside

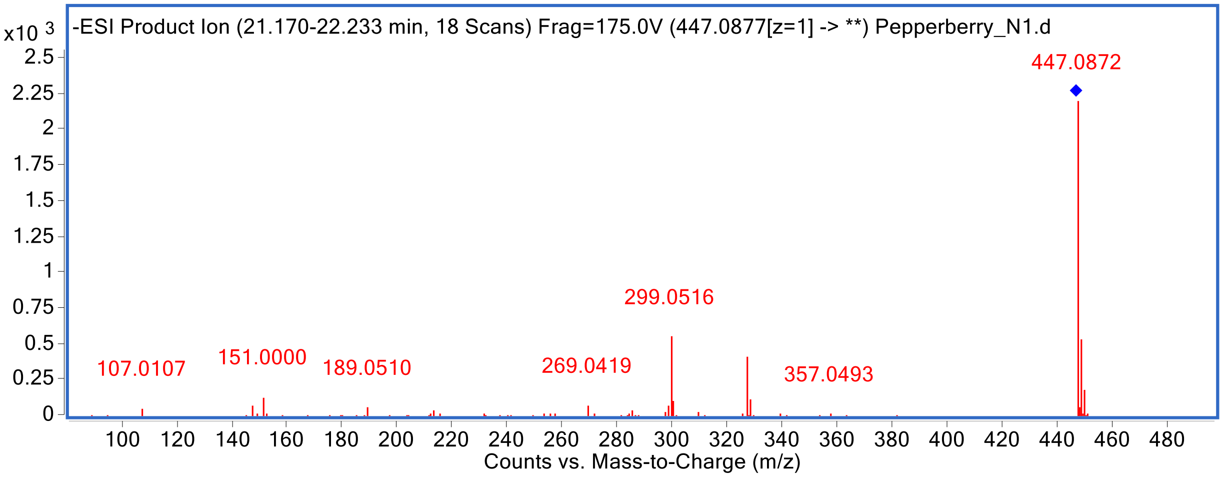

Quercetin-3-*O*-glucoside

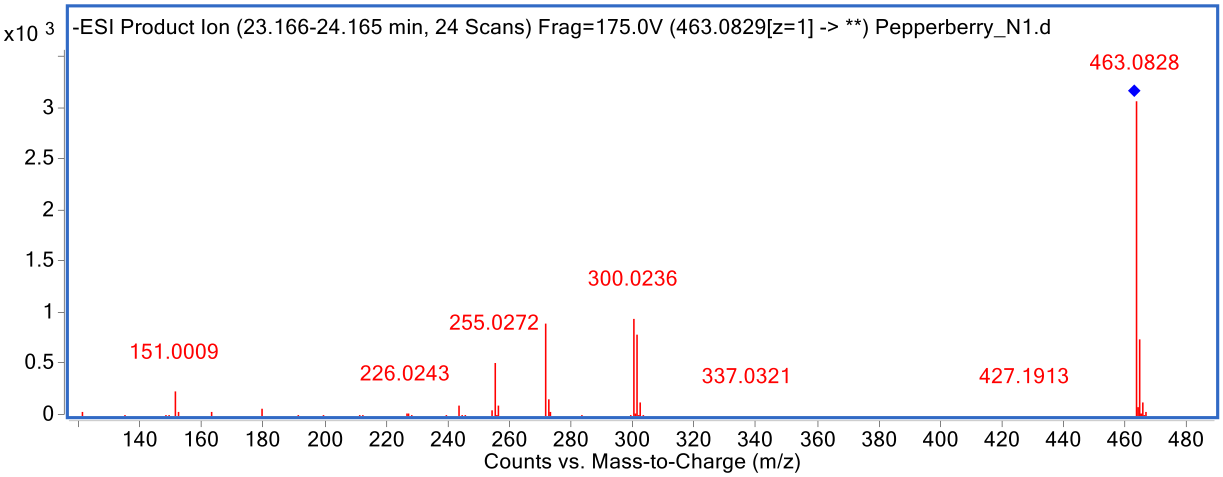

Procyanidin B2

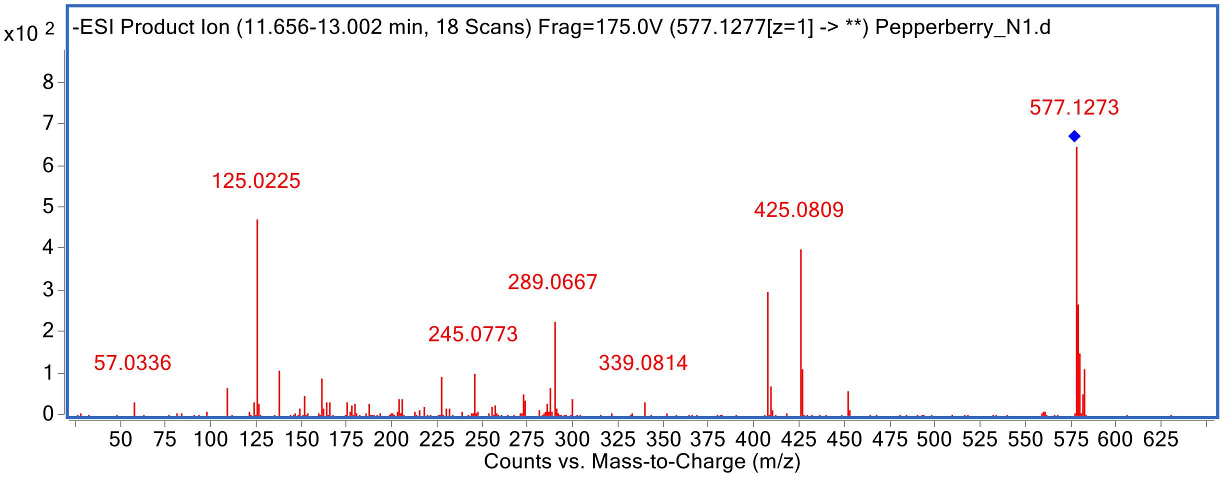

Procyanidin B2

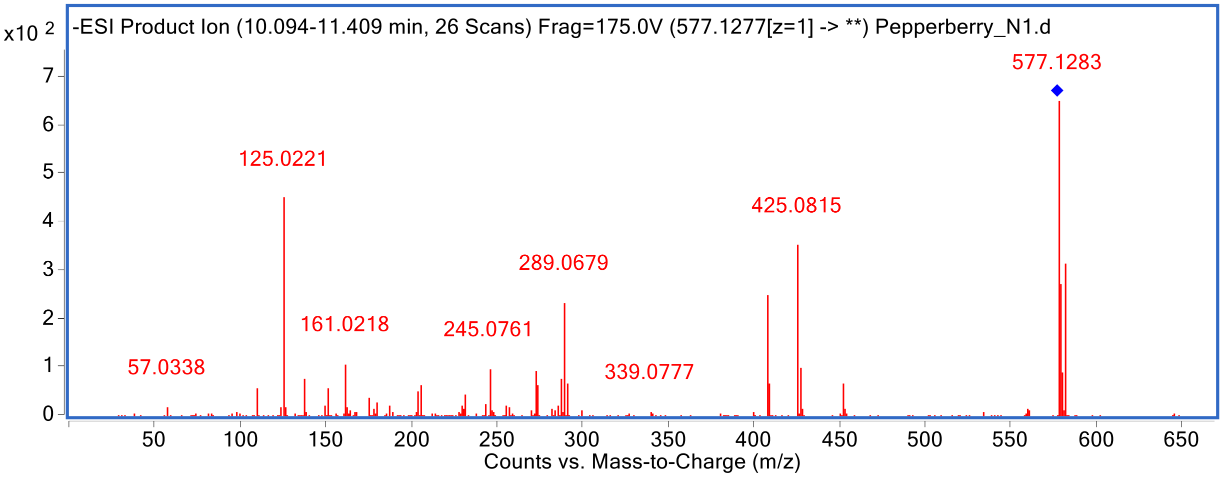

Kaempferol 3-rutinoside

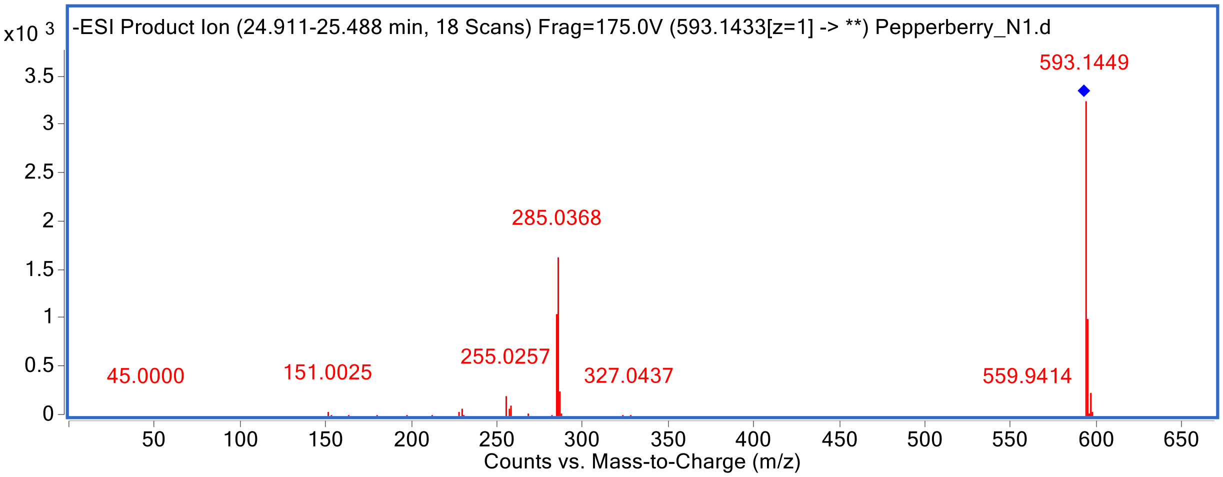

Rutin

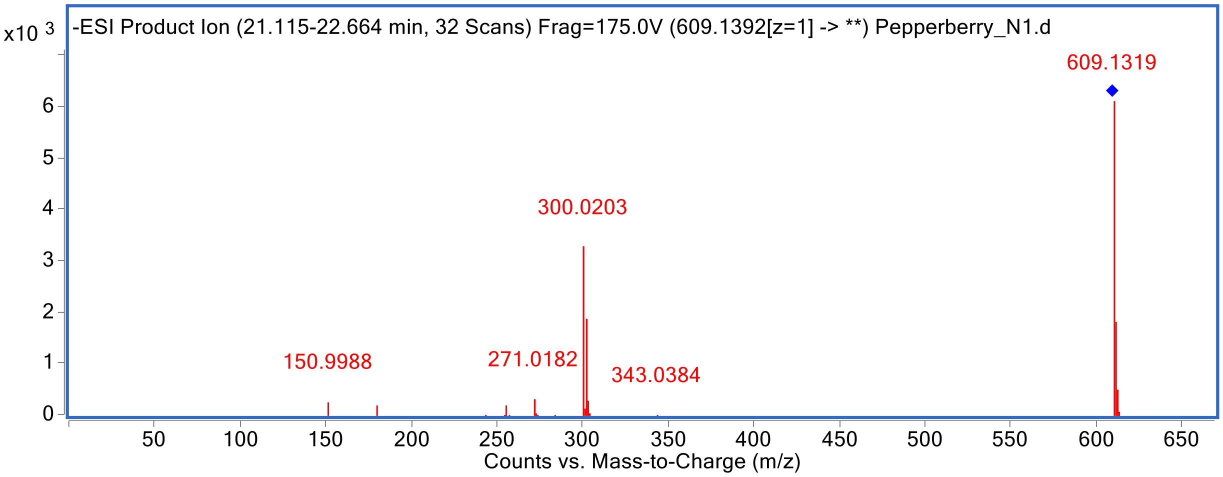

Protocatechuic acid 4-*O*-glucoside

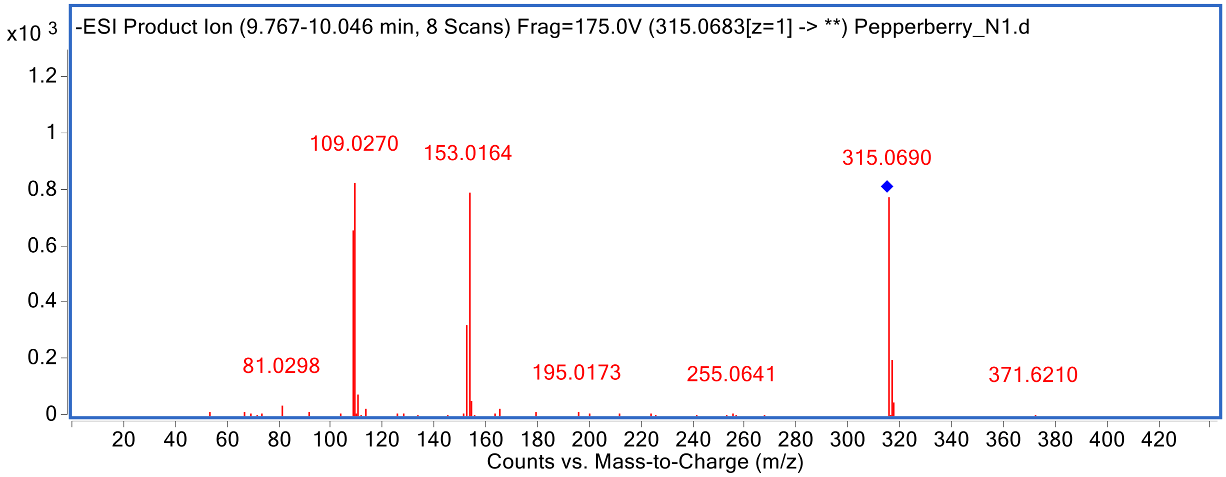

Pyrogallol

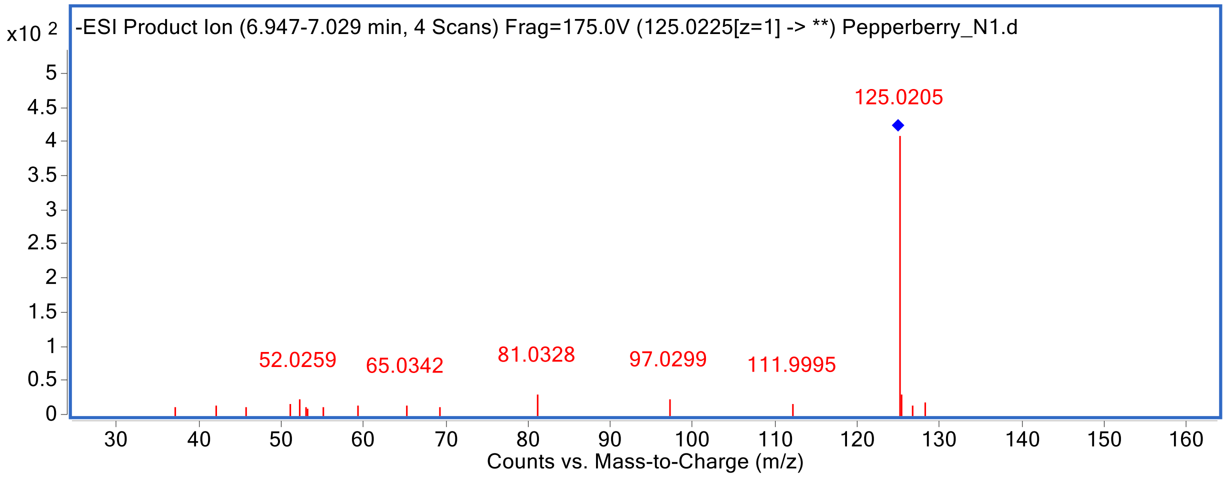

Gallic acid

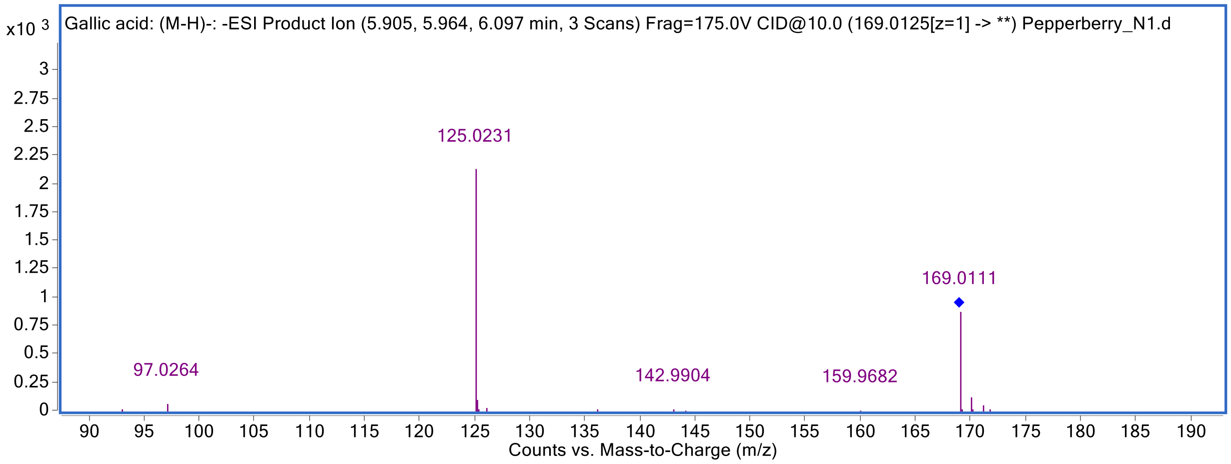

Quinic acid

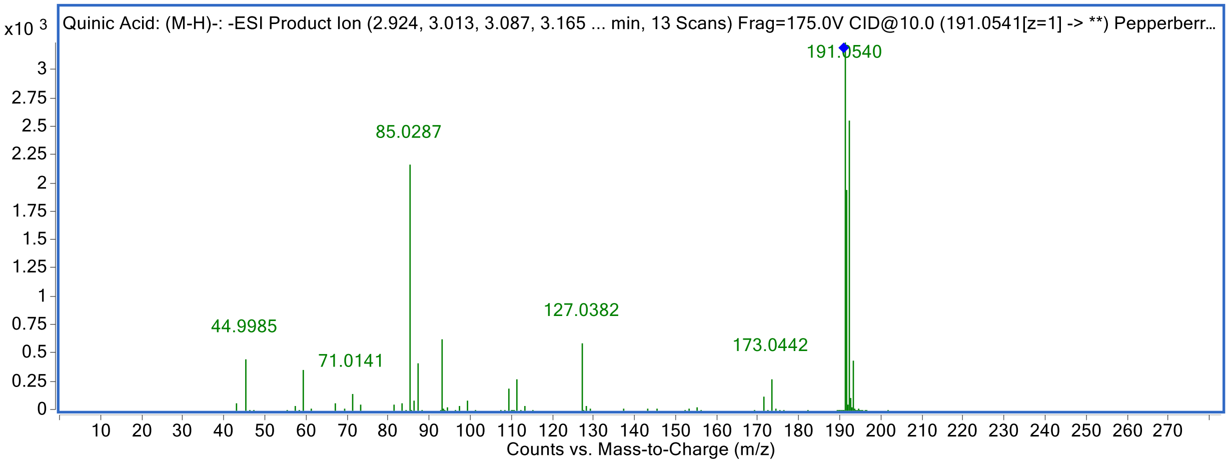

Quinic Acid: (M-H)-: -ESI Product Ion (2.924, 3.013, 3.087, 3.165 ... min, 13 Scans) Frag=175.0V CID@10.0 (191.0541[z=1] -> **) Pepperberr...
x10 3
191.0540
85.0287
127.0382
44.9985
71.0141
173.0442
Counts vs. Mass-to-Charge (m/z)

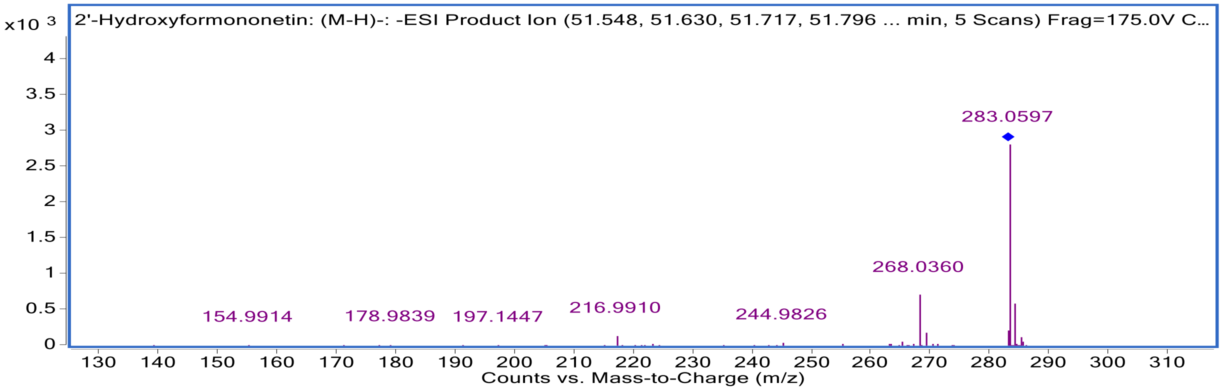

2'-Hydroxyformononetin: (M-H)-: -ESI Product Ion (51.548, 51.630, 51.717, 51.796 ... min, 5 Scans) Frag=175.0V C...
x10 3
283.0597
268.0360
154.9914
178.9839
197.1447
216.9910
244.9826
Counts vs. Mass-to-Charge (m/z)

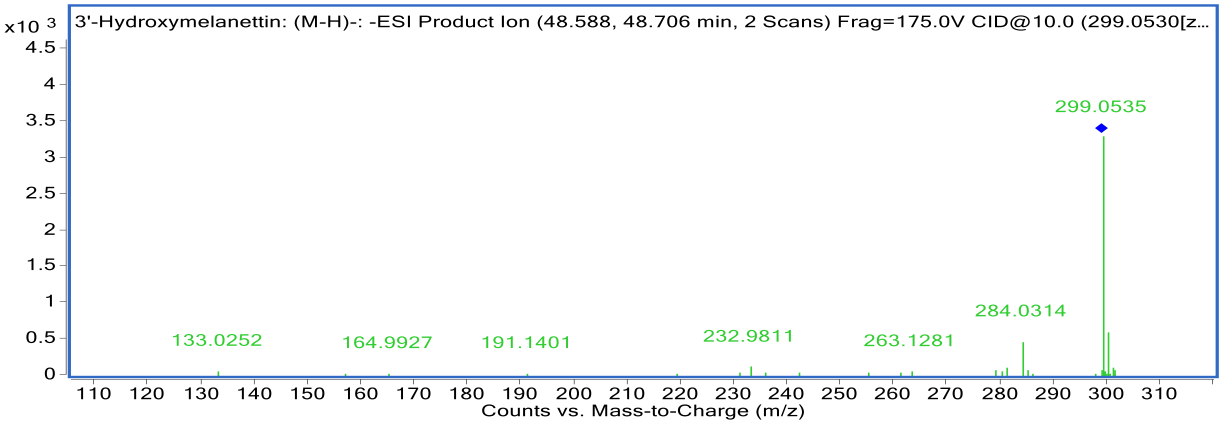

3'-Hydroxymelanettin: (M-H)-: -ESI Product Ion (48.588, 48.706 min, 2 Scans) Frag=175.0V CID@10.0 (299.0530[z...
x10 3
299.0535
284.0314
133.0252
164.9927
191.1401
232.9811
263.1281
Counts vs. Mass-to-Charge (m/z)

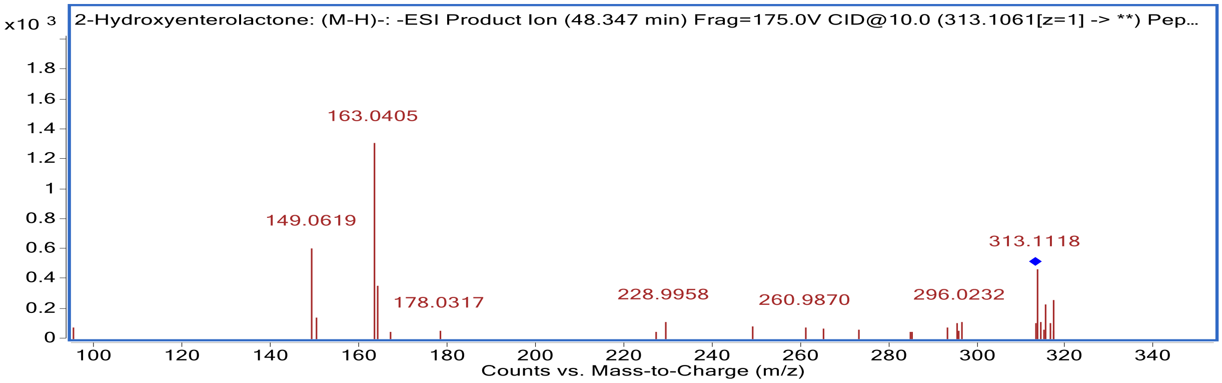

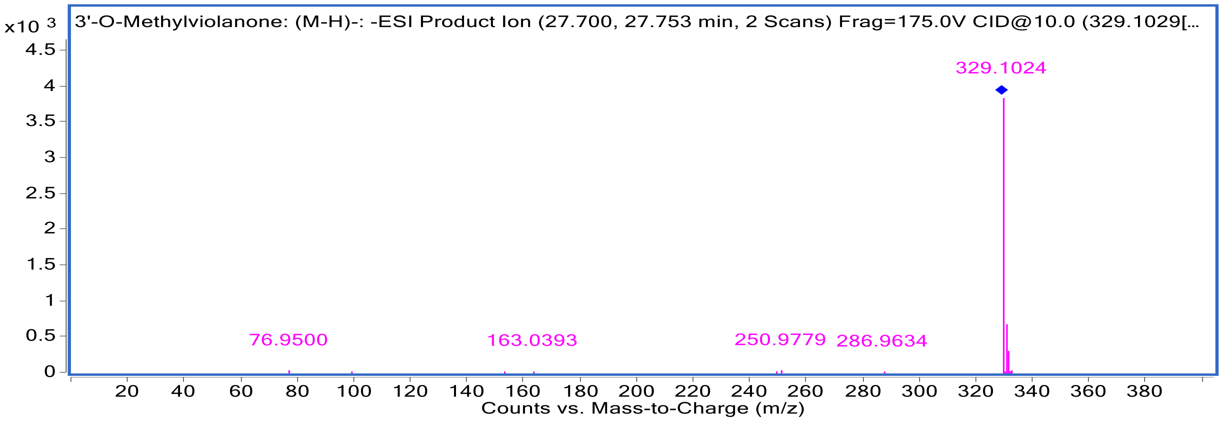

Kaempferol 3-*O*-glucoside

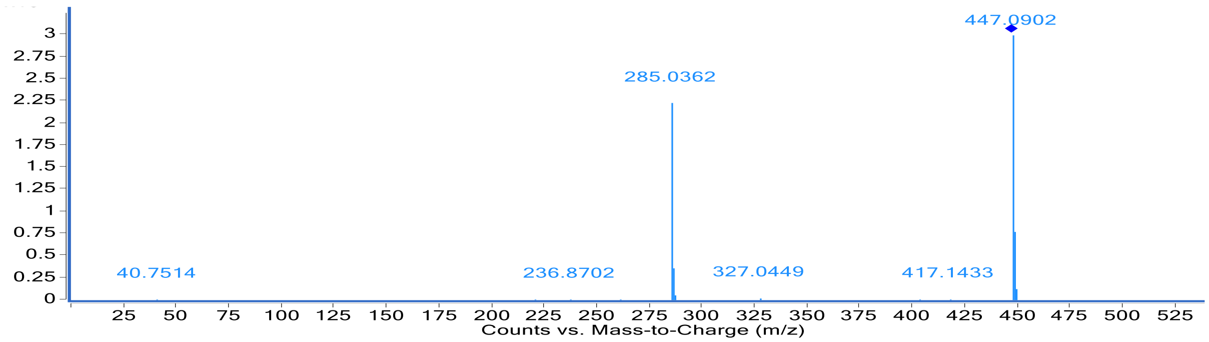

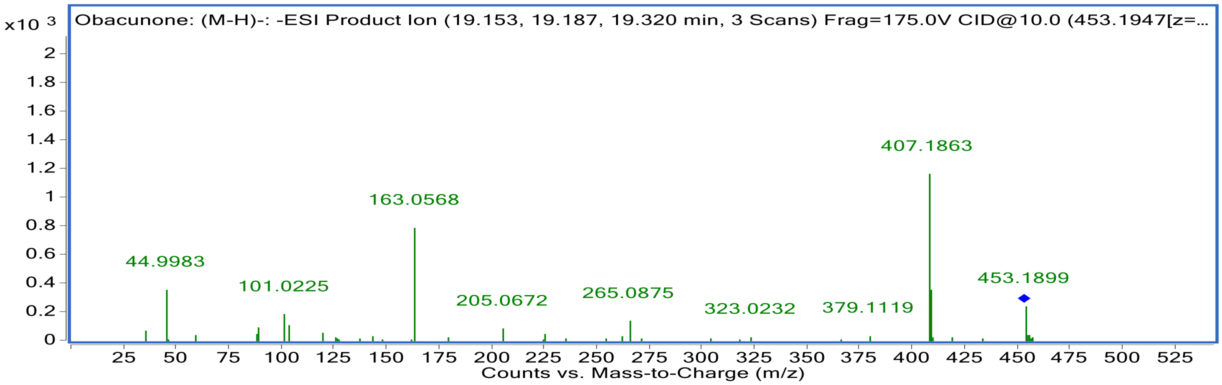

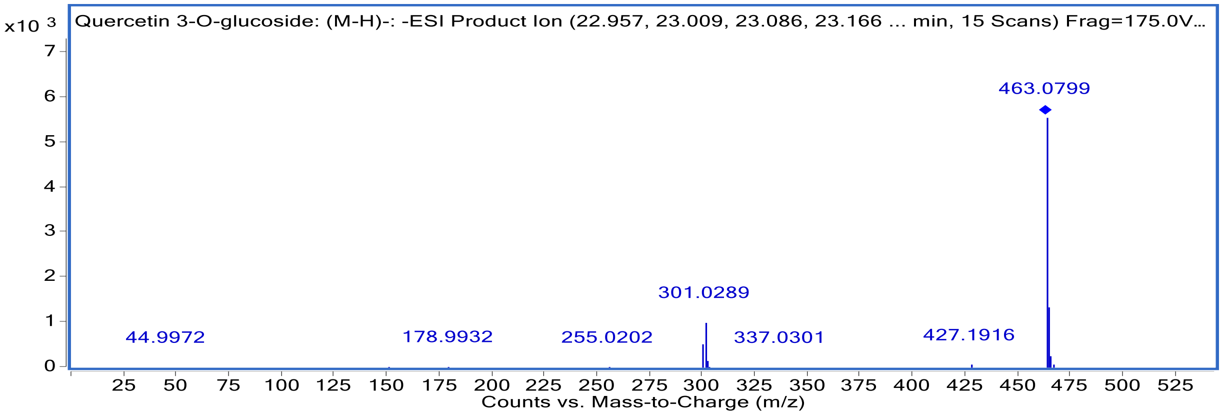

Silibinin

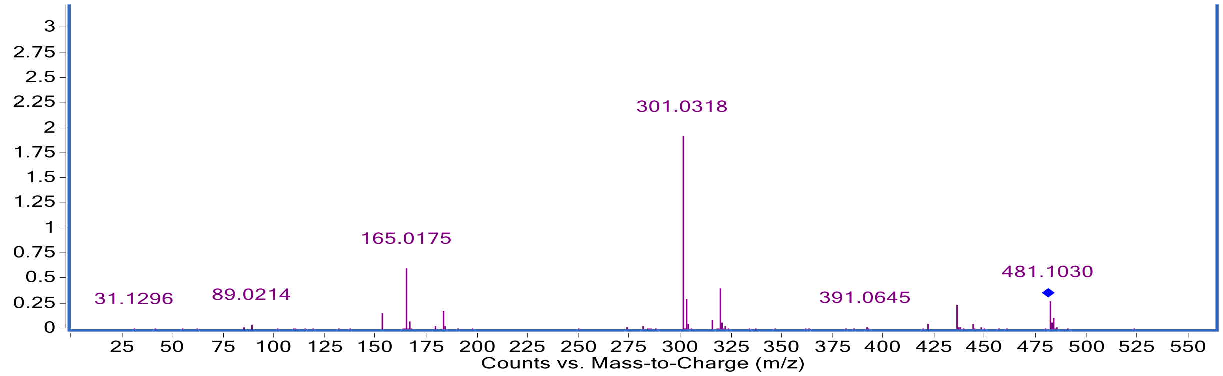

6-Hydroxykaempferol 3,6-diglucoside 7-glucuronide

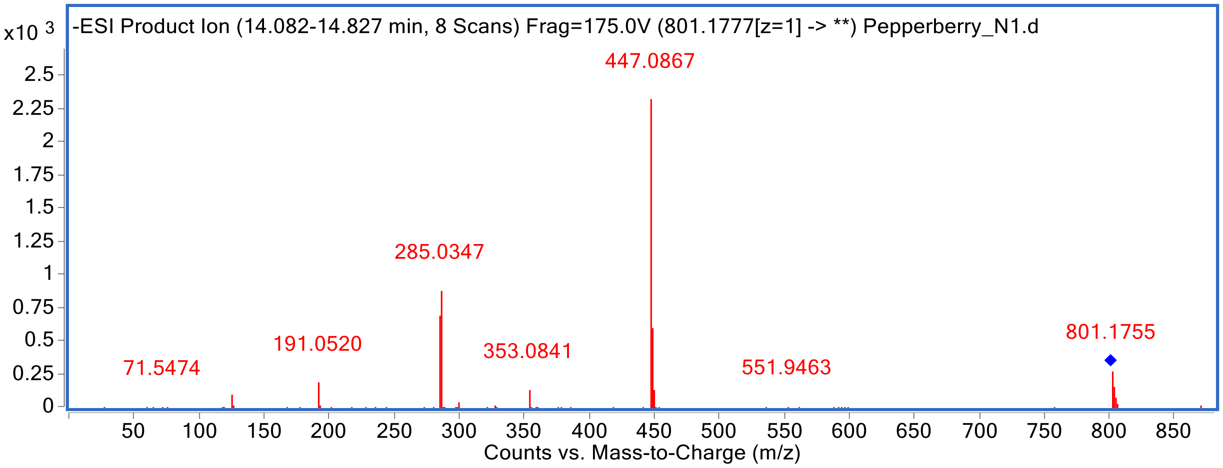

Figure S2. MS/MS spectra of some selected compounds

Quinic acid

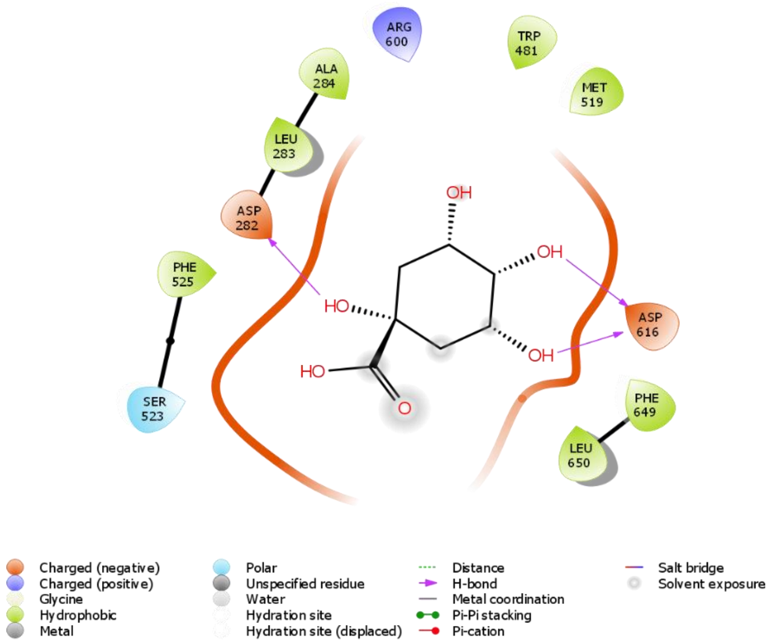

Naringin

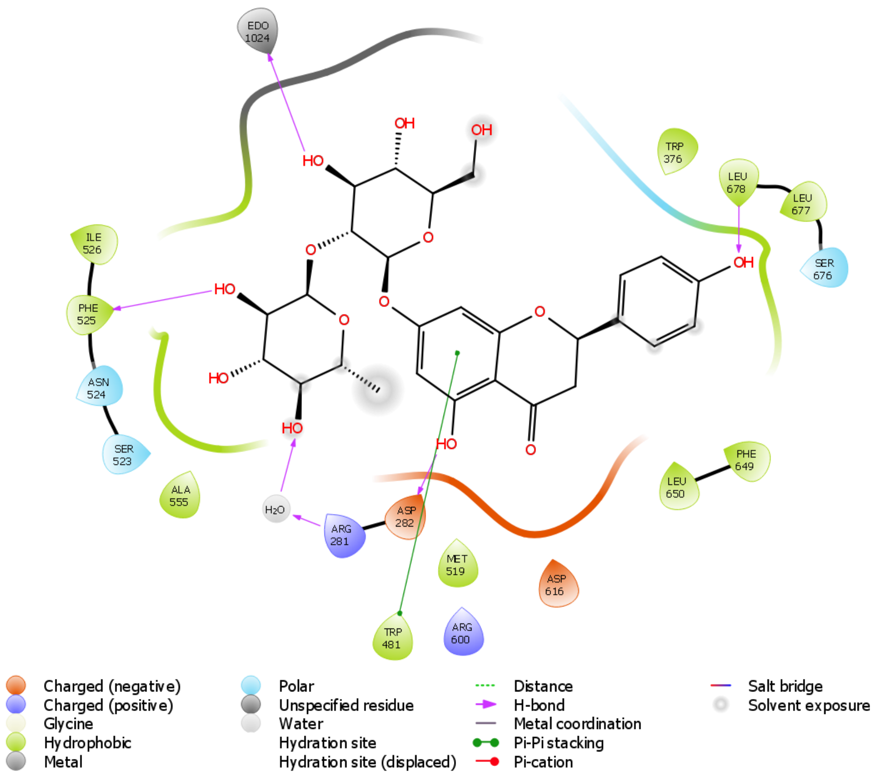

quercitrin

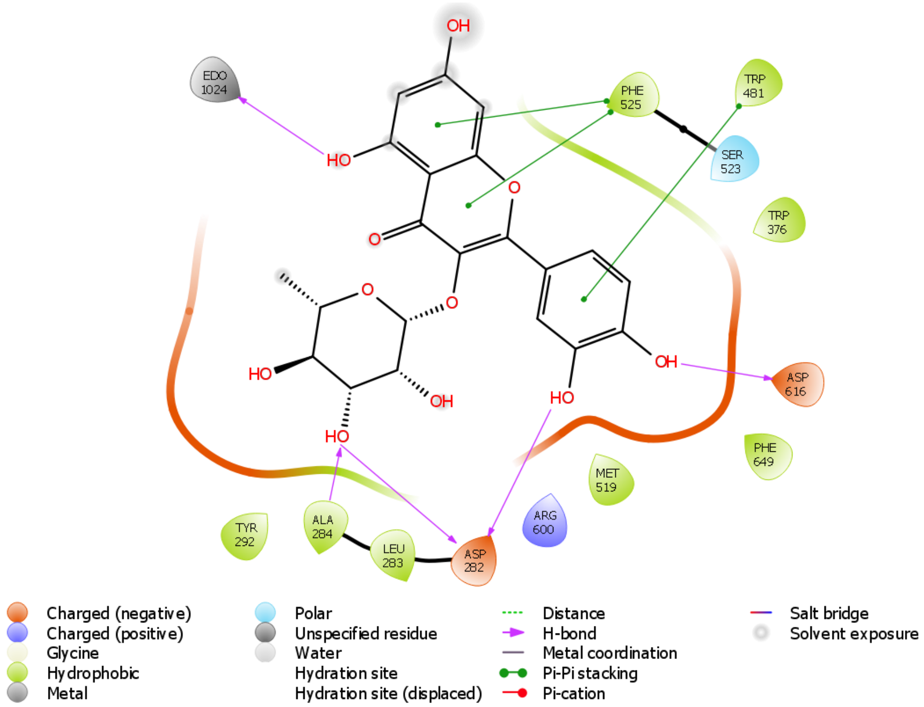

Diosmin

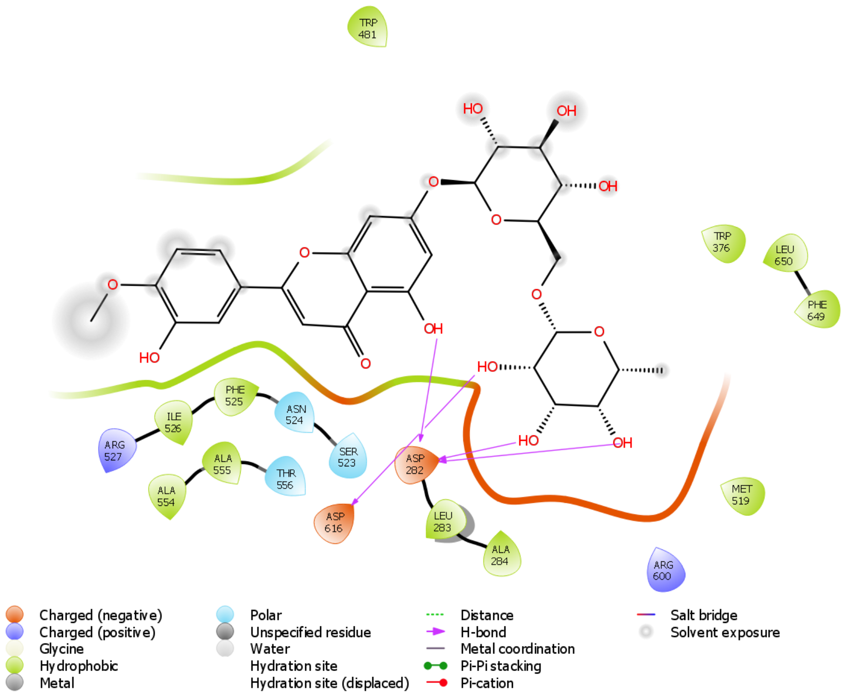

Procyanidin B2

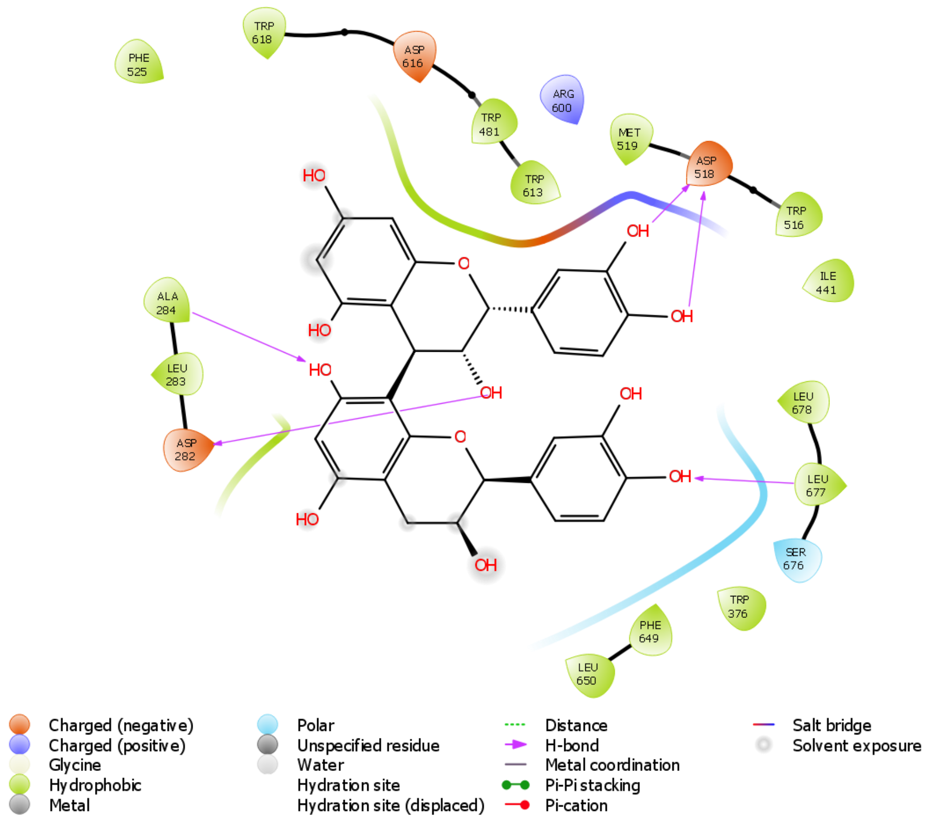

Acarbose

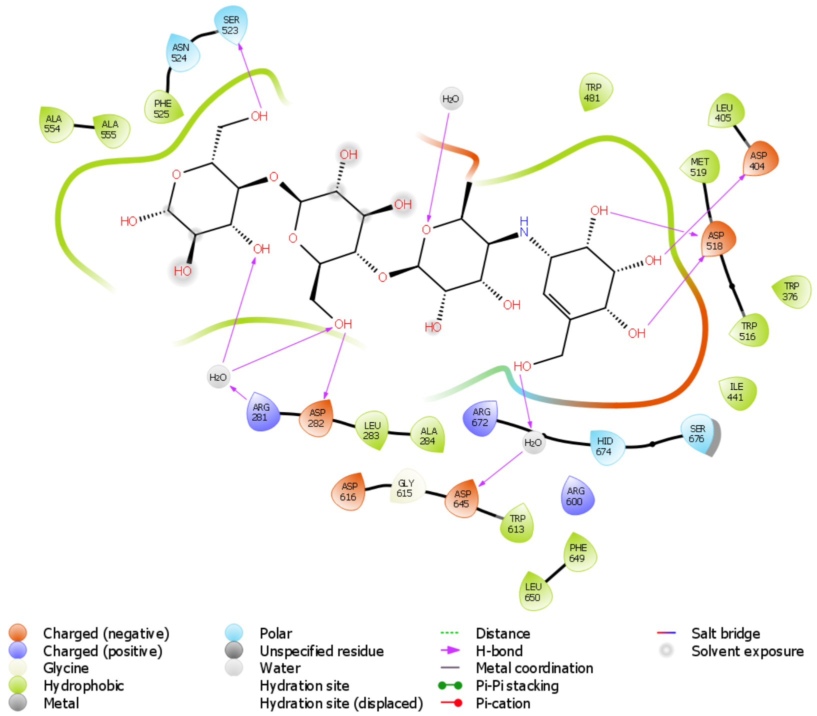

Rutin

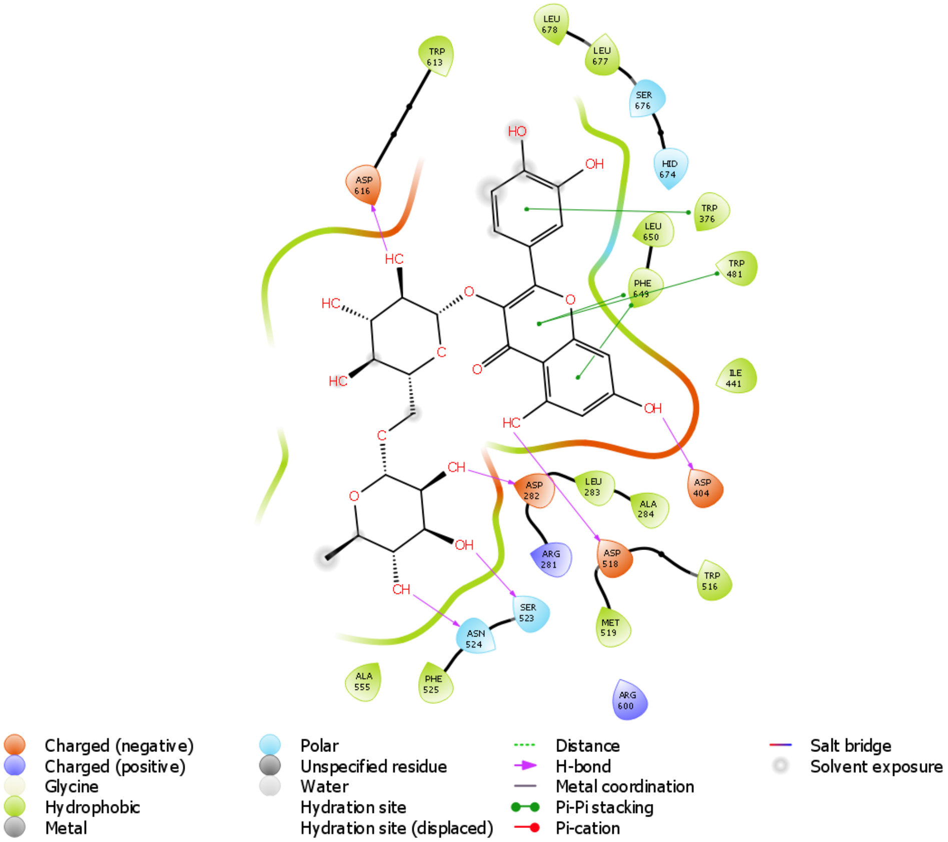

Delphinidin 3-sambubioside

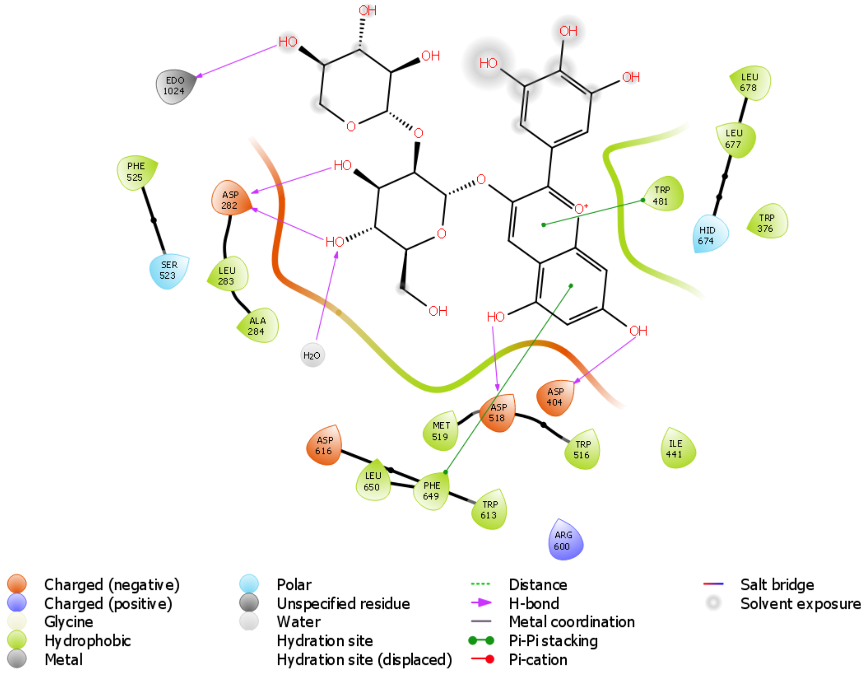

Delphinidin 3-rutinoside

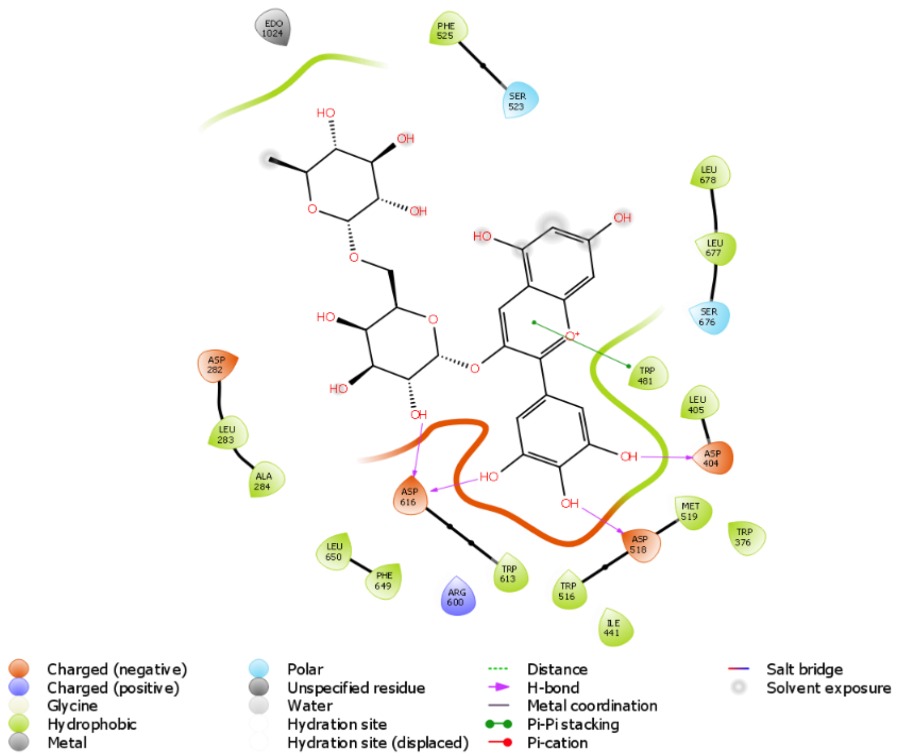

Quercetin 3-(2-galloylglucoside)

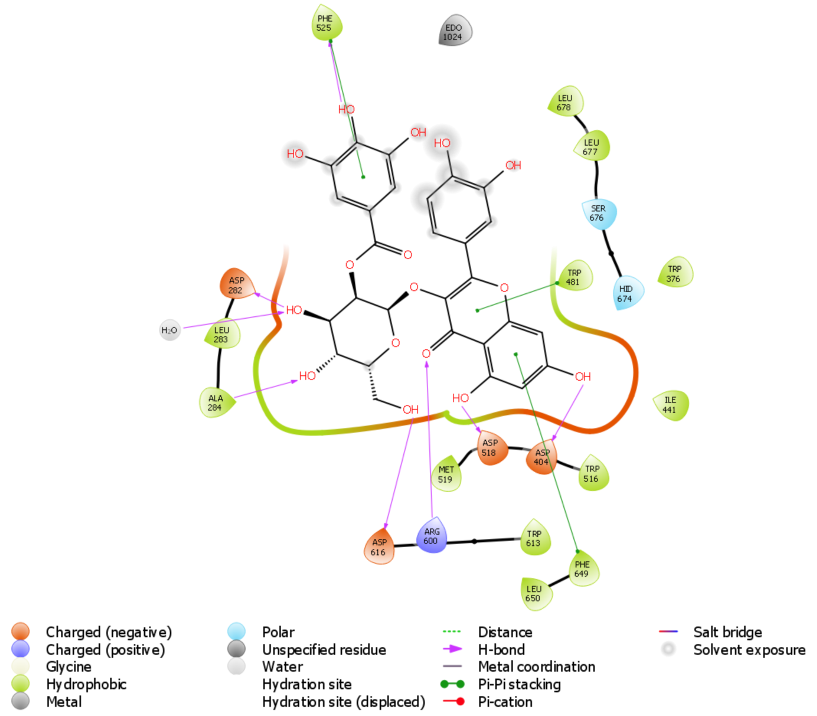

Figure S3. 2D binding geometry of some selected compounds

Table S1. Antioxidant activities of native Australian fruits and spices

	DPPH mg AAE/g	ABTS mg AAE/g	FRAP mg AAE/g	PMA mg AAE/g	FICA mg EDTA/g	•OH-RSA mg AAE/g	RPA mg AAE/g	α-glucosidase inhibition activity (IC_{50}) µg/mL
R	30.98 ± 1.64 ^{b}	59.27 ± 1.50 ^{c}	14.30 ± 1.92 ^{b}	13.41 ± 0.28 ^{b}	3.92 ± 0.42 ^{c}	39.31 ± 0.61 ^{c}	22.64 ± 1.78 ^{d}	79.09 ± 7.52 ^{b}
MPB	28.86 ± 0.49 ^{c}	85.60 ± 2.32 ^{b}	9.21 ± 1.03 c	34.19 ± 0.76 ^{a}	6.51 ± 0.20 ^{b}	43.25 ± 0.42 ^{b}	29.23 ± 3.82 ^{b}	24.04 ± 3.01 ^{d}
LA	24.94 ± 0.70 ^{d}	46.18 ± 0.38 ^{d}	4.60 ± 0.23 d	10.57 ± 0.18 ^{c}	3.60 ± 0.21 ^{c}	23.62 ± 0.47 ^{d}	26.79 ± 2.34 ^{c}	83.07 ± 9.03 ^{a}
SBG	49.70 ± 3.21 ^{a}	87.65 ± 3.17 ^{a}	26.57 ± 3.10 ^{a}	14.01 ± 0.47 ^{b}	9.05 ± 0.27 ^{a}	93.29 ± 2.20 ^{a}	60.54 ± 8.09 ^{a}	12.01 ± 1.2 ^{e}
Aca*								36.08 ± 4.15 ^{c}

* = Acarbose, Rosella (R), mountain pepper berries (MPB), lemon aspen (LA), strawberry gum (SBG); values with superscript letters ($^{a-e}$) are significantly different to each other.

Table S2. Quantification/semi-quantification of phenolic metabolites in Australian native fruits and spices (µg/g)

	**Name**	**Strawberry gum**	**Rosella**	**Mountain pepper berry**	**Lemon aspen**
1	Quercitrin	1274.04 ± 43.78	NQ	NQ	NQ
2	3',4',5'-Trimethoxyflavone	615.15 ± 21.63	NQ	NQ	NQ
3	Myricetin 3-*O*-rhamnoside	394.71 ± 16.21	NQ	NQ	NQ
4	Quercetin 3-*O*-arabinoside	371.54 ± 14.26	NQ	NQ	NQ
5	Quercetin 3-(2-galloylglucoside)	309.15 ± 20.38	NQ	NQ	NQ
6	* Epicatechin	279.17 ± 10.35	81.34 ± 4.64	121.24 ± 10.34	NQ
7	* Procyanidin B2	39.52 ± 3.65	NQ	NQ	11.32 ± 1.48
8	* Quercetin	53.04 ± 5.91	NQ	71.46 ± 4.52	18.31 ± 2.34
9	* Rutin	NQ	NQ	56.61 ± 5.48	NQ
10	* Isorhamnetin	12.52 ± 1.08	NQ	26.83 ± 2.86	19.04 ± 1.86
11	Myricetin	13.16 ± 0.89	NQ	23.67 ± 3.71	NQ
12	Quercetin-3-*O*-glucoside	17.83 ± 1.59	NQ	17.42 ± 2.14	NQ
13	* Chrysin	35.52 ± 2.77	NQ	NQ	NQ
14	Naringenin	24.72 ± 1.83	NQ	NQ	NQ
15	Delphinidin 3-*O*-sambubioside	NQ	196.61 ± 17.91	59.67 ± 5.24	NQ
16	Cyanidin-3-sambubioside	NQ	72.21 ± 8.63	NQ	NQ
17	* Cyanidin	NQ	53.04 ± 5.17	67.27 ± 4.89	NQ
18	Cyanidin 3-rutinoside	NQ	142.98 ± 13.01	82.91 ± 7.25	NQ
19	Cyanidin 3-*O*-glucoside	NQ	93.64 ± 8.04	39.07 ± 4.51	NQ
20	Delphinidin 3-rutinoside	NQ	17.23 ± 1.61	NQ	NQ
	Flavonoids	3440.07	657.05	566.15	48.67
21	* Chlorogenic acid	11.42 ± 1.31	64.07 ± 4.71	134.05 ± 12.67	28.31 ± 2.03
22	* Protocatechuic acid	63.56 ± 4.67	NQ	44.57 ± 5.82	NQ
23	* *p*-Hydroxybenzoic acid	NQ	11.74 ± 1.56	21.91 ± 3.41	NQ
24	* *p*-Coumaric acid	NQ	NQ	10.56 ± 1.35	13.07 ± 4.18
25	* Caffeic acid	15.51 ± 2.09	NQ	23.49 ± 1.92	18.64 ± 3.52
26	* Gallic acid	23.54 ± 3.19	17.21 ± 2.17	19.24 ± 3.12	NQ
	Phenolic acids				

* = quantified by using pure standards, NQ = Not quantified

Table S3. The calculated binding energies of selected compounds

No.	Compounds	Binding energy (Kcal/mol)
106	Quercetin 3-(2-galloylglucoside)	-11.09
107	Delphinidin 3-rutinoside	-11.08
108	Cyanidin 3-*O*-rutinoside	-10.90
109	Delphinidin 3-sambubioside	-10.38
110	Rutin	-10.14
111	Acarbose	-9.65
112	Cyanidin 3-rhamnoside 5-glucoside	-9.46
113	Cyanidin 3-*O*-galactoside	-9.38
114	Cyanidin 3-sambubioside	-9.37
115	acarbose	-9.29
116	Cyanidin 3-sambubioside	-9.26
117	Delphinidin 3-glucoside	-9.20
120	Delphinidin	-8.48
121	Cyanidin-3-glucoside	-8.19
122	Procyanidin B2	-8.05
123	Cyanidin	-7.81
125	Myricitrin	-7.59
126	3-Feruloylquinic acid	-7.32
127	Taxifolin	-7.13
128	Diosmin	-6.84
129	Quercitrin	-6.72
130	Chlorogenic acid	-6.62
131	Quercetin-3-*O*-arabinoside	-6.49
132	Naringin	-6.40
133	3-p-Coumaroylquinic acid	-6.35
134	Myricetin	-6.28
135	Quercetin	-5.95
136	Isorhamnetin	-5.68
137	Quinic acid	-5.65
138	Luteolin	-5.52
139	(-)-Epicatechin	-5.36
140	Hesperetin	-5.28
141	Gallic acid	-5.14
142	Quinic acid	-5.05
143	3-*O*-Sinapoylquinic acid	-4.91
144	Diosmetin	-4.36
145	Naringenin	-4.33
146	*p*-Hydroxybenzoic acid	-4.30
147	Salicylic acid	-4.20
148	Caffeic acid	-4.08
149	*p*-coumaric acid	-3.80
150	Pyrogallol	-3.74
151	Chrysin	-3.19
152	Protocatechuic acid	-3.12
153	3’-4’-5’-Trimethoxyflavone	-3.09
154	Cinnamic acid	-3.04
157	Coumarin	-1.93

Table S4. Predicted absorption and distribution of selected compounds

		Absorption							distribution			
No.	Compounds	WS	Caco2 permeability	Intestinal absorption (human) %	Skin Permeability	P-g substrate	P-g I inhibitor	P-g II inhibitor	VDss (human)	Fraction unbound (human)	BBB permeability	CNS permeability
1	3-*p*-Coumaroylquinic acid	-1.99	-0.66	43.9	-2.74	Yes	No	No	0.03	0.61	-1.16	-3.69
2	3-Sinapoylquinic acid	-2.55	-0.61	39.7	-2.74	Yes	No	No	-1.13	0.46	-1.40	-4.02
3	Caffeic acid	-2.33	0.63	69.4	-2.72	No	No	No	-1.1	0.53	-0.65	-2.61
4	Chlorogenic acid	-2.45	-0.84	36.4	-2.74	Yes	No	No	0.58	0.66	-1.41	-3.86
5	Cinnamic acid	-2.61	1.72	94.8	-2.70	No	No	No	-1.05	0.38	0.45	-1.83
6	Gallic Acid	-2.56	-0.08	43.4	-2.74	No	No	No	-1.86	0.62	-1.1	-3.74
7	*p*-Coumaric acid	-2.38	1.21	93.5	-2.72	No	No	No	-1.15	0.43	-0.23	-2.42
8	*p*-Hydroxybenzoic acid	-1.88	1.15	84.0	-2.72	No	No	No	-1.56	0.59	-0.33	-3.21
9	Protocatechuic acid	-2.07	0.49	71.1	-2.73	No	No	No	-1.3	0.65	-0.68	-3.31
10	Quinic acid	-1.12	-0.26	32.2	-2.74	No	No	No	-0.22	0.82	-0.89	-3.67
11	Cyanidin	-2.94	-0.35	87.3	-2.74	Yes	No	No	0.95	0.24	-1.23	-2.22
12	Cyanidin 3-O-rutinoside	-2.89	-1.09	23.1	-2.74	Yes	No	Yes	1.94	0.18	-2.15	-4.94
13	Cyanidin 3-O-glucoside	-2.93	0.06	45.4	-2.74	Yes	No	No	1.46	0.27	-1.71	-3.81
14	Delphinidin 3-rutinoside	-2.89	-1.27	10.8	-2.74	Yes	No	Yes	1.62	0.22	-2.5	-5.16
15	Cyanidin 3-sambubioside	-2.89	-0.98	13.9	-2.74	Yes	No	No	1.62	0.2	-2.06	-5.14
16	Delphinidin	-2.92	-0.83	77.4	-2.74	Yes	No	No	0.86	0.31	-1.62	-3.52
17	Delphinidin 3-O-sambubioside	-2.89	-1.17	1.6	-2.74	Yes	No	No	1.32	0.22	-2.41	-5.36
18	Cyanidin 3-glucoside	-2.93	0.06	45.4	-2.74	Yes	No	No	1.46	0.27	-1.71	-3.81
19	3'-O-Methylviolanone	-2.84	-1.32	23.3	-2.74	Yes	No	No	1.08	0.22	-2.43	-5.1
20	Chrysin	-3.54	0.95	93.8	-2.74	Yes	No	No	0.4	0.14	0.05	-1.91
21	Diosmin	-2.93	0.31	29.3	-2.74	Yes	No	No	1.43	0.11	-1.8	-4.84
22	Epicatechin	-3.12	-0.28	68.8	-2.74	Yes	No	No	1.03	0.24	-1.05	-3.3
23	Genistein	-3.6	0.90	93.4	-2.74	Yes	No	No	0.09	0.09	-0.71	-2.05
24	Isorhamnetin	-3.0	0.00	76.0	-2.74	Yes	No	No	1.12	0.09	-1.14	-3.19
25	Luteolin	-3.09	0.10	81.1	-2.74	Yes	No	No	1.15	0.17	-0.91	-2.25
26	Myricetin	-2.92	0.10	65.9	-2.74	Yes	No	No	1.32	0.24	-1.49	-3.71
27	Naringin	-2.92	-0.66	25.8	-2.74	Yes	No	No	0.62	0.16	-1.6	-4.77
28	Procyanidin B2	-2.89	-1.23	66.8	-2.74	Yes	Yes	Yes	-0.16	0.31	-1.94	-3.98
29	Phloretin	-3.08	-0.33	60.5	-2.74	Yes	No	No	0.77	0.25	-0.93	-2.54
30	Quercetin	-2.93	-0.23	77.2	-2.74	Yes	No	No	1.56	0.21	-1.1	-3.07
31	Quercetin-3-glucoside	-2.93	0.24	48.0	-2.74	Yes	No	No	1.85	0.23	-1.69	-4.09
32	Taxifolin	-3.04	0.92	64.7	-2.74	Yes	No	No	1.64	0.32	-0.73	-3.2
33	Rutin	-2.89	-0.95	23.6	-2.74	Yes	No	No	1.66	0.19	-1.9	-5.18
34	Diosmetin	-3.24	0.33	79.9	-2.74	Yes	No	No	0.71	0.07	-0.95	-2.32

35	Coumarin	-1.52	1.65	97.3	-1.92	No	No	No	-0.14	0.37	-0.01	-1.93
36	Pyrogallol	-1.41	1.12	83.6	-2.75	No	No	No	0.13	0.71	-0.44	-3.25
37	Isorhamnetin	-3.00	0.0	76.0	-2.74	Yes	No	No	1.12	0.09	-1.14	-3.19
38	Quercetin	-2.93	-0.23	77.2	-2.74	Yes	No	No	1.56	0.21	-1.1	-3.07
39	Quercitrin	-2.9	0.05	52.7	-2.74	Yes	No	No	1.52	0.13	-1.5	-4.16
40	3',4',5'-Trimethoxyflavone	-4.35	1.39	98.1	-2.56	No	Yes	Yes	-0.1	0.21	0.26	-2.12
41	Myricitrin	-2.89	-0.98	43.3	-2.74	Yes	No	No	1.55	0.18	-1.81	-4.38
42	Quercetin 3-O-arabinoside	-2.98	0.23	57.3	-2.74	Yes	No	No	1.75	0.23	-1.57	-3.91
43	Quercetin 3-(2-galloylglucoside)	-2.89	-1.83	31.9	-2.74	Yes	Yes	No	1.25	0.19	-2.62	-4.59
44	Naringenin	-3.22	1.03	91.3	-2.74	Yes	No	No	-0.02	0.06	-0.58	-2.22
45	[6]-Gingerol	-3.16	0.94	92.4	-2.82	Yes	No	No	0.52	0.26	-0.73	-2.79
46	Xanthohumol	-3.51	0.82	89.9	-2.74	Yes	Yes	Yes	0.39	0.09	-1.02	-2.22
47	Eriodictyol	-3.25	-0.09	74.7	-2.74	Yes	No	No	0.38	0.11	-0.83	-3.14
48	Limocitrin	-3.08	-0.14	78.4	-2.74	Yes	No	No	0.42	0.06	-1.34	-3.41

Intestinal absorption (human) (IA), P-glycoprotein substrate, P-glycoprotein I inhibitor, P-glycoprotein II inhibitor, Water solubility (log mol/L) (WS), Volume of distributions (VDss); blood-brain barrier (BBB); central nervous system (CNS)

Interpretation of results: If the Caco2 permeability value is higher than 0.90, a compound is considered to have a high Caco-2 permeability. A compound with a value of less than 30% is considered to be poorly absorbed in the human intestinal. If a compound has a value less than −2.5 is considered to have a low skin permeability. The compounds with values < −3 cannot penetrate the central nervous system while values > −2 are considered to penetrate the central nervous system. The compounds with values logBB < −1 are poorly distributed to the brain while values logBB > 0.3 are considered to cross the blood-brain barrier readily. VDss is considered low if below 0.71 L/kg (log VDss < −0.15) and high if above 2.81 L/kg (log VDss > 0.45).

Table S5. Pharmacokinetics properties of selected compounds

No.	Molecule	GI absorption	BBB permeant	Pgp substrate	CYP1A2 inhibitor	CYP2C19 inhibitor	CYP2C9 inhibitor	CYP2D6 inhibitor	CYP3A4 inhibitor	log Kp (cm/s) skin permeation
1	3-p-Coumaroylquinic acid	Low	No	No	No	No	No	No	No	-8.41
2	3-Sinapoylquinic acid	Low	No	Yes	No	No	No	No	No	-8.82
3	Caffeic acid	High	No	No	No	No	No	No	No	-6.58
4	Chlorogenic acid	Low	No	No	No	No	No	No	No	-8.76
5	Cinnamic acid	High	Yes	No	No	No	No	No	No	-5.69
6	Gallic Acid	High	No	No	No	No	No	No	Yes	-6.84
7	*p*-Coumaric acid	High	Yes	No	No	No	No	No	No	-6.26
8	p-Hydroxybenzoic acid	High	Yes	No	No	No	No	No	No	-6.02
9	Protocatechuic acid	High	No	No	No	No	No	No	Yes	-6.42
10	Quinic acid	Low	No	Yes	No	No	No	No	No	-9.15
11	Cyanidin	High	No	Yes	Yes	No	No	No	No	-7.51
12	Cyanidin 3-O-rutinoside	Low	No	No	No	No	No	No	No	-11.84
13	Cyanidin 3-O-glucoside	Low	No	No	No	No	No	No	No	-8.94
14	Delphinidin 3-rutinoside	Low	No	No	No	No	No	No	No	-12.19
15	Cyanidin 3-sambubioside	Low	No	No	No	No	No	No	No	-10.83
16	Delphinidin	High	No	Yes	Yes	No	No	No	No	-7.86
17	Delphinidin 3-O-sambubioside	Low	No	No	No	No	No	No	No	-11.19
18	Cyanidin 3-glucoside	Low	No	No	No	No	No	No	No	-8.94
19	3-O-Methylviolanone	Low	No	No	No	No	No	No	No	-11.04
20	Chrysin	High	Yes	No	Yes	No	No	Yes	Yes	-5.35
21	Diosmin	Low	No	Yes	No	No	No	No	No	-9.91
22	Epicatechin	High	No	Yes	No	No	No	No	No	-7.82
23	Genistein	High	No	No	Yes	No	No	Yes	Yes	-6.05
24	Isorhamnetin	High	No	No	Yes	No	No	Yes	Yes	-6.90
25	Luteolin	High	No	No	Yes	No	No	Yes	Yes	-6.25
26	Myricetin	Low	No	No	Yes	No	No	No	Yes	-7.40
27	Naringin	Low	No	Yes	No	No	No	No	No	-10.15
28	Procyanidin B2	Low	No	No	No	No	No	No	Yes	-8.15
29	Phloretin	High	No	No	Yes	No	Yes	No	Yes	-6.11
30	Quercetin	High	No	No	Yes	No	No	Yes	Yes	-7.05
31	Quercetin-3-glucoside	Low	No	No	No	No	No	No	No	-8.88
32	Taxifolin	High	No	No	No	No	No	No	No	-7.48
33	Rutin	Low	No	Yes	No	No	No	No	No	-10.26
34	Diosmetin	High	No	No	Yes	No	Yes	Yes	Yes	-5.93
35	Coumarin	High	Yes	No	Yes	No	No	No	No	-6.20
36	Pyrogallol	High	Yes	No	No	No	No	No	Yes	-6.70

37	Isorhamnetin	High	No	No	Yes	No	No	Yes	Yes	-6.90
38	Quercetin	High	No	No	Yes	No	No	Yes	Yes	-7.05
39	Quercitrin	Low	No	No	No	No	No	No	No	-8.42
40	3-4-5-Trimethoxyflavone	High	Yes	Yes	No	No	No	Yes	Yes	-6.57
41	Myricitrin	Low	No	No	No	No	No	No	No	-8.77
42	Quercetin 3-O-arabinoside	Low	No	No	No	No	No	No	No	-8.25
43	Quercetin 3-(2-galloylglucoside)	Low	No	No	No	No	No	No	No	-8.97
44	Naringenin	High	No	Yes	Yes	No	No	No	Yes	-6.17
45	[6]-Gingerol	High	Yes	No	Yes	No	No	Yes	No	-6.14
46	Xanthohumol	High	No	No	Yes	No	Yes	No	Yes	-4.86
47	Eriodictyol	High	No	Yes	No	No	No	No	Yes	-6.62
48	Limocitrin	High	No	No	Yes	No	Yes	Yes	Yes	-6.67

Table S6. Radar bioavailability properties of selected compounds

No.	Compounds	MW	Rotatable bonds	XLOGP3	TPSA	ESOL Log S	Fraction Csp3
1	3-p-Coumaroylquinic acid	338.31	5	-0.07	144.52	-1.75	0.38
2	3-Sinapoylquinic acid	398.36	7	-0.13	162.98	-1.92	0.44
3	Caffeic acid	180.16	2	1.15	77.76	-1.89	0.00
4	Chlorogenic acid	354.31	5	-0.42	164.75	-1.62	0.38
5	Cinnamic acid	148.16	2	2.13	37.30	-2.37	0.00
6	Gallic Acid	170.12	1	0.7	97.99	-1.64	0.00
7	p-Coumaric acid	164.16	2	1.46	57.53	-2.02	0.00
8	p-Hydroxybenzoic acid	138.12	1	1.58	57.53	-2.07	0.00
9	Protocatechuic acid	154.12	1	1.15	77.76	-1.86	0.00
10	Quinic acid	192.17	1	-2.37	118.22	0.53	0.86
11	Cyanidin	287.24	1	0.77	114.29	-2.60	0.00
12	Cyanidin 3-O-rutinoside	595.53	6	-2.68	252.36	-1.73	0.44
13	Cyanidin 3-O-glucoside	449.38	4	0.14	193.44	-2.82	0.29
14	Delphinidin 3-rutinoside	611.53	6	-3.04	272.59	-1.60	0.44
15	Cyanidin 3-sambubioside	581.50	6	-1.39	252.36	-2.46	0.42
16	Delphinidin	303.24	1	0.41	134.52	-2.45	0.00
17	Delphinidin 3-O-sambubioside	597.50	6	-1.75	272.59	-2.33	0.42
18	Cyanidin 3-glucoside	449.38	4	0.14	193.44	-2.82	0.29
19	3-O-Methylviolanone	611.53	7	-1.42	261.59	-2.55	0.44
20	Chrysin	254.24	1	3.52	70.67	-4.19	0.00
21	Diosmin	608.54	7	0.14	238.20	-3.51	0.46
22	Epicatechin	290.27	1	0.36	110.38	-2.22	0.20
23	Genistein	270.24	1	2.67	90.90	-3.72	0.00
24	Isorhamnetin	316.26	2	1.87	120.36	-3.36	0.06
25	Luteolin	286.24	1	2.53	111.13	-3.71	0.00
26	Myricetin	318.24	1	1.18	151.59	-3.01	0.00
27	Naringin	580.53	6	-0.44	225.06	-2.98	0.52
28	Procyanidin B2	578.52	3	2.37	220.76	-5.14	0.20
29	Phloretin	274.27	4	2.63	97.99	-3.38	0.13
30	Quercetin	302.24	1	1.54	131.36	-3.16	0.00
31	Quercetin-3-glucoside	464.38	4	0.36	210.51	-3.04	0.29
32	Taxifolin	304.25	1	0.95	127.45	-2.66	0.13
33	Rutin	610.52	6	-0.33	269.43	-3.30	0.44
34	Diosmetin	300.26	2	3.1	100.13	-4.06	0.06
35	Coumarin	146.14	0	1.39	30.21	-2.29	0.00
36	Pyrogallol	126.11	0	0.52	60.69	-1.44	0.00
37	Isorhamnetin	316.26	2	1.87	120.36	-3.36	0.06

38	Quercetin	302.24	1	1.54	131.36	-3.16	0.00
39	Quercitrin	448.38	3	0.86	190.28	-3.33	0.29
40	3-4-5-Trimethoxyflavone	312.32	4	2.3	57.90	-3.48	0.17
41	Myricitrin	464.38	3	0.51	210.51	-3.20	0.29
42	Quercetin 3-O-arabinoside	434.35	4	0.98	190.28	-3.27	0.25
43	Quercetin 3-(2-galloylglucoside)	616.48	7	1.53	277.27	-4.53	0.21
44	Naringenin	272.25	1	2.52	86.99	-3.49	0.13
45	[6]-Gingerol	294.39	10	2.76	66.76	-2.96	0.59
46	Xanthohumol	354.40	6	5.07	86.99	-5.18	0.19
47	Eriodictyol	288.25	1	2.02	107.22	-3.26	0.13

(Lipophilicity: XLOGP3 between −0.7 and +5.0, size: MW between 150 and 500 g/mol, polarity: TPSA between 20 and 130Å^2, solubility: log S not higher than 6, saturation: fraction of carbons in the sp3 hybridization not less than 0.25, and flexibility: no more than nine rotatable bonds; topological polar surface area (TPSA), Only quinic acid, satisfied all parameters for drug-likeness (oral bioavailability).

Table S7. Metabolism and excretion of selected compounds

		Metabolism							Excretion	
No.	Compounds	CYP2D6 substrate	CYP3A4 substrate	CYP1A2 inhibitor	CYP2C19 inhibitor	CYP2C9 inhibitor	CYP2D6 inhibitor	CYP3A4 inhibitor	Total Clearance	Renal OCT2 substrate
1	3-*p*-Coumaroylquinic acid	No	No	No	No	No	No	No	0.45	No
2	3-Sinapoylquinic acid	No	No	No	No	No	No	No	0.73	No
3	Caffeic acid	No	No	No	No	No	No	No	0.51	No
4	Chlorogenic acid	No	No	No	No	No	No	No	0.31	No
5	Cinnamic acid	No	No	No	No	No	No	No	0.78	No
6	Gallic Acid	No	No	No	No	No	No	No	0.52	No
7	*p*-Coumaric acid	No	No	No	No	No	No	No	0.66	No
8	*p*-Hydroxybenzoic acid	No	No	No	No	No	No	No	0.59	No
9	Protocatechuic acid	No	No	No	No	No	No	No	0.55	No
10	Quinic acid	No	No	No	No	No	No	No	0.64	No
11	Cyanidin	No	No	Yes	No	No	No	No	0.53	No
12	Cyanidin 3-*O*-rutinoside	No	No	No	No	No	No	No	-0.27	No
13	Cyanidin 3-*O*-glucoside	No	No	No	No	No	No	No	0.52	No
14	Delphinidin 3-rutinoside	No	No	No	No	No	No	No	-0.41	No
15	Cyanidin 3-sambubioside	No	No	No	No	No	No	No	-0.24	No
16	Delphinidin	No	No	Yes	No	No	No	No	0.58	No
17	Delphinidin 3-*O*-sambubioside	No	No	No	No	No	No	No	-0.38	No
18	Cyanidin 3-glucoside	No	No	No	No	No	No	No	0.52	No
19	3'-*O*-Methylviolanone	No	No	No	No	No	No	No	-0.21	No
20	Chrysin	No	No	Yes	Yes	Yes	No	No	0.41	No
21	Diosmin	No	No	No	No	No	No	No	-0.11	No
22	Epicatechin	No	No	No	No	No	No	No	0.18	No
23	Genistein	No	No	Yes	Yes	No	No	No	0.15	No
24	Isorhamnetin	No	No	Yes	No	No	No	No	0.51	No
25	Luteolin	No	No	Yes	No	Yes	No	No	0.50	No
26	Myricetin	No	No	Yes	No	No	No	No	0.42	No
27	Naringin	No	No	No	No	No	No	No	0.32	No
28	Procyanidin B2	No	No	No	No	No	No	No	-0.09	Yes
29	Phloretin	No	No	No	No	No	No	No	0.21	No
30	Quercetin	No	No	Yes	No	No	No	No	0.41	No
31	Quercetin-3-glucoside	No	No	No	No	No	No	No	0.39	No
32	Taxifolin	No	No	No	No	No	No	No	-0.08	No
33	Rutin	No	No	No	No	No	No	No	-0.37	No
34	Diosmetin	No	No	Yes	Yes	Yes	No	No	0.60	No
35	Coumarin	No	No	Yes	No	No	No	No	0.97	No

36	Pyrogallol	No	No	No	No	No	No	No	0.10	No
37	Isorhamnetin	No	No	Yes	No	No	No	No	0.51	No
38	Quercetin	No	No	Yes	No	No	No	No	0.41	No
39	Quercitrin	No	No	No	No	No	No	No	0.36	No
40	3',4',5'-Trimethoxyflavone	No	Yes	Yes	Yes	Yes	No	No	0.42	Yes
41	Myricitrin	No	No	No	No	No	No	No	0.30	No
42	Quercetin 3-O-arabinoside	No	No	No	No	No	No	No	0.40	No
43	Quercetin 3-(2-galloylglucoside)	No	No	No	No	No	No	No	0.32	No
44	Naringenin	No	No	Yes	No	No	No	No	0.06	No
45	[6]-Gingerol	No	No	Yes	Yes	Yes	No	No	1.34	No
46	Xanthohumol	No	Yes	Yes	Yes	Yes	No	No	0.21	No
47	Eriodictyol	No	No	No	No	No	No	No	-0.01	No
48	Limocitrin	No	No	Yes	No	No	No	No	0.48	No

Organic cation transporter 2 (OCT2)

Table S8. Predicted toxicity of abundant phenolic compounds

No.	Compounds	AMES toxicity	MTD-H	hERG I inhibitor	hERG II inhibitor	ORAT (LD50)	LOAEL	Hepatotoxicity	Skin Sensitization	*T. pyriformis* toxicity	Minnow toxicity
1	3-*p*-Coumaroylquinic acid	No	-0.09	No	No	1.74	2.51	No	No	0.29	4.61
2	3-Sinapoylquinic acid	No	0.86	No	No	1.99	3.81	No	No	0.29	5.24
3	Caffeic acid	No	1.15	No	No	2.38	2.09	No	No	0.29	2.25
4	Chlorogenic acid	No	-0.13	No	No	1.97	2.98	No	No	0.29	5.74
5	Cinnamic acid	No	1.11	No	No	2.09	2.65	No	No	0.25	1.72
6	Gallic Acid	No	0.7	No	No	2.22	3.06	No	No	0.29	3.19
7	*p*-Coumaric acid	No	1.11	No	No	2.16	2.53	No	No	0.32	1.61
8	p-Hydroxybenzoic acid	No	0.85	No	No	2.26	2.48	No	No	0.27	1.81
9	Protocatechuic acid	No	0.81	No	No	2.42	2.02	No	No	0.27	2.45
10	Quinic acid	No	1.63	No	No	1.13	3.53	No	No	0.29	4.87
11	Cyanidin	No	0.5	No	No	2.46	2.54	No	No	0.29	2.55
12	Cyanidin 3-O-rutinoside	No	0.46	No	Yes	2.5	3.29	No	No	0.29	5.70
13	Cyanidin 3-O-glucoside	No	0.56	No	Yes	2.55	4.2	No	No	0.29	6.40
14	Delphinidin 3-rutinoside	No	0.44	No	Yes	2.49	3.47	No	No	0.29	6.84
15	Cyanidin 3-sambubioside	No	0.47	No	Yes	2.5	4.08	No	No	0.29	5.60
16	Delphinidin	No	0.51	No	No	2.55	2.93	No	No	0.29	3.31
17	Delphinidin 3-O-sambubioside	No	0.45	No	Yes	2.49	4.26	No	No	0.29	6.94
18	Cyanidin 3-glucoside	No	0.56	No	Yes	2.55	4.20	No	No	0.29	6.40
19	3'-O-Methylviolanone	No	0.44	No	Yes	2.48	4.41	No	No	0.29	10.88
20	Chrysin	No	0.02	No	No	2.29	0.96	No	No	0.54	1.75
21	Diosmin	No	0.57	No	Yes	2.51	3.34	No	No	0.29	5.35
22	Epicatechin	No	0.44	No	No	2.43	2.50	No	No	0.35	3.59
23	Genistein	No	0.48	No	No	2.27	2.19	No	No	0.38	1.94
24	Isorhamnetin	No	0.58	No	No	2.41	2.5	No	No	0.3	2.21
25	Luteolin	No	0.5	No	No	2.46	2.41	No	No	0.33	3.17
26	Myricetin	No	0.51	No	No	2.50	2.72	No	No	0.29	5.02
27	Naringin	No	0.43	No	Yes	2.5	4.2	No	No	0.29	6.04
28	Procyanidin B2	No	0.44	No	Yes	2.48	4.35	No	No	0.29	8.70
29	Phloretin	No	0.31	No	No	2.38	3.32	No	No	0.34	2.48
30	Quercetin	No	0.5	No	No	2.47	2.61	No	No	0.29	3.72
31	Quercetin-3-glucoside	No	0.57	No	Yes	2.54	4.42	No	No	0.29	8.06
32	Taxifolin	No	0.35	No	No	2.26	3.10	No	No	0.29	4.69
33	Rutin	No	0.45	No	Yes	2.49	3.67	No	No	0.29	7.68

34	Diosmetin	No	0.42	No	No	2.34	2.27	No	No	0.34	1.74
35	Coumarin	No	0.44	No	No	2.11	1.90	No	No	0.37	1.56
36	Pyrogallol	No	-0.27	No	No	2.05	2.37	No	No	0.13	2.73
37	Isorhamnetin	No	0.58	No	No	2.41	2.5	No	No	0.3	2.21
38	Quercetin	No	0.5	No	No	2.47	2.61	No	No	0.29	3.72
39	Quercitrin	No	0.5	No	No	2.59	3.02	No	No	0.29	4.95
40	3',4',5'-Trimethoxyflavone	No	0.28	No	No	2.2	1.20	No	No	0.48	-0.55
41	Myricitrin	No	0.45	No	Yes	2.54	3.39	No	No	0.29	6.00
42	Quercetin 3-O-arabinoside	No	0.58	No	No	2.54	3.85	No	No	0.29	6.67
43	Quercetin 3-(2-galloylglucoside)	No	0.44	No	Yes	2.49	4.65	No	No	0.29	10.11
44	Naringenin	No	-0.18	No	No	1.79	1.94	No	No	0.37	2.14
45	[6]-Gingerol	No	0.64	No	No	1.96	1.63	No	No	1.49	0.97
46	Xanthohumol	No	-0.03	No	Yes	2.18	1.91	No	No	0.54	0.54
47	Eriodictyol	No	0.01	No	No	2.03	2.48	No	No	0.33	2.97
48	Limocitrin	No	0.66	No	No	2.39	2.73	No	No	0.3	2.15

Oral Rat Acute Toxicity (ORAT), Oral Rat Chronic Toxicity (LOAEL), Max. tolerated dose (human) (MTD-H)

Explanations

Minnow toxicity

LC_{50} values below 0.5mM (log $LC_{50} < -0.3$) are regarded as high acute toxicity.

***T. Pyriformis* toxicity**

pIGC50 (negative logarithm of the concentration required to inhibit 50% growth in log µg/L) is considered, with a value > −0.5 log µg/L is considered toxic.

AMES toxicity: if the AMES value is positive, then the compound will be mutagenic.

Maximum tolerated dose: a value less than is equal to 0.477 log(mg/kg/day) is considered low and high if greater than 0.477 log(mg/kg/day).
